# Machine Learning−Accelerated Discovery of Proton-Conducting 2D Materials for Proton Exchange Membranes

**DOI:** 10.1021/acsnano.5c15161

**Published:** 2025-12-27

**Authors:** Yuting Li, Daniel Bahamon, Marcelo Lozada-Hidalgo, Nirpendra Singh, Andre K. Geim, Lourdes F. Vega

**Affiliations:** † Research & Innovation Center for Graphene and 2D Materials (RIC-2D), 105955 Khalifa University of Science and Technology, Abu Dhabi 127788, UAE; ‡ Research and Innovation Center on CO_2_ and Hydrogen (RICH Center) and Chemical and Petroleum Engineering Department, Khalifa University of Science and Technology, Abu Dhabi 127788, UAE; § Department of Physics and Astronomy and National Graphene Institute, 5292 University of Manchester, Manchester M13 9PL, U.K.; ∥ Physics Department, Khalifa University of Science and Technology, Abu Dhabi 127788, UAE

**Keywords:** proton permeation, ab-initio MD simulations, machine learning, 2D materials, proton exchange membranes for hydrogen fuel cells

## Abstract

Two-dimensional (2D) materials emerge as promising alternatives to conventional polymer-based proton exchange membranes (PEMs) due to their high proton conductivity, mechanical robustness, and surface tunability. Here we present an integrated framework combining ab initio molecular dynamics (AIMD) simulations and machine learning (ML) to accelerate the discovery of proton- and hydrogen-transport properties over 866 nonmetallic 2D materials. Three ML models were trained using AIMD-derived permeation barriers from 488 materials, with Random Forest achieving the highest accuracy and revealing structure–property relationships that govern proton transport. Critical descriptors, including proton−atom distance, pore size, interlayer spacing, and electron affinity, emerged as key predictors of permeation behavior. H^+^/H_2_ selectivity through additional AIMD simulations allowed identifying 18 promising candidates, including the experimentally studied graphene and hexagonal boron nitride, thus supporting the robustness of our approach. Experimentally synthesized but barely explored materials, including 2D Si, Ge, TeC, TeCl, GeSe and CSe, emerged as strong candidates for proton conducting membranes. The framework further highlights theoretically stable compounds as unexplored opportunities for PEMs. By integrating atomic-scale simulations with data-driven models, this work provides both fundamental insights into proton permeation mechanisms and practical guidance for designing selective, high-performance nanomaterials for hydrogen energy technologies.

## Graphical abstract

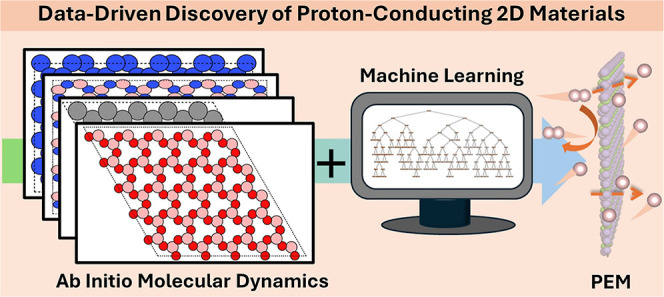

Proton Exchange Membranes (PEMs),^1,2^ also referred to as proton-conducting membranes or electrolytes, are materials specifically designed to facilitate selective proton (H^+^) transport while blocking electron and gas permeation. PEMs constitute the most essential component of fuel cells and redox flow batteries and are also employed in other electrochemical applications including PEM electrolyzers. Current PEM technologies face considerable limitations and would greatly benefit from enhanced proton conductivity under diverse operational conditions, reduced gas crossover and lower material costs - factors that collectively constraint their efficiency and commercial scalability.^3^ 2D materials^4,5^ represent a promising pathway to address these challenges, offering the potential for superior H^+^ permeability combined with exceptional H^+^/H_2_ selectivity that minimizes hydrogen gas crossover.^6–8^ Among these, nonmetallic 2D materials are particularly attractive due to their intrinsic ability to block electron transport.^9,10^ With hundreds of nonmetallic 2D crystals remaining unexplored in terms of their proton and gas permeability, abundant opportunities clearly exist for discovering novel proton-conducting materials with enhanced performance characteristics. To this end, conventional trial-and-error approaches to membrane design, characterization and testing create labor-intensive research cycles^11^ that are poorly suited to screening the vast space of possible 2D candidates. Given these constraints, establishing relationships between structural parameters, physicochemical properties and membrane performance becomes essential for rationalizing membrane design and accelerating materials discovery.

Theoretical and computational approaches are invaluable for predicting unknown properties, interpreting experimental data and understanding fundamental structure–property relationships; hence, they are considered powerful tools for designing novel materials for specific applications.^12–14^ Previously published work^9,15–18^ has demonstrated that the energy barrier for proton permeation is a critical parameter determining the efficiency of 2D materials as proton conductors. In general, proton permeation barriers in 2D materials can be determined through density functional theory (DFT),^16,18–20^ ab initio molecular dynamics (AIMD),^6,21–23^ and classical molecular dynamic (MD)^24^ simulations. For example, Hu et al.^17^ reported that graphene and hexagonal boron nitride (h-BN) monolayers are highly permeable to thermal protons under ambient conditions, with proton transport barriers ranging from 1.25 to 1.40 eV for graphene. Miao et al.^18^ investigated the permeability of a single graphene sheet to protons and hydrogen atoms by calculating the permeation energy barriers using DFT and harmonic transition state theory. Their results show that a proton penetrates more easily than hydrogen through a graphene sheet. Seel and Pandey^9^ studied the surface interactions and penetration barriers for the diffusion of protons and hydrogen atoms in various 2D materials, revealing that graphene and h-BN monolayers hold the potential for high selective permeability membranes. Moreover, Chai et al.^25^ used AIMD to screen proton transfer barriers for various undoped and nitrogen-doped GO membranes, revealing that N-doped GO achieves a proton transfer rate 7 orders of magnitude higher through a relay mechanism involving ketone and pyridine-like sites. This underscores the utility of AIMD in capturing detailed atomic interactions and dynamic processes crucial for understanding proton transport in complex materials.

Considering that quantum calculations are highly time-consuming, the integration of Machine learning (ML)^26,27^ with AIMD^28^ offers a promising approach for screening a large-scale number of 2D materials from available 2D-material databases to identify novel materials with enhanced proton permeation properties. Several online databases, such as Computational 2D Materials Database (C2DB),^29^ 2D Materials Encyclopedia (2Dmatpedia),^30^ and Materials Cloud 2D crystals database (MC2D),^31,32^ provide extensive resources of 2D materials, including crystal structure and energetic and electronic properties. Additionally, ML allows for rapidly predicting ion conductivity and permeation behavior and efficiently screening materials based on their fundamental properties and structural characteristics.^33^ For instance, Zhang et al.^34^ applied a fully connected neural network model to design anilinium-based anion exchange membrane (AEM) with enhanced conductivity by identifying structure–property relationships among 180,000 variants. Daglar and Keskin^35^ used grand canonical Monte Carlo (GCMC) and MD simulations to evaluate gas permeabilities and selectivities of metal−organic frameworks (MOFs) membranes, developing eight ML models to predict the adsorption and diffusion properties of He, H_2_, N_2_, and CH_4_ in 5249 MOFs and 31,494 MOF/polymer mixed matrix membranes with high accuracy, demonstrated on both experimental and hypothetical MOFs. Bai et al.^36^ also used eight supervised ML methods to predict H_2_ permeability and the trade-off of selectivity and permeability using the Gaussian Process Regression (GPR) and Random Forest (RF) methods, achieving the highest predictive performance, with the best R^2^ value reaching 0.88. Barnett et al.^37^ trained a GPR model using experimental data from 700 polymers, transforming each polymer into a binary ″fingerprint″ via RDKit.^38^ This enabled predictions for over 11,000 homopolymers, with synthesized top candidates surpassing the CO_2_/CH_4_ upper bound, highlighting the potential of ML-driven membrane design.

In this study, we explore the proton permeability of hundreds of nonmetallic 2D materials that have not yet been investigated for this application. Our approach (see Figure 1 and methodology section) starts by sourcing 866 nonmetallic 2D materials from the 2Dmatpedia database^30^ and calculating the proton permeation barrier for 488 materials using AIMD simulations. These AIMD-derived data are utilized to train ML models that predict proton permeation barriers for the entire database based on fundamental material properties such as structural, electronic, and energetic features. The trained models are subsequently used to evaluate the remaining 378 2D materials, predicting their permeability and corresponding energy barriers. We combine regression and classification approaches among three ML algorithms (deep neural networks (DNN), GPR, and RF). The best-performing model (RF) allows us to systematically explore correlations between proton permeation behavior and material characteristics. Following this approach, materials with proton permeation barriers below 1.4 eV (the reported value for pristine graphene, taken as a benchmark) are identified as promising candidates for further evaluation. These selected materials are then subjected to AIMD simulations to assess their H^+^/H_2_ selectivity, ensuring effective hydrogen gas blocking (H_2_ permeation barriers >10 eV). This approach accelerates the discovery of proton-conducting materials with negligible gas crossover for PEM applications.

**Figure 1:**
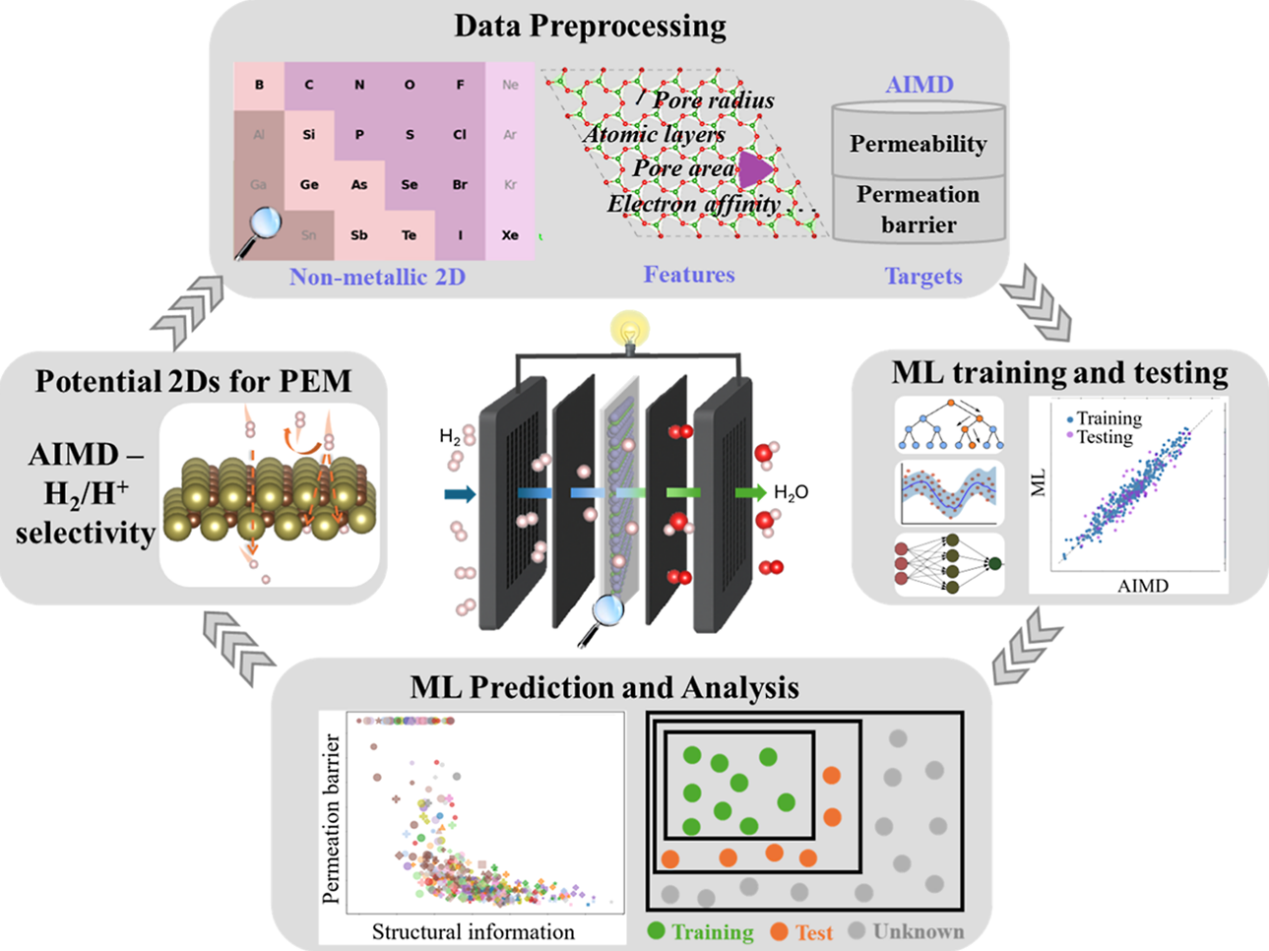
Schematic illustration of predicting permeation barriers and identifying the potential candidate of nonmetallic 2D materials for PEM via AIMD and ML methods. In the simplified periodic table, the nonmetallic and metalloid elements of the studied 2D materials are highlighted in bold font.

## Results and Discussion

### Data Collection and Mining

AIMD simulations were utilized to evaluate the proton permeation through 488 different nonmetallic 2D materials. For each material, three initial positions of proton permeation were considered, and the minimum energy barrier was selected as the representative proton permeation barrier for the specific material. Several representative energy profiles were used to calculate the proton permeation barrier through AIMD simulations, as shown in Figure S1 in the Supporting Information and Figure 2a. For example, Figure S1a illustrates a material that offers small resistance to proton permeation, where the proton passes directly through the monolayer with the lowest potential energy. In this case, the maximum energy is recorded when the proton reaches 1.4 Å below the surface, representing the energy barrier for proton desorption from the material. In contrast, Figure 2a depicts a situation where a significant energy barrier is observed as the proton passes the center of the 2D layer, corresponding to graphene. This maximum energy value is seen when the proton is positioned at the center of the ring, indicating significant resistance to proton transport in the central region of the material. Figure S1b in the Supporting Information depicts the calculations of a more complex multiatomic-layer 2D structure characterized by varying energy barriers along different segments of the proton pathway. The segment analysis also considers the energy required for the proton to reach 1.4 Å from the layer. The highest energy barrier among these segments is selected as the final permeation barrier for this material, representing the most difficult route for proton permeation in multilayered systems. Moreover, the permeation barrier for graphene (GN), shown in Figure 2a is 1.33 eV, while the obtained barrier for h-BN was 0.69 eV. These values are consistent with previously reported results in the literature,^17^ which indicate a permeation barrier of 1.25–1.40 eV for graphene and approximately 0.7 eV for monolayer h-BN.

**Figure 2:**
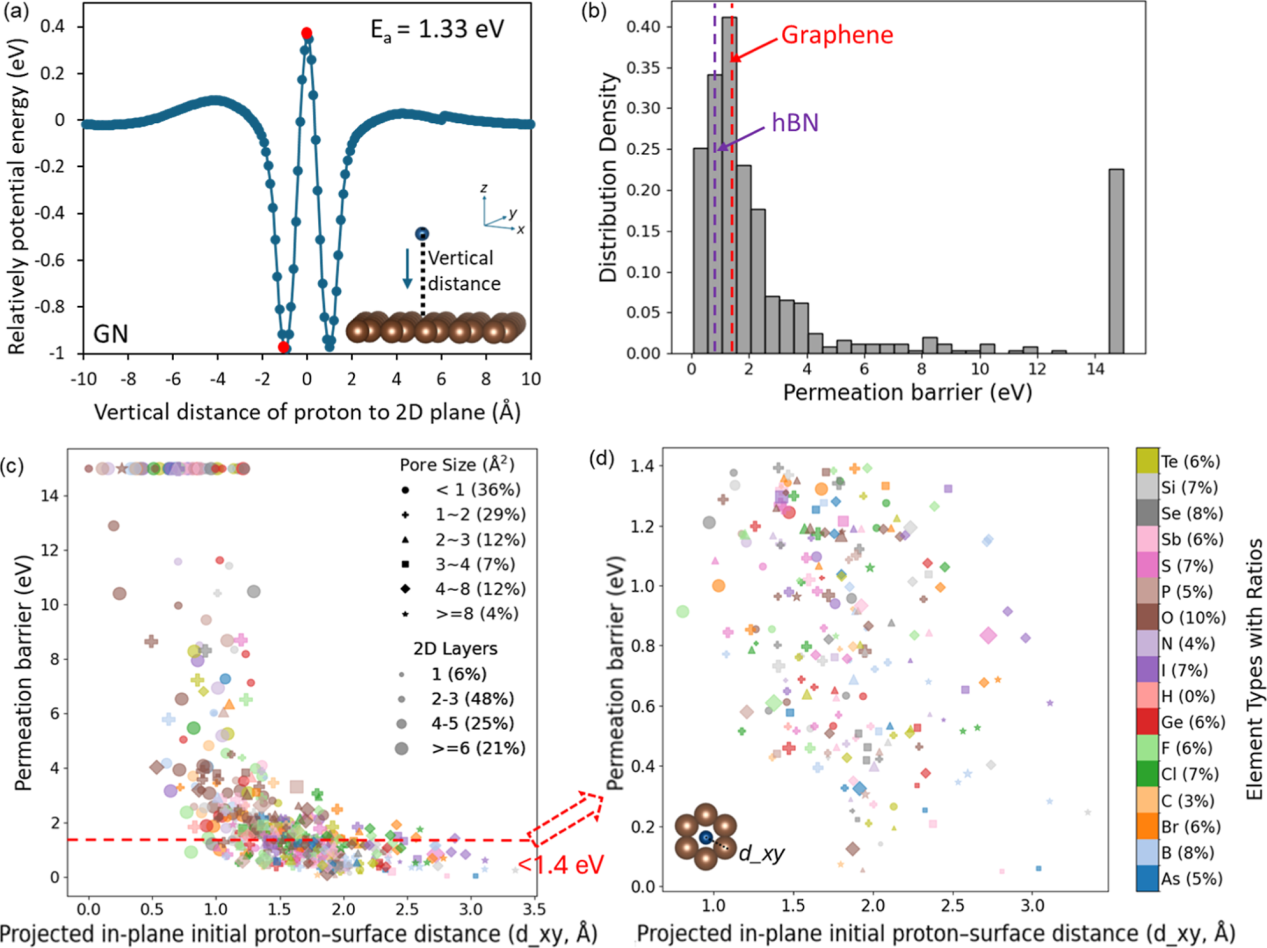
(a) Relative potential energy profile of proton permeation for graphene from top to down along the *z* direction as an example of obtaining permeation barrier using AIMD; the red points indicate the maximum and minimum energy points used to calculate proton permeation barriers. The brown and blue spheres represent carbon atoms (C) and the proton (H), respectively. (b) Distribution of calculated proton permeation energies of the studied materials from AIMD, highlighting the values of the benchmark graphene and h-BN. (c) Relationship between the calculated proton permeation barrier, the projected in-plane initial proton-surface distance (*d_xy*), pore size, number of 2D atomic layers, and type of nearest atom to proton in 2D materials. Pore size represents the average pore area of the individual pores in each of the 2D materials. The percentages (%) represent the proportion of specific categories within the corresponding data set. These samples account for the results obtained from AIMD within the 0−15 eV range across all 488 materials. The color of element types of nearest atom to proton in 2D materials are the same as in (d). (d) Proton permeation barriers in the range 0–1.4 eV extracted from (c), marked with the dashed red line in (c). The legends of pore size and 2D layers are the same as in (c).

The calculated permeation barriers serve as the target data set for ML models, while 60 fundamental features were collected as the ML input data set, as detailed in Table S1 in the Supporting Information. These features include structural and electronic properties, such as the projected in-plane initial proton−surface distance (i.e., the distance between the proton and the material’s atom closest to the proton in the *xy*-plane, *d_xy*), the average pore size of the 2D layer (*Pa*), the largest pore size in the 2D plane (*Pm*), the minimum interlayer spacing (*Lm*), and the average interlayer distance (*AvgL_d*), the electronegativity (*e_en*) and the electron affinity (*ea*) and a few energy-related features.

The distribution of proton permeation barriers across the 488 evaluated materials is illustrated in Figure 2b. Most of the studied materials exhibit permeation barriers in the range of 0 to 2 eV, indicating that many 2D nonmetallic materials offer relatively low resistance to proton transport. A concentration of materials at 15 eV represents those classified by the model as completely proton impermeable and then assigned this cutoff value. The relationship between proton permeation barriers and several essential features of the 2D-materials, including the projected in-plane initial proton−surface distance (see Figure S2 in the Supporting Information), local environment, average pore size (Figure S3), and layer thickness of 2D-materials can be inferred from the results shown in Figure 2c,d, where the complete data set obtained from AIMD calculations is shown, highlighting part of the data with permeation barriers below 1.4 eV. The permeation barrier and the projected in-plane initial proton−surface distance (*d_xy*) follow an inverse trend, resembling an exponential decay. As the proton in-plane distance to the closest atom of the 2D layer increases, the permeation barrier significantly decreases, following a rapid drop for distances below 1.25 Å. This suggests that at larger distances, the interactions weaken, resulting in lower barriers for proton permeation. However, for distances smaller than 1.25 Å, there is noticeable sparsity in barrier heights, indicating that additional factors, such as pore size, layer thickness and local atomic arrangement significantly impact proton transport in this regime. Both the number of atomic layers and pore size play critical roles in determining the barrier, with thicker structures and smaller pores presenting greater resistance to proton transport, as generally expected. Even when the proton distance to the surface atoms in the *xy* plane is moderate, around 1.25 Å, smaller pore sizes (e.g., circles and plus signs in Figure 2c,d) tend to be associated with higher permeation barriers. This implies that limited passing space with smaller pores restricts proton movement, leading to greater resistance. When comparing pore sizes and atomic layer numbers from the data set, it is observed that the proportion of materials with a pore area smaller than 1 Å^2^ significantly decreases in the low-barrier subset with energies below 1.4 eV. Similarly, the proportion of materials with more than 4 atomic layers decreases markedly.

The elements in the color legend in Figure 2d correspond to the closest atoms to the proton within the 2D structure, providing insights into the local environment during proton permeation. Based on the complete database shown in the subplot (Figure 2c), oxygen (O) and boron (B) represent a significant proportion of the elements in nonmetallic 2D materials. Most of the data points corresponding to lower permeation barriers, typically below 0.4 eV, are predominantly Sb (light pink), As (blue), B (light blue), and P (light brown). The electron affinity order of Cl > *F* > Br > I > S > Se > Te > O > Si > C > Ge > Sb > As > H > *P* > B > N^39^ suggests that elements with low electron affinities contribute to lower barriers, due to weaker proton binding or interactions. It is also observed that elements such as Se, Te, Si, and Ge are associated with relatively low permeation barriers, suggesting that a moderate electron affinity can provide a balanced interaction with the proton. However, elements like Cl and F with very high electron affinity could present challenges for proton permeation since it is more difficult for the proton to break the strong binding forces or attractive interactions.

### Machine Learning Performance

Our ML model establishes a predictive relationship between the fundamental physical features and proton permeation behavior, including basic geometric, electronic, and energy characteristics. After performing Pearson correlation analysis and Sure Independence Screening and Sparsifying Operator (SISSO) operation, 43 features from the original feature set were selected for ML modeling. DNN, GP, and RF were chosen to cover a range of learning paradigms and complexity, and each model was evaluated for predictive accuracy and generalization capability on both training and test data sets (see Figure 3a–c). The comparison shows that the RF model achieved the highest coefficient of determination value (*R* ^2^, eq 5) on the test set, explaining 90% of the variance in the proton permeation data. Additionally, the RF model yielded a mean absolute error (MAE) of 0.07, indicating strong predictive capability and high accuracy in predicting the barrier values. A unique observation for the GP model is the pronounced overfitting with imperfect generalization due to the relatively small training set of only 488 data points or noise in the data.

**Figure 3:**
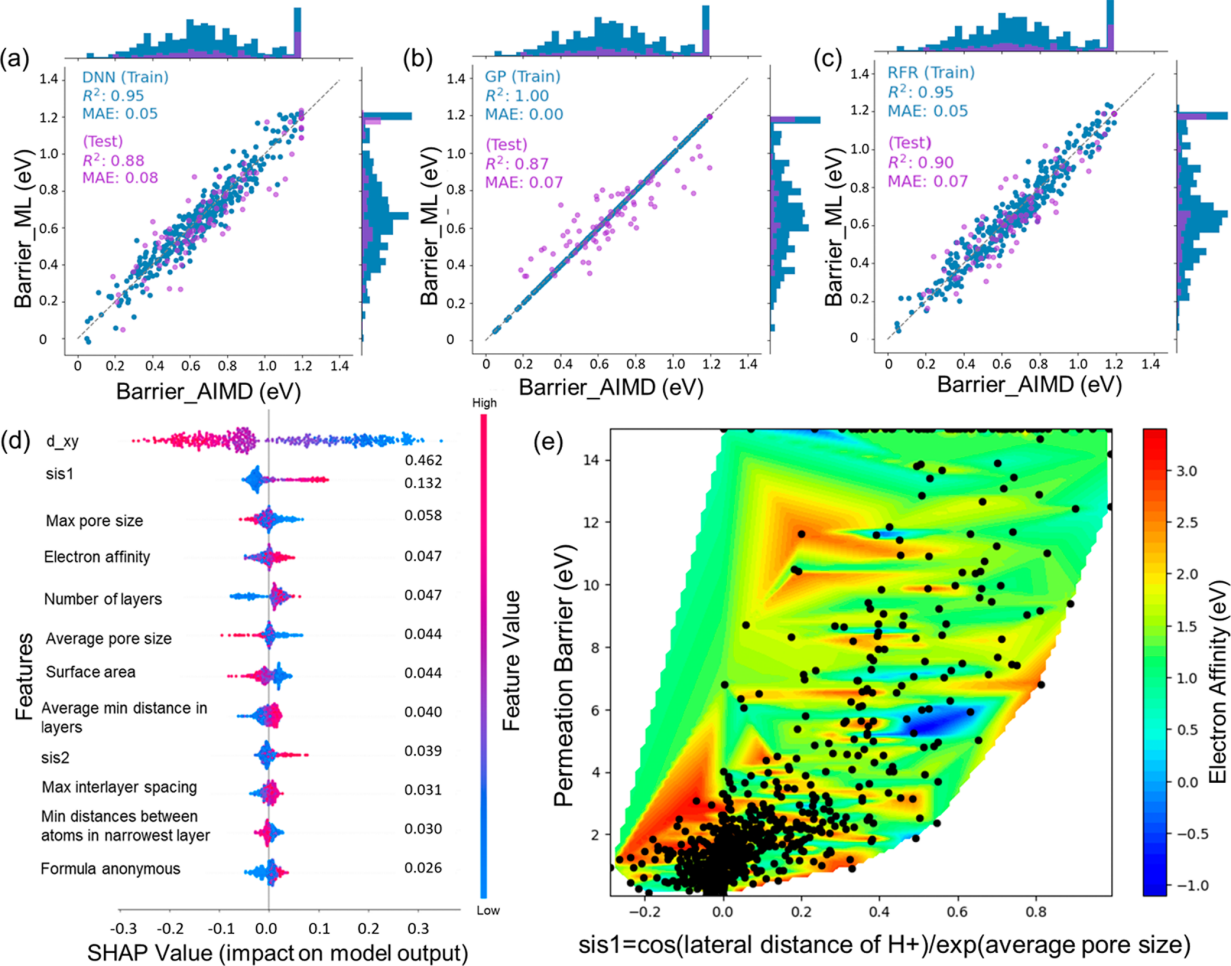
Comparison of the proton permeation barriers calculated using AIMD with predicted barriers from the DNN (a), GP (b), RF (c) models on the training and testing sets. The original barrier values were transformed using the Yeo-Johnson transformation^40^ for data normalization. The histogram shows the normalized barrier value distribution. (d) SHAP value analysis showing feature impact on RF model output. The SHAP value quantifies the feature’s impact on the model’s prediction of the proton permeation barrier. Positive SHAP values indicate that a feature increases the predicted value, whereas negative SHAP values indicate a decrease in the predicted value. The color of each point represents the magnitude of the feature value of each sample, blue indicates low feature values, red indicates high feature values. The ranking of features along *y*-axis indicates their importance on model predictions, with the numbers on the right representing their importance scores derived from RF feature importance calculation. (e) The relationship between the permeation barrier and SISSO generated highly related descriptors (sis1) and electron affinity. The heatmap was generated by linearly interpolating the electron affinity value, which allows us to connect the scattered data points smoothly and capture the overall trend and variation across the range. The black dots represent the actual calculated data points from the simulations.

The prediction performance of the GP and DNN models on the test data set present very similar error values compared with RF, which means these models can capture the underlying relationships in the proton permeation with a comparable level of accuracy, not dramatically outperforming the others in terms of error reduction. This similarity in error metrics suggests that the data’s underlying patterns are being effectively modeled regardless of the approach used. The histogram in Figure S4a illustrates the distribution of prediction errors for the Random Forest Regressor (RFR) model. The distribution appears roughly centered around 0, indicating that the model predicts values close to the simulation values with good overall accuracy and no significant bias toward overprediction or underprediction. Figure S4b presents the learning curve of the RFR. The training loss and cross-validation decrease rapidly as the training set size increases, eventually stabilizing at a relatively low value. This behavior indicates that the model fits the training data well while steadily improving its generalization to unseen data.

Table S2 summarizes and compares results from this work with other available ML studies focused on predicting the chemical properties of 2D materials. The table allows comparing the performance and generalization capabilities of the ML models, highlighting the current state of the art. Note that in a few cases, ML models do not perfectly predict permeation barriers due to the complexity of the chemisorption and the inherent intricacies of the materials. For example, as shown in Figure S1b, the multilayer structure Ge_3_N_4_ consistently exhibits a higher permeation barrier in the ML model than the results obtained from the simulations. This difference arises because features such as the number of layers, the interlayer spacing, and the pore size from a top-view perspective provide an approximate description of the material for the ML model. When the proton permeates through several layers of Ge_3_N_4_, the complex layer-by-layer interactions hinder the model’s ability to accurately capture the true barrier.

To better understand the contributions of each feature to the ML model’s predictions, the Shapley Additive Explanations (SHAP) plots of RF model in Figure 3d and SHAP plots of DNN and GPR model Figure S5 in the Supporting Information illustrate the impact of various features on the prediction of proton permeation barriers, facilitating the interpretation of the ML decision-making process. Figure 3d shows the importance ranking of features from the RF model and their corresponding importance scores. These scores are derived from the contribution of each feature in reducing the impurity of nodes across all trees in the forest. Finally, 12 features were identified with an importance score of more than 0.01.

It is observed that the initial in-plane distance between the proton and the atom closest to the proton from the top view (*d_xy*) is the most important feature for predicting the permeation barrier, with a score of approximately 0.46 (see Figure 3d). The scatter points show that large values of *d_xy* (in red) are associated with strong negative SHAP values, while small values of *d_xy* (in blue) are linked to large positive SHAP values. This indicates that extreme values of *d_xy* significantly impact the model’s predictions, and larger values of *d_xy* generally result in lower predicted permeation barriers, whereas smaller values lead to higher barriers. The physical interpretation is that increasing the distance from the pore center to the pore edge facilitates proton permeation, which also agrees with recent work^41^ showing that ripples enhance proton permeation as the rings are elongated. The second important feature is sis1 (defined in eq 1) related to the ratio between the in-plane distance between the proton and the material’s closest atom and the average pore size. High values of sis1 generally have a significant positive impact on the model’s prediction. In addition to the maximum pore size and average pore size, the interlayer structure also plays a crucial role in the behavior of some 2D materials. For instance, when the average minimum distance between neighboring atoms within the narrowest layer (*dm*) is small, atoms in this layer tend to cluster more tightly leaving larger interstitial voids between neighboring atoms. Additionally, a smaller number of atomic layers (*L*) or a larger maximum interlayer spacing (*Lm*) indicates a looser stacking, which results in smaller overall permeation barriers. Notice that the electron affinity (*ea*) significantly impacts the strength of bonding to hydrogen and, consequently, affects the model’s predictions. A higher electron affinity usually enhances proton-surface interactions, which can hinder proton transport. The SHAP analysis for the DNN and GP models is also presented in Figure S5 in the Supporting Information, revealing that the feature importance varies between these ML models. All models consistently indicate that structural descriptors primarily dictate the geometric accessibility for proton transport, while electronic descriptors, such as electron affinity and local charge distribution, modulate the strength of proton−surface interactions. This complementary interplay between geometry and electronic structure reinforces the physical interpretability of the ML framework. Notably, although these features align well with physical intuition, the model autonomously identified them from an initial set of 60 descriptors, without human bias or prior assumptions.

The correlations between several top-ranking features and the permeation barrier were demonstrated in Figure 2c. Furthermore, Figure 3e highlights the influence of two additional key features: a combined geometric descriptor generated from SISSO (sis1, eq 1), and electron affinity (*ea*). From the heatmap, electron affinity exhibits significantly higher values in the low permeation barrier region. This suggests that materials possessing both enhanced electron-accepting capabilities and favorable geometric configurations of large pore size (small sis1 values) exhibit low permeation barriers, which is beneficial for charge transfer and proton injection. Conversely, in the high permeation barrier domain, the permeation barrier increases as electron affinity decreases, even with a small sis1 value in restricting electron capture.

The decision-making process of the RFR model is visualized in Figure S6a in the Supporting Information, providing a detailed view of the feature thresholds used to predict the target variable and offering insights into the relationships captured by the model. The root node splits on *d_xy* ≤ 1.38 Å, signifying that this feature has the most substantial influence on the predictions, forming the primary division of the data set. Subsequent nodes further guide predictions through features such as pore size (*Pm* and *Pa*), surface area (*A*), number of atomic layers (*L*), sis2 (eq 2), and electron affinity (*ea*), highlighting their relative significance in the model. Features related to intralayer atomic distance, such as *dm* and *dma*, are typically utilized in lower levels of the tree, indicating their role in capturing finer details in making decisions. Pathways resulting in predicted normalized values below 0.65 eV (light color nodes in the last level of the tree) primarily branch through the condition *d_xy* ≥ 1.38 Å, which directs samples to the right-hand side of the tree. For *d_xy* ≥ 1.75 Å, the predicted normalized barriers are generally below 0.65 eV, with only a few exceptions where *dm* ≤ 2.73 with *dma* ≤ 3.15 or *Lm* ≤ 1.66. The pathway with the lowest barrier (white node) follows the conditions *d_xy* ≥ 1.75, sis2 ≤ 0, *dm* ≤ 3.08, and 2.29 ≥ *dma* ≥ 1.61. Moreover, Figure S6b represents a hierarchical decision-making process of the well-trained Random Forest Classifier (RFC, accuracy = 99%), which uses feature thresholds to classify materials as proton-permeable or impermeable. The root node splits the data on the condition sis2 ≤ 0.0, identifying it as the most influential feature and dividing the data set into a larger branch with 356 samples (sis2 ≤ 0.0) and a smaller branch with 34 samples (sis2 ≥ 0.0). Subsequent splits use features such as *A*, *Pm*, *d_xy*, and *dma* to refine predictions, with each level capturing more detailed patterns in the data. The darkest orange node appears in the subtree under specific conditions (*d_xy* ≤ 0.91 or *A* ≥ 123.32), suggesting these thresholds are highly predictive of impermeability. The pathway combines larger *d_xy*, large pore sizes, lower electron affinity, and larger *dma* to achieve a predicted permeation barrier below 1.4 eV. These criteria provide a clear guide for membrane design by identifying the most critical parameters and their specific ranges that result in low permeation barriers.

### AIMD for H^+^/H_2_ Selectivity

Using the trained RF model, proton permeation barriers for 378 unknown 2D materials were predicted and combined with AIMD data, identifying 388 materials with barriers below 1.4 eV. These qualified materials were subsequently evaluated using AIMD to examine the permeability of H_2_ and the selectivity between H_2_ and proton (H^+^) transport. While most materials allow H_2_ to permeate directly, Figure 4 highlights two representative behaviors observed in AIMD simulations where H_2_ does not freely permeate for the promising candidates: scenario 1 with H_2_ dissociation (see Figure 4a,b): when a proton passes through the 2D layer at a given velocity, it interacts strongly with neighboring atoms forming strong chemical bonds. These bonds dissipate part of the proton’s kinetic energy and elongate the H–H bond to weaken the H–H attraction, ultimately leading to the dissociation of H_2_; and scenario 2 where the H–H bond stretches (see Figure 4c). In some cases, as H_2_ passes through the 2D layer, the bond length temporarily extends beyond 1 Å. However, the elongation is insufficient to overcome the H–H attractive forces, and the protons revert to their original H_2_ molecules. In scenario 2, where H_2_ bonds stretch but do not dissociate, the recorded permeation barriers are exceptionally high, exceeding 9 eV. These materials demonstrate strong resistance to H_2_ transport, making them particularly relevant for further investigation as selective proton conductors.

**Figure 4:**
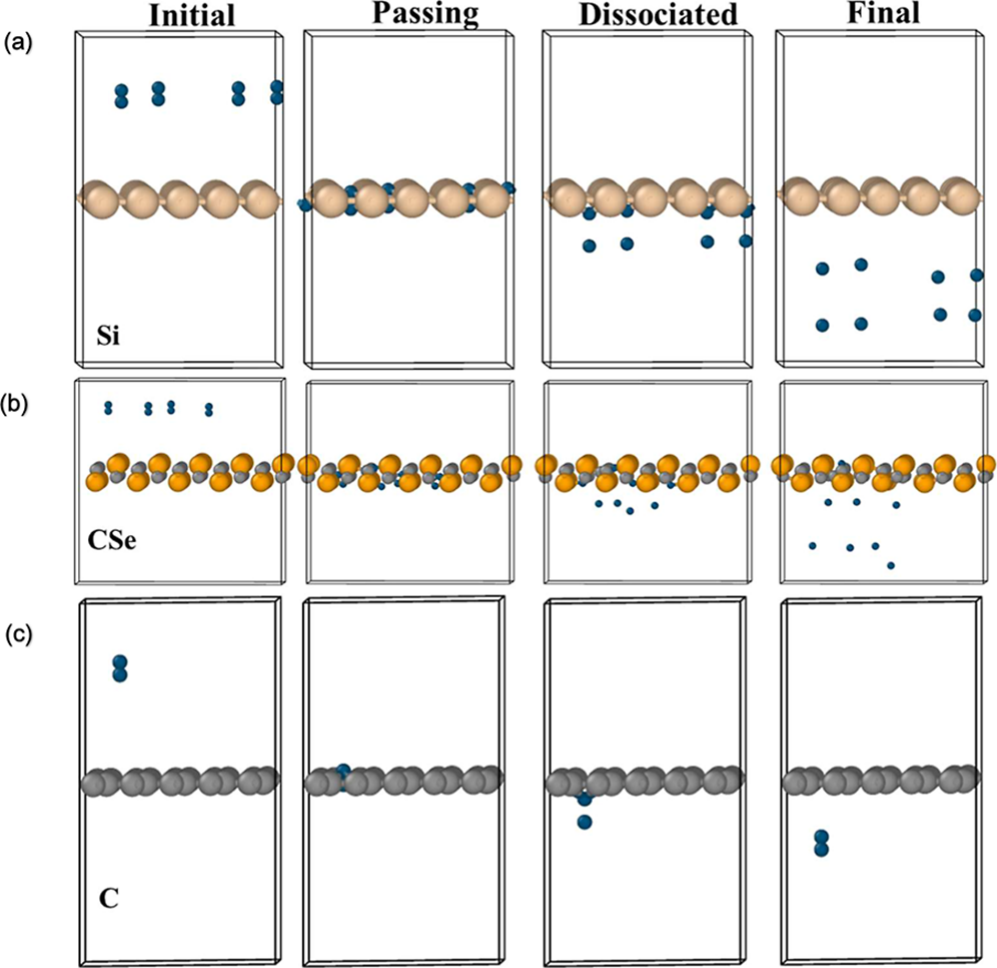
AIMD simulations of H_2_ permeation and bond dynamics through 2D layers. (a)-(b) Two examples showing the dissociation of H_2_ during permeation through 2D layers. Multiple H_2_ molecules were placed at different positions to verify and confirm the observed behaviors. (c) An example to show the H–H bond stretch and reformation of H_2_ during permeation through 2D layers.

For PEMs, the selectivity between H^+^ and H_2_ is critical, as high H^+^ permeability ensures efficient proton conduction, while low H_2_ permeability prevents fuel crossover. Excessive H_2_ permeation reduces system efficiency and poses potential safety risks by enabling unintended H_2_–O_2_ mixing, which could form combustible mixtures under certain conditions. Thus, materials with high selectivity are essential for optimal performance and safe operation in PEM-based systems. As shown in Figure 5, materials with a significant gap between H^+^ and H_2_ permeation barriers, particularly those represented by hollow symbols, are ideal for achieving high selectivity in PEMs. In some of these materials, H_2_ undergoes chemisorption and dissociation during permeation, revealing distinct properties that enable dual functionality. These materials show promise not only as proton exchange membranes but also as catalytic layers, leveraging their ability to dissociate H_2_ and suppress recombination for enhanced catalytic performance.

**Figure 5:**
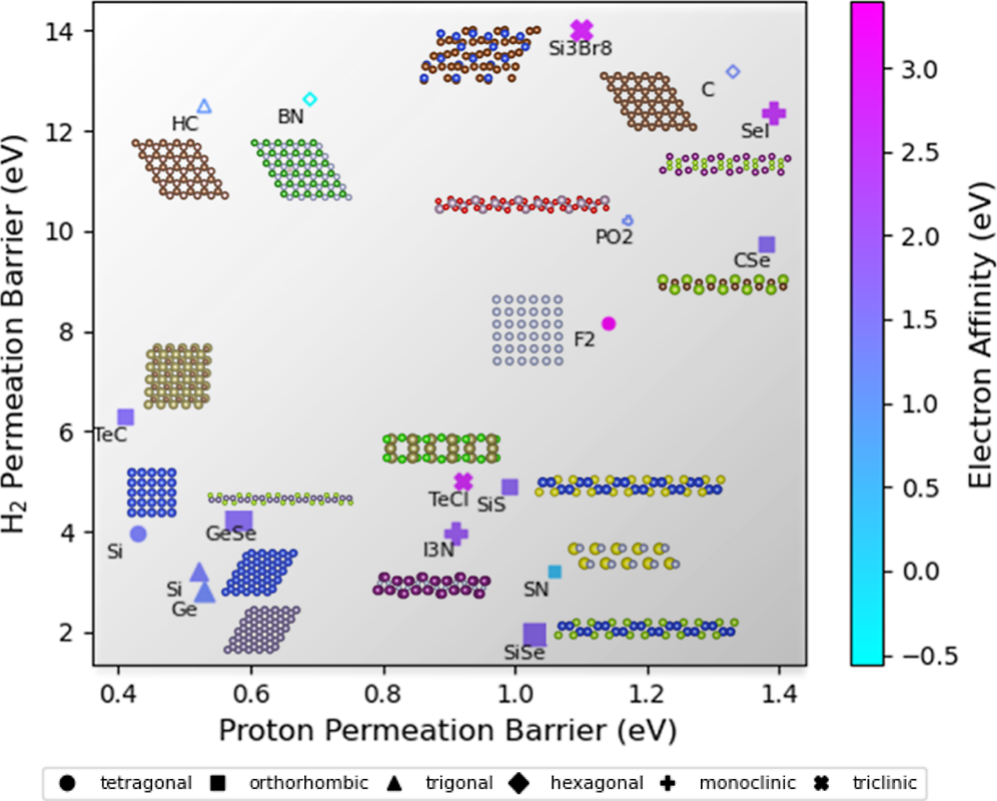
Relative selectivity between proton and H_2_ permeation across 2D materials. The symbols represent different crystal structures. The color scale corresponds to the electron affinity (in eV), with light blue indicating lower values and light purple indicating higher values. The size of each symbol reflects the relative value of the average minimum distances between atoms within each layer (*dma*), representing the intralayer atomic distance. Solid symbols indicate materials where H_2_ dissociates during permeation. Open symbols represent cases where H_2_ does not completely dissociate but exhibits high barriers (>9 eV).

To further examine how structural and electronic features influence the AIMD-observed H_2_ permeation behaviors, we analyzed the screened candidate materials by mapping their electron affinity, intralayer atomic distance, and crystal symmetry (see Figure 5). It is observed that materials capable of H_2_ dissociation generally display relatively higher electron affinity and moderately compact atomic arrangements. These features may stabilize adsorbed hydrogen atoms and favor a dissociation-mediated transport pathway. In contracts, materials that exhibit high H_2_ permeation barrier tend to have lower electron affinity and small intralayer atomic distance.

To evaluate promising materials based on H^+^/H_2_ selectivity, the compounds shown in Figure 5 are also listed in Table S3 of the Supporting Information, summarizing their key properties and prior research status. Notice that we did not exclude zero-band gap materials, since their electronic structure can be tuned (e.g., by strain, fields, or doping); instead, our screening focuses on intrinsic proton conductivity, with band gap optimization considered at later design stages. Some materials provided in Table S3 of the Supporting Information have been experimentally synthesized and applied in PEMs, such as silica nanosheets,^42^ graphene,^43–45^ h-BN^46–48^ and silicene.^49,50^ Additionally, Komma et al.^43^ demonstrated the successful integration of single-layer graphene into PEMs, confirming its strong hydrogen-gas blocking capability. However, under typical humidified operating conditions of PEM fuel cells, the hydrogen-blocking performance declines due to reversible interlayer changes caused by humidification and irreversible defect formation during operation. Several functionalized graphene-based materials have been specifically developed for PEM applications.^44,45^ For example, hydrogen plasma treatment provides an efficient approach for surface functionalization of single-layer graphene and enables wafer-scale fabrication.^51,52^ Two comprehensive reviews^53,54^ explore the structure, physical and chemical properties, and formation mechanisms of graphane (hydrogenated graphene), summarizing advancements in its synthesis and engineering methods and potential applications in hydrogen storage, electronics and other fields. Moreover, several review articles^46–48^ highlight h-BN as a promising material for membrane applications due to its stability, mechanical strength, high proton conductivity (∼100 mS·cm^−1^), and high separation factor (∼10) between protons and deuterons. Large-scale h-BN membranes have been successfully applied in fuel cells and batteries. Furthermore, monolayer germanene, a graphene-like germanium allotrope, has garnered significant attention due to its high carrier mobility and tunable properties.^55–58^ To the best of our knowledge, it has yet to be explored for PEM applications, even at the theoretical level.

Monolayer Group IV−VI monochalcogenides (MXs, M = Ge, Si, Sn; X = S, Se) have garnered significant attention for their promising thermoelectric and optical properties. GeSe has been experimentally synthesized^59^ and extensively studied using DFT,^60,61^ standing out as an appealing photovoltaic material due to its advantageous electronic and optical characteristics. However, its application in PEMs remains unexplored. Another 2D material SiS demonstrates exceptionally low thermal conductivity along in-plane directions; theoretical studies^62–64^ of SiS are abundant, but experimental synthesis remains elusive. Similarly, SiSe has been extensively studied theoretically^65–67^ for water splitting and nanodevices, with predicted high stability in aqueous environments and ambient electrochemical conditions, though experimental validation is lacking. Carbon-based monochalcogenides (CSe, TeC) remain largely unexplored despite their excellent predicted thermoelectric and solar energy conversion potential. In particular, 2D CSe represents a promising alternative to phosphorene for renewable energy applications.^68^

It should be noted that several materials shown in Figure 5 and Table S3 in the Supporting Information are relatively uncommon in 2D materials research. For example, nitrogen triiodide (NI_3_) is impractical due to its unusual negative dissociation energy (−290 kJ·mol^−1^) and extreme sensitivity to mechanical impact. To date, only two studies^69,70^ have addressed the stability of this hazardous 3D compound. Similarly, tellurium carbide (TeC) has received minimal attention, with only patents^71^ on TeC films. Other compounds such as TeCl, SN, Si_3_Br_8_, F_2_, SeI and PO_2_ are theoretically stable but present significant challenges for experimental realization. While their possible application in PEMs requires additional research, our computational screening highlights their promise for membrane technologies, warranting further investigation despite their current experimental inaccessibility.

Beyond synthesis challenges, the environmental stability of many computationally identified 2D materials under realistic PEM operating conditions (humid, oxidative environments) remains a critical concern. Even among experimentally demonstrated candidates, materials such as silicene and germanene are known to be extremely air-sensitive and would likely require protective strategies to maintain structural integrity in practical fuel cells. Similarly, other theoretically stable materials may face severe degradation under conditions typical of fuel-cell operation. Experimental evaluation of long-term stability will be essential for these computationally promising candidates.

## Conclusions

In this work, we applied a combined AIMD and machine learning approach to explore a wide range of nonmetallic 2D materials for PEMs in hydrogen fuel cells, focusing on how atomic-scale structural and electronic features influence proton conductivity and H^+^/H_2_ selectivity. Our workflow enabled the identification of both experimentally demonstrated and theoretically predicted 2D materials with high proton selectivity.

AIMD simulations on 488 structures generated a data set of permeation barriers, which was used to train three ML models, with Random Forest achieving the best performance (*R* ^2^ = 0.90, MAE = 0.07 eV). Applying this model to the remaining 378 materials identified a total of 388 candidates with barriers below 1.4 eV, surpassing graphene. Key features governing proton transport include proton-atom distance >1.38 Å, large pore size, low electron affinity, and wide intralayer atomic distance. Subsequent AIMD simulations assessing H^+^/H_2_ selectivity revealed distinct dissociation behaviors. This screening highlighted 18 promising PEM candidates: well-studied materials like graphene and h-BN (validating the approach), experimentally synthesized but unexplored compounds such as hydrogenated graphene, silicene, germanene, diamane, TeC, TeCl, GeSe, and CSe, and theoretically stable 2D materials (SN, SiSe, SiS, Si_3_Br_8_, F_2_, SeI, PO_2_) as longer-term targets for synthesis and evaluation.

Overall, these findings provide a roadmap for designing next-generation PEMs and guide further exploration of 2D materials for hydrogen-selective membranes. More broadly, the integration of AIMD with ML underscores the critical role of specific structural and electronic features in proton transport, accelerating the discovery of advanced PEMs with enhanced performance.

## Methods

### Workflow

AIMD and ML have been combined in this work to predict and analyze permeation barriers, enabling the systematic identification of promising 2D materials for PEMs, as shown in a schematic manner in Figure 1. The initial step involved collecting 866 structures from 2Dmatpedia^30^ online database, specifically focusing on identifying all nonmetallic materials. Then, features such as elemental compositions, pore sizes, layer thickness, electronic affinities, stacking types, and other relevant properties for these identified 2D materials were gathered to serve as input data sets for ML. Subsequently, AIMD simulations were conducted to determine proton permeability and permeation barriers of a subset of 488 materials, which served as the target data set for ML. Regarding the ML regression and classification algorithms, DNN, GP, and RF were employed to learn the proton permeation performance across the obtained data set. Regression models were first used to predict the proton permeation barrier, and classification models were subsequently employed for impermeable materials to determine whether a material is permeable to protons. Materials classified as impermeable were further assigned a permeation barrier of 15 eV. The performance of these models was evaluated based on the metrics of accuracy for classification tasks, mean-average error (MAE) and coefficient of determination (*R* ^2^) for regression tasks. By comparing the performance of each model and harnessing the strength of each approach, we ensured robust and accurate predictions of proton permeability properties, providing valuable insights into the proton permeation characteristics of the materials. The trained ML models were further utilized to predict the permeability and energy barriers for the remaining 378 2D materials. Materials predicted to have a proton permeation barrier lower than 1.4 eV (lower than the obtained theoretical value for graphene) were selected as promising candidates. In the final step, these promising materials were further evaluated using AIMD simulations to assess their H^+^/H_2_ selectivity. Specifically, H_2_ selectivity was determined by evaluating whether H_2_ molecules get entirely blocked or permeate the 2D layers with high permeation barrier (>10 eV). This screening process ultimately identifies potential 2D candidates for highly efficient PEMs.

### AIMD Simulations

To calculate the potential energy barrier for proton permeation across various nonmetallic 2D materials, AIMD simulations were conducted using the Vienna Ab-initio Simulation Package (VASP).^72–74^ Electron−ion interactions were described using the projector augmented wave (PAW) method,^75^ optimizing computational performance without sacrificing accuracy. Molecular dynamic simulations were employed with a time step of 0.2 fs to accurately capture the dynamic behavior of protons within the material framework. The plane-wave cutoff energy was set to 400 eV. A dispersion correction method was included to account for van der Waals interactions. Gaussian smearing with a width of 0.05 eV was used to improve the electronic state occupation’s numerical stability.^76^ A maximum of 200 ionic steps were set to properly explore the potential energy surface and the proton permeation pathways. The simulations operated in the microcanonical ensemble (NVE), ensuring the conservation of total energy within the system. The initial temperature was set to 300 K. The total number of electrons was explicitly specified to ensure hydrogen acts as a proton in the system throughout the simulation.

Permeation was assessed in vacuum (i.e., no aqueous/solvent media). The *pymatgen* package^77^ was utilized to generate a diverse set of structures with one proton placed 10 Å above the 2D layers, and an initial velocity of −0.5 Å/fs along the *z*-direction (perpendicular to the 2D-material) was assigned to push it toward the basal plane of the material. Three possible proton placement configurations were considered based on maximizing the distance from surrounding surface atoms in the *xy*-plane by using the hollow-site detection function in *pymatgen* (one example is shown in Figure S2). This strategy ensured that the proton was positioned directly above the center of the pore, allowing searching for the minimum permeation energy during proton penetration, thereby optimizing the efficiency and computational resources. After this initialization with velocity, the proton’s motion was fully governed by first-principles molecular dynamics, without any constraints on its subsequent trajectory. Therefore, if the repulsive interactions from the 2D material or its local atomic distribution favor a curved or off-center pathway, the proton is free to follow such a trajectory during the AIMD evolution. For the supercell size, the simulation box of the unit system was enlarged to contain approximately 200 atoms in total, striking a balance between computational manageability, practicality, and representativeness.

### Key Features and Machine Learning Models

The fundamental structural, electronic, and energetic properties were considered in the ML model, aiming at utilizing easily obtainable features to capture the key factors influencing proton permeation effectively. A total of 60 features were initially chosen, with detailed abbreviations, descriptions, and sources provided in Table S1. The code details for feature collections and ML models can be found in the Supporting Information.

Among the selected features, the projected in-plane initial proton position from the surrounding surface atoms in the *xy*-plane (Figure S2) providing the lowest energy barrier in the AIMD calculations was named as feature *d_xy*. Additionally, the pore size in the 2D materials required special image processing. The process involved initially transforming the image to a grayscale format, then the Otsu thresholding method was applied to convert the image into a binary format. The pores were further identified and quantified through connectivity labeling and analysis of region properties, which assigns a unique label to each group of connected white pixels, so it can be recognized as ″*these pixels belong to the same pore region*.″ Once the pore regions were identified, their area and shape were calculated using region property analysis. Specifically, the “*regionprops*” function in python was used to obtain the areas of the pore regions, excluding regions smaller than 50 pixels to omit noise. Based on this method, we identified all individual pores in the 2D material. The pore average feature (*Pa*) is then obtained by averaging the areas of all pores in the given material, while *Pm* corresponds to the largest pore area among them. In the case 2D materials have only one type of pores, *Pa* = *Pm*, as presented in Figure S3.

The Sure Independence Screening and Sparsifying Operator (SISSO)^78^ works by initially screening the features using a measure of independence, such as mutual information, to identify the most meaningful ones, and subsequently applying a sparsifying operator that enforces sparsity and further reduces the feature space. Based on this technique, two top-ranked descriptors (sis1, sis2) were calculated as

sis1=cos(d_xy)/exp(Pa) 1

2 $$sis2=\frac{nele}{atom\_n\_lay\times \left(sg+atom\_n\_lay\right)}$$

where *d_xy* is the projected in-plane initial proton−surface distance, *Pa* the average pore size of the 2D layer, *nele* the number of element types in the 2D material, *atom_n_lay* the total number of atoms in the narrowest layer and *sg* the space group of the lattice in the 2D material. Notice that all features used to define sis1 and sis2 are under the Geometrical-type category (see Table S1 in the Supporting Information).

Pearson correlation analysis was employed to eliminate highly correlated features and ensure that the ML model was trained in unique and independent features, ultimately improving model performance and reducing overfitting risks. Features with an absolute Pearson correlation coefficient (see Figure S7) greater than 80% were identified as redundant and removed based on their linear correlation with the permeation barrier. We generated two new features (see eqs 1 and 2) by combining the descriptors identified through SISSO to improve the ML model’s performance and effectively handle data sets with high-dimensional features.

All ML algorithms were conducted by the open-source code Scikit-learn package in the Python (version 3.10.5) environment. DNN, GP and RF were selected as they represent distinct categories of ML models: decision tree-based, probabilistic, and neural network-based, respectively. This diversity ensures a comprehensive exploration of proton permeation behavior, allowing us to leverage the unique strengths of each model type and tailor to specific data characteristics and problem types. Before model training, all features were normalized using the StandardScaler function in scikit-learn, which standardizes each descriptor to zero mean and unit variance. This preprocessing was applied to the DNN and GP models to ensure consistent feature scaling. In contrast, the RF model was trained on raw data as RF models are inherently insensitive to feature scaling: their split rules rely on thresholding individual features rather than gradient-based optimization. Feature scaling does not alter the decision tree structure, as their splits and weights are scale independent.

Notably, for the RFR model, features with importance scores greater than 0.01 were selected for a second round of training and prediction. This iterative process aimed at refining the model by focusing on the most impactful features, ultimately enhancing prediction accuracy and reducing model complexity. The calculated energy barriers were randomly split as training and testing sets according to an 80:20 ratio. Random search, where hyperparameters are randomly sampled from a defined range or distribution, was employed for using cross-validation to estimate the performance of each hyperparameter combination, the parameters of best model are summarized in Table S4. To evaluate the prediction performance of each model, accuracy for classification, mean-average error (MAE), and coefficient of determination (*R* ^2^) for regression, have been calculated for the training and testing sets. The formula for each one of the indicators is as follows

3 $$accuracy=\frac{TP+TN}{TP+TN+FP+FN}$$

4 $$MAE=\frac{1}{m}\sum_{i=1}^{m}\left|y_{i}-y_{pi}\right|$$

5 $$R^{2}=1-\frac{\sum_{i=1}^{m}{\left(y_{i}-y_{pi}\right)}^{2}}{\sum_{i=1}^{m}{\left(y_{i}-{\mathrm{y̅}}_{i}\right)}^{2}}$$

where TP is true positive, TN is true negative, FP is false positive, FN is false negative, *y*_*i*_ is the permeation barrier value from AIMD calculations randomly selected from data set, *y*_*pi*_ is the predicted value of the corresponding regression model, ${\mathrm{y̅}}_{i}$ is the average value of *y*_*ti*_, *m* is the number of samples in the data set. Generally, an ideal model should have *R* ^2^ value close to 1, and a small MAE close to 0.

## Supplementary Material



## Data Availability

Data is available on request.

## Notes

Summary of 60 initial features used for machine learning models, machine learning studies for proton permeation prediction on 2D-materials, key properties and descriptions of screened materials for PEMs, the hyper-parameters of DNN, RF, and GP algorithms for classification (C) and regression (R) tasks, the relative potential energy profile of proton permeation for three different materials, representative initial proton placement configurations above the 2D layers from top view (xy plane), shown as an example for B_2_O_3_, top view of the proton placement configurations above the 2D materials for three selected materials with different geometry and composition, the histogram of prediction errors for the Random Forest Regressor model, Shapley Additive Explanations (SHAP) value analysis showing feature impact on DNN and GPR model output, visualization of the trained Random Forest Regressor (RFR) to highlight the key decision pathways of the model, Pearson correlation coefficient map for selected features (PDF)

## References

[1] Jiao K., Xuan J., Du Q., Bao Z., Xie B., Wang B., Zhao Y., Fan L., Wang H., Hou Z., Huo S., Brandon N. P., Yin Y., Guiver M. D. (2021). Designing
the next Generation of Proton-Exchange Membrane Fuel Cells. Nature.

[2] Haider R., Wen Y., Ma Z.-F., Wilkinson D. P., Zhang L., Yuan X., Song S., Zhang J. (2021). High Temperature
Proton Exchange
Membrane Fuel Cells: Progress in Advanced Materials and Key Technologies. Chem. Soc. Rev..

[3] Lee K.-S., Spendelow J. S., Choe Y.-K., Fujimoto C., Kim Y. S. (2016). An Operationally
Flexible Fuel Cell Based on Quaternary Ammonium-Biphosphate Ion Pairs. Nat. Energy.

[4] Lei Y., Zhang T., Lin Y.-C., Granzier-Nakajima T., Bepete G., Kowalczyk D. A., Lin Z., Zhou D., Schranghamer T. F., Dodda A., Sebastian A., Chen Y., Liu Y., Pourtois G., Kempa T. J., Schuler B., Edmonds M. T., Quek S. Y., Wurstbauer U., Wu S. M., Glavin N. R., Das S., Dash S. P., Redwing J. M., Robinson J. A., Terrones M. (2022). Graphene and Beyond:
Recent Advances in Two-Dimensional Materials Synthesis, Properties,
and Devices. ACS Nanosci. Au.

[5] Moghadam P. Z., Chung Y. G., Snurr R. Q. (2024). Progress
toward the Computational
Discovery of New Metal−Organic Framework Adsorbents for Energy
Applications. Nat. Energy.

[6] Xu J., Jiang H., Shen Y., Li X.-Z., Wang E. G., Meng S. (2019). Transparent Proton
Transport through a Two-Dimensional Nanomesh Material. Nat. Commun..

[7] Nauman
Javed R. M., Al-Othman A., Tawalbeh M., Olabi A. G. (2022). Recent
Developments in Graphene and Graphene Oxide Materials for Polymer
Electrolyte Membrane Fuel Cells Applications. Renewable Sustainable Energy Rev..

[8] DuChanois R. M., Porter C. J., Violet C., Verduzco R., Elimelech M. (2021). Membrane Materials
for Selective Ion Separations at the Water−Energy Nexus. Adv. Mater..

[9] Seel M., Pandey R. (2016). Proton and Hydrogen
Transport through Two-Dimensional
Monolayers. 2D Mater..

[10] Wu Z. F., Sun P. Z., Wahab O. J., Tan Y. T., Barry D., Periyanagounder D., Pillai P. B., Dai Q., Xiong W. Q., Vega L. F., Lulla K., Yuan S. J., Nair R. R., Daviddi E., Unwin P. R., Geim A. K., Lozada-Hidalgo M. (2023). Proton and
Molecular Permeation through the Basal Plane of Monolayer Graphene
Oxide. Nat. Commun..

[11] Wang N., Yuan B., Tang C., Du L., Zhu R., Aoki Y., Wang W., Xing L., Ye S. (2022). Machine-Learning-Accelerated
Development of Efficient Mixed Protonic−Electronic Conducting
Oxides as the Air Electrodes for Protonic Ceramic Cells. Adv. Mater..

[12] Hammes-Schiffer S., Galli G. (2021). Integration of Theory
and Experiment in the Modelling of Heterogeneous
Electrocatalysis. Nat. Energy.

[13] Lu S., Zhou Q., Guo Y., Zhang Y., Wu Y., Wang J. (2020). Coupling a Crystal
Graph Multilayer Descriptor to Active Learning
for Rapid Discovery of 2D Ferromagnetic Semiconductors/Half-Metals/Metals. Adv. Mater..

[14] Li W., Liu W., Jia W., Zhang J., Zhang Q., Zhang Z., Zhang J., Li Y., Liu Y., Wang H., Xiang Y., Lu S. (2024). Dual-Proton
Conductor for Fuel Cells
with Flexible Operational Temperature. Adv.
Mater..

[15] Yang L., Cong E., Hao Z., Bo C., Cui Y., Xu S., Wu R., Li Q., Zhang X., Zhang S., Yang L. (2022). Selective Penetration
Mechanism of Hydrogen Isotope through Graphene
Membrane. Carbon.

[16] Kroes J. M. H., Fasolino A., Katsnelson M. I. (2017). Density
Functional Based Simulations
of Proton Permeation of Graphene and Hexagonal Boron Nitride. Phys. Chem. Chem. Phys..

[17] Hu S., Lozada-Hidalgo M., Wang F. C., Mishchenko A., Schedin F., Nair R. R., Hill E. W., Boukhvalov D. W., Katsnelson M. I., Dryfe R. A. W., Grigorieva I. V., Wu H. A., Geim A. K. (2014). Proton Transport through One-Atom-Thick
Crystals. Nature.

[18] Miao M., Nardelli M. B., Wang Q., Liu Y. (2013). First Principles
Study
of the Permeability of Graphene to Hydrogen Atoms. Phys. Chem. Chem. Phys..

[19] Burke K. (2012). Perspective
on Density Functional Theory. J. Chem. Phys..

[20] Feng Y., Chen J., Fang W., Wang E.-G., Michaelides A., Li X.-Z. (2017). Hydrogenation Facilitates Proton Transfer through Two-Dimensional
Honeycomb Crystals. J. Phys. Chem. Lett..

[21] Tuckerman M. E. (2002). Ab Initio
Molecular Dynamics: Basic Concepts, Current Trends and Novel Applications. J. Phys.: Condens. Matter.

[22] Devanathan R., Idupulapati N., Baer M. D., Mundy C. J., Dupuis M. (2013). Ab Initio
Molecular Dynamics Simulation of Proton Hopping in a Model Polymer
Membrane. J. Phys. Chem. B.

[23] Xu F., Wang Y., Lian C., Xu Z. (2022). Fast Proton-Selective
Transport through Covalent Organic Frameworks in Aqueous Phase. J. Membr. Sci..

[24] Shi L., Xu A., Pan D., Zhao T. (2019). Aqueous Proton-Selective Conduction
across Two-Dimensional Graphyne. Nat. Commun..

[25] Chai G.-L., Shevlin S. A., Guo Z. (2017). Nitrogen-Mediated
Graphene Oxide
Enables Highly Efficient Proton Transfer. Sci.
Rep..

[26] Xin H. (2022). Catalyst Design
with Machine Learning. Nat. Energy.

[27] Priya P., Nguyen T. C., Saxena A., Aluru N. R. (2022). Machine Learning
Assisted Screening of Two-Dimensional Materials for Water Desalination. ACS Nano.

[28] Dronadula M. T., Aluru N. R. (2025). Catalysis-Mediated, Ion-Size Modulation-Driven Separation
of Transition Metal Complexes. ACS Nano.

[29] Haastrup S., Strange M., Pandey M., Deilmann T., Schmidt P. S., Hinsche N. F., Gjerding M. N., Torelli D., Larsen P. M., Riis-Jensen A. C. (2018). The Computational 2D Materials Database: High-Throughput
Modeling and Discovery of Atomically Thin Crystals. 2D Mater..

[30] Zhou J., Shen L., Costa M. D., Persson K. A., Ong S. P., Huck P., Lu Y., Ma X., Chen Y., Tang H. (2019). 2DMatPedia, an Open
Computational Database of Two-Dimensional
Materials from Top-down and Bottom-up Approaches. Sci. Data.

[31] Mounet N., Gibertini M., Schwaller P., Campi D., Merkys A., Marrazzo A., Sohier T., Castelli I. E., Cepellotti A., Pizzi G. (2018). Two-Dimensional Materials from High-Throughput Computational
Exfoliation of Experimentally Known Compounds. Nat. Nanotechnol..

[32] Campi D., Mounet N., Gibertini M., Pizzi G., Marzari N. (2022). Novel Materials
in the Materials Cloud 2D Database. Mater. Cloud
Arch.

[33] Zhai S., Xie H., Cui P., Guan D., Wang J., Zhao S., Chen B., Song Y., Shao Z., Ni M. (2022). A Combined
Ionic Lewis Acid Descriptor and Machine-Learning Approach to Prediction
of Efficient Oxygen Reduction Electrodes for Ceramic Fuel Cells. Nat. Energy.

[34] Zhang Q., Yuan Y., Zhang J., Fang P., Pan J., Zhang H., Zhou T., Yu Q., Zou X., Sun Z., Yan F. (2024). Machine Learning-Aided Design of Highly Conductive
Anion Exchange Membranes for Fuel Cells and Water Electrolyzers. Adv. Mater..

[35] Daglar H., Keskin S. (2022). Combining Machine Learning
and Molecular Simulations
to Unlock Gas Separation Potentials of MOF Membranes and MOF/Polymer
MMMs. ACS Appl. Mater. Interfaces.

[36] Bai X., Shi Z., Xia H., Li S., Liu Z., Liang H., Liu Z., Wang B., Qiao Z. (2022). Machine-Learning-Assisted High-Throughput
Computational Screening of Metal−Organic Framework Membranes
for Hydrogen Separation. Chem. Eng. J..

[37] Barnett J. W., Bilchak C. R., Wang Y., Benicewicz B. C., Murdock L. A., Bereau T., Kumar S. K. (2020). Designing Exceptional
Gas-Separation Polymer Membranes Using Machine Learning. Sci. Adv..

[38] Bento A. P., Hersey A., Félix E., Landrum G., Gaulton A., Atkinson F., Bellis L. J., De Veij M., Leach A. R. (2020). An Open
Source Chemical Structure Curation Pipeline Using RDKit. J. Cheminf..

[39] lmmentel/Mendeleev .A Python Resource for Properties of Chemical Elements; Ions and Isotopes, 2014 https://github.com/lmmentel/mendeleev (accessed May 20, 2024).

[40] Box G. E.
P., Cox D. R. (1964). An Analysis
of Transformations. J. R. Stat. Soc. Ser. B
Methodol..

[41] Wahab O. J., Daviddi E., Xin B., Sun P. Z., Griffin E., Colburn A. W., Barry D., Yagmurcukardes M., Peeters F. M., Geim A. K., Lozada-Hidalgo M., Unwin P. R. (2023). Proton Transport through Nanoscale Corrugations in
Two-Dimensional Crystals. Nature.

[42] Guo Z., Chen J., Byun J. J., Cai R., Perez-Page M., Sahoo M., Ji Z., Haigh S. J., Holmes S. M. (2022). High-Performance
Polymer Electrolyte Membranes Incorporated with 2D Silica Nanosheets
in High-Temperature Proton Exchange Membrane Fuel Cells. J. Energy Chem..

[43] Komma M., Freiberg A. T. S., Abbas D., Arslan F., Milosevic M., Cherevko S., Thiele S., Böhm T. (2024). Applicability
of Single-Layer Graphene as a Hydrogen-Blocking Interlayer in Low-Temperature
PEMFCs. ACS Appl. Mater. Interfaces.

[44] Pavko L., Gatalo M., Finšgar M., Ruiz-Zepeda F., Ehelebe K., Kaiser P., Geuß M., D̵ukić T., Surca A. K., Šala M., Bele M., Cherevko S., Genorio B., Hodnik N., Gaberšček M. (2022). Graphene-Derived Carbon Support Boosts
Proton Exchange Membrane Fuel Cell Catalyst Stability. ACS Catal..

[45] Su H., Hu Y. H. (2021). Recent Advances in Graphene-Based Materials for Fuel
Cell Applications. Energy Sci. Eng..

[46] Yoon S. I., Seo D.-J., Kim G., Kim M., Jung C.-Y., Yoon Y.-G., Joo S. H., Kim T.-Y., Shin H. S. (2018). AA′-Stacked
Trilayer Hexagonal Boron Nitride Membrane for Proton Exchange Membrane
Fuel Cells. ACS Nano.

[47] Yoon S. I., Ma K. Y., Kim T.-Y., Shin H. S. (2020). Proton
Conductivity
of a Hexagonal Boron Nitride Membrane and Its Energy Applications. J. Mater. Chem. A.

[48] Yadav V., Kulshrestha V. (2019). Boron Nitride: A Promising Material for Proton Exchange
Membranes for Energy Applications. Nanoscale.

[49] An Y., Tian Y., Wei C., Zhang Y., Xiong S., Feng J., Qian Y. (2020). Recent Advances
and Perspectives
of 2D Silicon: Synthesis and Application for Energy Storage and Conversion. Energy Storage Mater..

[50] Shan G., Tan H., Ma R., Zhao H., Huang W. (2023). Recent Progress in
Emergent Two-Dimensional Silicene. Nanoscale.

[51] Son J., Lee S., Kim S. J., Park B. C., Lee H.-K., Kim S., Kim J. H., Hong B. H., Hong J. (2016). Hydrogenated Monolayer
Graphene with Reversible and Tunable Wide Band Gap and Its Field-Effect
Transistor. Nat. Commun..

[52] Tong J., Fu Y., Domaretskiy D., Della Pia F., Dagar P., Powell L., Bahamon D., Huang S., Xin B., Costa Filho R. N., Vega L. F., Grigorieva I. V., Peeters F. M., Michaelides A., Lozada-Hidalgo M. (2024). Control of Proton Transport and Hydrogenation in Double-Gated
Graphene. Nature.

[53] Morse J. R., Zugell D. A., Patterson E., Baldwin J. W., Willauer H. D. (2021). Hydrogenated
Graphene: Important Material Properties Regarding Its Application
for Hydrogen Storage. J. Power Sources.

[54] Fei Y., Fang S., Hu Y. H. (2020). Synthesis,
Properties and Potential
Applications of Hydrogenated Graphene. Chem.
Eng. J..

[55] Li C., Kang J., Xie J., Wang Y., Zhou L., Hu H., Li X., He J., Wang B., Zhang H. (2020). Two-Dimensional
Monoelemental Germanene Nanosheets: Facile Preparation and Optoelectronic
Applications. J. Mater. Chem. C.

[56] Ouyang J., Feng C., Ji X., Li L., Gutti H. K., Kim N. Y., Artzi D., Xie A., Kong N., Liu Y.-N., Tearney G. J., Sui X., Tao W., Farokhzad O. C. (2019). 2D Monoelemental Germanene Quantum Dots: Synthesis
as Robust Photothermal Agents for Photonic Cancer Nanomedicine. Angew. Chem., Int. Ed..

[57] Dávila M. E., Le Lay G. (2016). Few Layer Epitaxial Germanene: A Novel Two-Dimensional
Dirac Material. Sci. Rep..

[58] Bianco E., Butler S., Jiang S., Restrepo O. D., Windl W., Goldberger J. E. (2013). Stability and Exfoliation of Germanane: A Germanium
Graphane Analogue. ACS Nano.

[59] Yumigeta K., Brayfield C., Cai H., Hajra D., Blei M., Yang S., Shen Y., Tongay S. (2020). The Synthesis of Competing
Phase GeSe and GeSe2 2D Layered Materials. RSC
Adv..

[60] Lv X., Wei W., Mu C., Huang B., Dai Y. (2018). Two-Dimensional GeSe
for High Performance Thin-Film Solar Cells. J. Mater. Chem. A.

[61] Shin H., Krogel J. T., Gasperich K., Kent P. R. C., Benali A., Heinonen O. (2021). Optimized Structure and Electronic Band Gap of Monolayer
GeSe from Quantum Monte Carlo Methods. Phys.
Rev. Mater..

[62] Zhang T., Zhu L. (2024). Exceptionally Low Thermal
Conductivity in Simple Two-Dimensional
SiS: Anomalous Emergence of Rattling Phonon Modes in Non-Caged Materials. J. Mater. Chem. C.

[63] Yang J.-H., Zhang Y., Yin W.-J., Gong X. G., Yakobson B. I., Wei S.-H. (2016). Two-Dimensional SiS Layers with Promising Electronic
and Optoelectronic Properties: Theoretical Prediction. Nano Lett..

[64] Jiang H. R., Zhao T. S., Liu M., Wu M. C., Yan X. H. (2016). Two-Dimensional
SiS as a Potential Anode Material for Lithium-Based Batteries: A First-Principles
Study. J. Power Sources.

[65] Huang Z., Ren K., Zheng R., Wang L., Wang L. (2023). Ultrahigh Carrier Mobility
in Two-Dimensional IV−VI Semiconductors for Photocatalytic
Water Splitting. Molecules.

[66] Zhou Q., Liu L., Liu Q., Wang Z., Gao C., Liu Y., Ye H. (2020). Highly Selective Adsorption on SiSe
Monolayer and Effect of Strain
Engineering: A DFT Study. Sensors.

[67] Mao Y., Ben J., Yuan J., Zhong J. (2018). Tuning the Electronic Property of
Two Dimensional SiSe Monolayer by In-Plane Strain. Chem. Phys. Lett..

[68] Kamal C., Chakrabarti A., Ezawa M. (2016). Direct Band Gaps in Group IV-VI Monolayer
Materials: Binary Counterparts of Phosphorene. Phys. Rev. B.

[69] Jander, J. Recent Chemistry and Structure Investigation of Nitrogen Triiodide, Tribromide, Trichloride, and Related Compounds. In Adv. Inorg. Chem. Radiochem.; Emeléus, H. J. , Sharpe, A. G. , Eds.; Academic Press, 1976; Vol. 19, pp 1–63.

[70] Marinho G.
S., de Farias R. F. (2021). The Structure,
Thermodynamic Instability and Energetics
of NI3, Its Specific Impulse and a Strategy for Its Stabilization. J. Mol. Struct..

[71] Knisley, T. ; Woods, K. N. ; Saly, M. ; Winter, C. H. ; Upadhyay, A. Deposition of Tellurium-Containing Thin Films. U.S. Patent, 2022.1,140,8068 B2

[72] Sun G., Kürti J., Rajczy P., Kertesz M., Hafner J., Kresse G. (2003). Performance
of the Vienna Ab Initio Simulation Package
(VASP) in Chemical Applications. J. Mol. Struct.
THEOCHEM.

[73] Kresse G., Furthmüller J. (1996). Efficiency
of Ab-Initio Total Energy Calculations for
Metals and Semiconductors Using a Plane-Wave Basis Set. Comput. Mater. Sci..

[74] Hafner J. (2008). Ab-Initio
Simulations of Materials Using VASP: Density-Functional Theory and
Beyond. J. Comput. Chem..

[75] Blöchl P. E. (1994). Projector
Augmented-Wave Method. Phys. Rev. B.

[76] Jin Y., Le P. M. L., Gao P., Xu Y., Xiao B., Engelhard M. H., Cao X., Vo T. D., Hu J., Zhong L., Matthews B. E., Yi R., Wang C., Li X., Liu J., Zhang J.-G. (2022). Low-Solvation Electrolytes for High-Voltage
Sodium-Ion Batteries. Nat. Energy.

[77] Ong S. P., Richards W. D., Jain A., Hautier G., Kocher M., Cholia S., Gunter D., Chevrier V. L., Persson K. A., Ceder G. (2013). Python Materials Genomics
(Pymatgen): A Robust, Open-Source Python
Library for Materials Analysis. Comput. Mater.
Sci..

[78] Ouyang R., Curtarolo S., Ahmetcik E., Scheffler M., Ghiringhelli L. M. (2018). SISSOA
Compressed-Sensing Method for Identifying the
Best Low-Dimensional Descriptor in an Immensity of Offered Candidates. Phys. Rev. Mater..

