# Supplementary material for: Machine Learning−Accelerated Discovery of Proton-Conducting 2D Materials for Proton Exchange Membranes
Source: ACS Nano. 2025 Dec 27;20(1):719–30. doi: 10.1021/acsnano.5c15161 (PMC12810482; doi:10.1021/acsnano.5c15161)
Supplement: Supplementary file 1 [file nn5c15161_si_001.pdf]

Supporting Information

Machine Learning–Accelerated Discovery of Proton-Conducting

Yuting Li[1,2], Daniel Bahamon[1,2], Marcelo Lozada-Hidalgo[3], Nirpendra Singh[1,4], Andre K. Geim[3], Lourdes F. Vega[1,2,]*

[1] *Research & Innovation Center for Graphene and 2D Materials (RIC-2D), Khalifa University of Science and Technology, Abu Dhabi 127788, UAE*

[2] *Research and Innovation Center on CO_2 and Hydrogen (RICH Center) and Chemical and Petroleum Engineering Department, Khalifa University of Science and Technology, Abu Dhabi 127788, UAE*

[3] *Department of Physics and Astronomy and National Graphene Institute, University of Manchester, Manchester M13 9PL, U.K.*

[4] *Physics Department, Khalifa University of Science and Technology, Abu Dhabi 127788, UAE*

**Correspondence to: lourdes.vega@ku.ac.ae*

Table: Table S1-S4

Figure: Figure S1-S7

Table S1. Summary of 60 initial features used for machine learning models. The first column categorizes the features into three types: Geometrical (G), Electronic (e), and Energetic (E). The last column indicates whether the feature is derived from structural data and coordinate information (S), or obtained from the Mendel

Type	Abbreviation	Physical meaning	Source
G	*a_d_max*	Average of the maximum distances between neighbouring atoms within a single layer, averaged across all layers	S
	A	Surface area of simulation box	S
	a_vr	Average van der Waals radius	M
	atom_n_lay	Total number of atoms in the narrowest layer	S
	ave_d_lay	Average interatomic distance in the narrowest layer	S
	AvgL_d	Average interlayer spacing	S
	b	Average distance between atoms	S
	Channel	Whether atoms are arranged in a parallel manner in the front view, forming distinct channels for proton permeation	S
	cs	Symmetry of the crystal lattice	2D
	dm	In the narrowest layer, the minimum distance between neighboring atoms, when the interlayer spacing is 0.3 Å.	S
	dma	Average of the minimum distances between neighboring atoms within a single layer, averaged across all layers	S
	d_xy	Projected in-plane initial proton–surface distance. From the top view (*xy* plane), it is the distance between the proton and the material's atom closest to the proton	S
	d_xy_2	From the top view (*xy* plane), the distance between proton and the material's atom second closest to the proton	S
	fa	Formula anonymous	2D
	fuc_h	The presence of P, Ge, Br elements or functional groups of O-F, O-N, O-C, F-N, F-C, N-C	S
	L	The number of layers	S
	m	Average atomic mass	M
	max_d_lay3	In the widest layer, the maximum distance between the neighboring atoms, when the interlayer spacing is 0.3 Å.	S
	max_d_minlay	In the narrowest layer, the maximum distance between the neighboring atoms, when *the* interlayer spacing is 1 Å.	S
	Lm	Maximum interlayer spacing	S
	min_d_lay	In the narrowest layer, the minimum distance between the neighboring atoms, when the interlayer spacing is 1 Å.	S
	min_lay	The layer index of the narrowest layer	S
	min_lay_z	The *z* coordinate of the narrowest layer	S
	n	Number of atoms	S
	n_an	From the top view, the atomic number of the 2D-material's atom closest to the proton	M
	n_an_2	From the top view, the atomic number of the 2D-material's atom second closest to the proton	M
	n_cr	From the top view, the covalent radii of the 2D-material's atom closest to the proton	M
	n_cr_2	From the top view, the covalent radii of the 2D-material's atom second closest to the proton	M
	nele	The number of element types	2D
	Pa	Average pore size	S
	Pm	Maximum pore size	S

Type	Abbreviation	Physical meaning	Source
	r_c	Average covalent radius per atom	M
	r_i	Covalent radius of each element	M
	sg	Space group of the lattice	2D
	Stack_c	Stacking pattern of layers	S
	Stack_n	The number of different stacking arrangements	S
	x	The lattice cell size	S
	y	The lattice cell size	S
	z_a	The thickness of 2D layers	S
	a_dp	Average dipole polarizability	M
e	*ave_charge*	Average charge of all elements in the system	MP
	bc	Band center of density of states (DOS), the average energy of DOS within a specified energy range	2D
	bf	Band filling, the occupancy of the electronic states in the conduction or valence bands	2D
	BG	Band gap, the energy difference between the top of the valence band and the bottom of the conduction band	MP
	bk	Kurtosis of DOS, describes the shape of DOS	2D
	cbm	Conduction band minimum	2D
	charge_{i}	Charge of each element	MP
	ea	Average electron affinity per atom	M
	e_en	Average electronegativity per atom	M
	n_dp	From the top view, the dipole polarizability of the 2D-material's atom closest to the proton	M
	n_dp_2	From the top view, the dipole polarizability of the 2D-material's atom second closest to the proton	M
	n_ea	From the top view, the electron affinity of the 2D-material's atom closest to the proton	M
	n_ea_2	From the top view, the electron affinity of the 2D-material's atom second closest to the proton	M
	n_en	From the top view, the electronegativity of the 2D-material's atom closest to the proton	M
	n_en_2	From the top view, the electronegativity of the 2D-material's atom second closest to the proton	M
	vbm	Valence band maximum	2D
E	*E*	Total energy	2D
	E_d	Decomposition energy	2D
	E_v	van der Waals energy	2D
	E_x	Exfoliation energy	2D

Table S2. Machine learning studies for proton permeation prediction on 2D-materials. The machine learning models developed in this work are compared with existing ML studies

focusing on other types of 2D-materials. Marked in red font is the selected ML model used in this work for further analysis.

System	Main Features	ML model	Training Data	Error (eV)
This work	***Pore size, Interlayer structures, electron affinity, SISSI generated***	***RF***	***488***	***R^2:0.90 MAE: 0.07***
		PR		***R^2: 0.87 MAE: 0.07***
		DNN		***R^2: 0.88 MAE: 0.08***
Separation of H_2 over CH_4, N_2, H_2S, O_2, CO_2, and He, trade-off selectivity and permeability of CoRE-MOFMs ADDIN ZOTERO_ITEM CSL_CITATION {"citationID":"S3ELQvAi","properties":{"formattedCitation":"\\super 3\\nosupersub{}","plainCitation":"3","noteIndex":0},"citationItems":[{"id":300,"uris":["http://zotero.org/users/15250265/items/4K8PM73I"],"itemData":{"id":300,"type":"article-journal","abstract":"The high-throughput computational screening (HTCS) and machine learning (ML) were applied to evaluate the H2 separation performances of computation-ready, experimental metal–organic framework membranes (CoREMOFMs). For the separation of H2/X (X = CH4, N2, H2S, O2, CO2, and He), based on the results of the structureperformance relationships by univariate analysis found that the performance of the top-performing candidates far exceeded Robeson's upper bound. To evaluate the H2 permeability (PH2) and permselectivity, a new trade-off variable, the trade-off multiple selectivity and permeability (TMSP), was defined as the	Pore limiting diameters, large cavity diameter, volumetric surface area, porosity, density, pore size distribution %	BPNN,RF, SVM, GP, BAT, BOT, RL, SL	1000	R^2: 0.32-0.88 MAE: 537.46-166.626

process regression and random forest methods exhibited the first and second highest predictive abilities, respectively. Calculating the feature importance of each CoRE-MOFM to investigate the relationship between the number of features and the accuracy of the ML model. And a decision tree model was established following the principle of the minimum Gini coefficient, which would be accurately classified the CoRE-MOFMs based on the membrane structural descriptors; the optimal structural descriptors of MOF and the physical properties of the gas to be separated are used to establish a multivariate regression model which can predict the value of the optimal structural descriptors of MOFMs by knowing the physical properties of component X in the other H2/X separation. The quantitative structureperformance relationships on the microscopic scale could provide a theoretical basis for the screening of topperforming CoRE-MOFMs. The HTCS and ML methods could efficiently accelerate the design and the development of top-performing CoRE-MOFMs for H2 separation.","container-title":"Chemical Engineering Journal","DOI":"10.1016/j.cej.2022.136783","ISSN":"13858947","journalAbbreviation":"Chemical Engineering Journal","language":"en","page":"136783","source":"DOI.org (Crossref)","title":"Machine-Learning-Assisted High-Throughput computational screening of Metal–Organic framework membranes for hydrogen separation","URL":"https://linkinghub.elsevier.com/retrieve/pii/S1385894722022781","volume":"446","author":[{"family":"Bai","given":"Xiangning"},{"family":"Shi","given":"Zenan"},{"family":"Xia","given":"Huan"				

},{"family":"Li","given":"Shuhua"},{"family":"Liu","given":"Zili"},{"family":"Liang","given":"Hong"},{"family":"Liu","given":"Zhiting"},{"family":"Wang","given":"Bangfen"},{"family":"Qiao","given":"Zhiwei"}],"accessed":{"date-parts":[["2024",10,2]]},"issued":{"date-parts":[["2022",10]]}}}],"schema":"https://github.com/citation-style-language/schema/raw/master/csl				
CO adsorption energy on metal-nonmetal codoped graphene ADDIN ZOTERO_ITEM CSL_CITATION {"citationID":"qIwDOtM5","properties":{"formattedCitation":"\\super 4\\nosupersub{}","plainCitation":"4","noteIndex":0},"citationItems":[{"id":"BJBRhy7I/gvtmlz3i","uris":["http://www.mendeley.com/documents/?uuid=e0b7e7b0-00d1-484e-a1d6-e87a7601e355"],"itemData":{"DOI":"10.1021/acs.jpcc.0c05964","ISSN":"19327455","abstract":"Electroreduction of CO2 is one of the most potential ways to realize CO2 recycle and energy regeneration. The key to promoting this technology is	atomic number, radius, electronegativity, d-electron, ionization energy	XGBR	171	R^2: 0.79-0.90 RMSE: 0.23-0.16

respect to such a screening model. This work demonstrates the potential of machine learning methods and provides a convenient approach for the effective theoretical design of electrocatalysts for CO2 reduction.","author":[{"dropping-particle":"","family":"Chen","given":"An","non-dropping-particle":"","parse-names":false,"suffix":""},{"dropping-particle":"","family":"Zhang","given":"Xu","non-dropping-particle":"","parse-names":false,"suffix":""},{"dropping-particle":"","family":"Chen","given":"Letian","non-dropping-particle":"","parse-names":false,"suffix":""},{"dropping-particle":"","family":"Yao","given":"Sai","non-dropping-particle":"","parse-names":false,"suffix":""},{"dropping-particle":"","family":"Zhou","given":"Zhen","non-dropping-particle":"","parse-names":false,"suffix":""}],"container-title":"Journal of Physical Chemistry C","id":"ITEM-1","issue":"41","issued":{"date-parts":[["2020"]]},"page":"22471-22478","title":"A Machine Learning Model on Simple Features for CO2Reduction Electrocatalysts","type":"article-journal","volume":"124"}}],"schema":"https://github.com/citation-style-language/schema/raw/master/cs				
Hydroxide ion (OH^-) conductivity of poly-based a n i o n e x c h a	chemical structure information	DNN	592, 211, 248	RMSE: 0.016-0.009

journal","abstract":"Possessing high ionic conductivity is required to polymer-based membrane electrolytes. However, it is a challenge to evaluate the conductivity based on the structure of the polymer membrane without any measurements. We present a deep learning protocol to predict the hydroxide ion (OH-) conductivity from chemical structure information of poly (2,6-dimethyl phenylene oxide)-based anion exchange membranes (AEMs) grafting with one kind of functional cationic group. The modeling process includes data collection and feature processing, functional cationic group identification, OH- conductivity prediction and scientific law extraction. The established model achieves 99.7% of accuracy for classifying various functional cationic groups. The prediction error in OHconductivity is ± 0.016 S/cm for quaternary ammonium based AEMs, ± 0.014 S/cm for saturated heterocyclic ammonium based ones, and ± 0.07 S/cm for those possessing imidazolium cations. The proposed protocol is powerful to assist researchers in designing the AEMs with predictable OH- conductivity, and provides a new research paradigm of the AEMs preparation.","container-title":"Journal of Membrane Science","DOI":"10.1016/j.memsci.2021.119983","ISSN":"03767388","journalAbbreviation":"Journal of Membrane Science","language":"en","page":"119983","source":"DOI.org (Crossref)","title":"A deep learning protocol for analyzing and predicting ionic conductivity of anion exchange membranes","URL":"https://linkinghub.elsevier.com/retrieve/pii/S0376738821009261","volume":"642","author":[{"family":"Zhai","given":"Fu-Heng"},{"family":"Zhan","given":"Qing-				

Qing"},{"family":"Yang","given":"Yun-Fei"},{"family":"Ye","given":"Ni-Ya"},{"family":"Wan","given":"Rui-Ying"},{"family":"Wang","given":"Jin"},{"family":"Chen","given":"Shuai"},{"family":"He","given":"Rong-Huan"}],"accessed":{"date-parts":[["2024",11,17]]},"issued":{"date-parts":[["2022",2]]}}}],"schema":"https://github.com/citation-style-language/schema/raw/master/csl				
Gas permeabilities and selectivities of CO_2/CH_4 and CO_2/N_2 ADDIN ZOTERO_ITEM CSL_CITATION {"citationID":"iQZyasG7","properties":{"formattedCitation":"\\super 6\\nosupersub{}","plainCitation":"6","noteIndex":0},"citationItems":[{"id":45,"uris":["http://zotero.org/users/15250265/items/EZFUZR7I"],"item	polymer fingerprints, environment conditions	BLR, ERT	620, 608, 102, 123, 165, 341	RMSE: 0.23-0.86

","ISSN":"03767388","journalAbbreviation":"Journal of Membrane Science","language":"en","page":"119207","source":"DOI.org (Crossref)","title":"Imputation of missing gas permeability data for polymer membranes using machine learning","URL":"https://linkinghub.elsevier.com/retrieve/pii/S0376738821001575","volume":"627","author":[{"family":"Yuan","given":"Qi"},{"family":"Longo","given":"Mariagiulia"},{"family":"Thornton","given":"Aaron W."},{"family":"McKeown","given":"Neil B."},{"family":"Comesaña-Gándara","given":"Bibiana"},{"family":"Jansen","given":"Johannes C."},{"family":"Jelfs","given":"Kim E."}],"accessed":{"date-parts":[["2024",10,1]]},"issued":{"date-parts":[["2021",6]]}}}],"schema":"https://github.com/citation-style-l				
Band gap and dielectric constant of 2D field-effect transistor	mean atomic mass, cations electronegativity, anions electronegativity, L2 norm of elemental fractions, cluster dimensionality, packing fraction	XGBoost	522	Accuracy exceeding 80%

MoS2-based FETs. Element prevalence analysis indicates that materials containing strongly electronegative anions and heavy cations are more likely to be promising dielectrics. Moreover, we developed a high-accuracy two-step machine learning (ML) classifier for screening dielectrics. Implementing active learning framework, we successfully identified 49 additional promising vdW dielectrics. This work provides a rich candidate list of vdW dielectrics along with a high-accuracy ML screening model, facilitating future development of 2D FETs.","container-title":"Nature Communications","DOI":"10.1038/s41467-024-53864-4","ISSN":"2041-1723","issue":"1","journalAbbreviation":"Nat Commun","language":"en","license":"2024 The Author(s)","note":"publisher: Nature Publishing Group","page":"9527","source":"www.nature.com","title":"High-throughput screening and machine learning classification of van der Waals dielectrics for 2D nanoelectronics","URL":"https://www.nature.com/articles/s41467-024-53864-4","volume":"15","author":[{"family":"Li","given":"Yuhui"},{"family":"Wan","given":"Guolin"},{"family":"Zhu","given":"Yongqian"},{"family":"Yang","given":"Jingyu"},{"family":"Zhang","given":"Yan-Fang"},{"family":"Pan","given":"Jinbo"},{"family":"Du","given":"Shixuan"}],"accessed":{"date-parts":[["2024",12,6]]},"issued":{"date-parts":[["2024",11,4]]}}}],"schema":"https://github.com/citation-style-language/schema/raw/master/csl				
CO_2	Organic, inorganic, topology, functionality	RF, RR, SVM	100	MAE: 0.46-4

perties":{"formattedCitation":"\\super 8\\nosupersub{}","plainCitation":"8","noteIndex":0},"citationItems":[{"id":2184,"uris":["http://zotero.org/users/15250265/items/7M9ZYU7I"],"itemData":{"id":2184,"type":"article-journal","abstract":"A novel computational methodology for large-scale screening of MOFs is applied to gas storage with the use of machine learning technologies. This approach is a promising trade-off between the accuracy of ab initio methods and the speed of classical approaches, strategically combined with chemical intuition. The results demonstrate that the chemical properties of MOFs are indeed predictable (stochastically, not deterministically) using machine learning methods and automated analysis protocols, with the accuracy of predictions increasing with sample size. Our initial results indicate that this methodology is promising to apply not only to gas storage in MOFs but in many other material science projects.","container-title":"npj Computational Materials","DOI":"10.1038/s41524-017-0045-8","ISSN":"2057-3960","issue":"1","journalAbbreviation":"npj Comput Mater","language":"en","license":"2017 The Author(s)","note":"publisher: Nature Publishing Group","page":"1-7","source":"www.nature.com","title":"Chemically intuited, large-scale screening of MOFs by machine learning techniques","URL":"https://www.nature.com/articles/s41524-017-0045-				

"George E."}],"accessed":{"date-parts":[["2024",12,3]]},"issued":{"date-parts":[["2017",10,2]]}}}],"schema":"https://github.com/citation-style-language/schema/raw/master/csl				
six gas separations: He/H_2, He/N_2, He/CH_4, H_2/N_2, H_2/CH_4, and N_2/CH_4 of MOFs and MOF/polymer MMMs ADDIN ZOTERO_ITEM CSL_CITATION {"citationID":"130uTsGK","properties":{"formattedCitation":"\\super 9\\nosupersub{}","plainCitation":"9","noteIndex":0},"citationItems":[{"id":41,"uris":["http://zotero.org/users/15250265/items/IEZUAYRK"],"itemData":{"id	pore size, pore geometry, atom types, metallic percentage, and heat of adsorption of gases	*TPOT*	5249, 31,494	Adsorption: R^2: 0.73-0.99 Diffusion: R^2: 0.65-0.80

31,494 MOF/polymer MMMs were predicted using these ML models. To examine the transferability of the ML models, we also focused on computer-generated, hypothetical MOFs (hMOFs) and predicted the gas permeability and selectivity of 1000 hMOF/polymer MMMs. The ML models that we developed accurately predict the uptake and diffusion properties of He, H2, N2, and CH4 gases in MOFs and will significantly accelerate the assessment of separation performances of MOF membranes and MOF/polymer MMMs. These models will also be useful to direct the extensive experimental efforts and computationally demanding molecular simulations to the fabrication and analysis of membrane materials offering high performance for a target gas separation.","container-title":"ACS Applied Materials & Interfaces","DOI":"10.1021/acsami.2c08977","ISSN":"1944-8244, 1944-8252","issue":"28","journalAbbreviation":"ACS Appl. Mater. Interfaces","language":"en","license":"https://creativecommons.org/licenses/by/4.0/","page":"32134-32148","source":"DOI.org (Crossref)","title":"Combining Machine Learning and Molecular Simulations to Unlock Gas Separation Potentials of MOF Membranes and MOF/Polymer MMMs","URL":"https://pubs.acs.org/doi/10.1021/acsami.2c08977","volume":"14","author":[{"family":"Daglar","given":"Hilal"},{"family":"Keskin","given":"Seda"}],"accessed":{"date-parts":[["2024",10,1]]},"issued":{"date-parts":[["2022",7,20]]}}}],"schema":"https://github.com/citation-style-language/schema/raw/master/csl				

BLR: Bayesian Linear Regression; BPNN: back propagation neural network; SVM: support vector machine; BAT: bagged tree; BOT: boosted tree; SL: stepwise linear regression; RL: Linear regression; DNN: Deep Neural Network; XGBR: extreme gradient boosting regression; RR: ridge regression; TPOT : tree-based pipeline optimization tool; MAE: mean absolute error, closer to 0 indicates better performance; RMSE: root mean squared error, closer to 0 indicates better performance; R^2*: the coefficient of determination, closer to 1 indicates better performance.*

Table S3. **Key properties and descriptions of screened materials for PEMs.** Ea means the activation energy for proton or hydrogen molecule permeation through the material. The detailed material properties include unique ID, crystal structure, pore size, atomic distances, electron affinity, band gap, and types of neighboring atoms. References related to materials, such as theoretical (DFT), experimental (EXP), or practical application (PEM). The presence of reference numbers indicates that studies have been found in the respective field; the absence implies no relevant literature has been identified. The materials that have been widely studied in the literature for PEM are highlighted in grey color, while the materials only investigated as theoretical stable structures are highlighted in blue color.

**Name**	**Ea_H$^+$ (eV)**	**Ea_H$_2$ (eV)**	**Front view (YZ)**	**Top view (XY)**	**Information (ID, Name, Pore size, dma, Electron affinity, band gap, Neighbour atom)**	**Ref**
Si	0.43	3.95	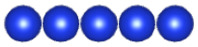	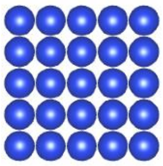	60ef73284dbb56fa22f3da3d, diamond cubic Silicon, 3.68 Å^2, 2.42 Å, 1.39 eV, 0 eV, Si	DFT EXP ADDIN ZOTERO_ITEM CSL_CITATION {"citationID":"tEJ4A2YK","properties":{"formattedCitation":"\\super 10,11\\nosupersub{}","plainCitation":"10,11","noteIndex":0},"citationItems":[{"id":2006,"uris":["http://zotero.org/users/15250265/items/

Name	Ea_H^+ (eV)	Ea_H_2 (eV)	Front view (YZ)	Top view (XY)	Information (ID, Name, Pore size, dma, Electron affinity, band gap, Neighbour atom)	Ref
						"id":2006,"type":"article-journal","abstract":"In this paper, molecular dynamic simulations have been performed to fabricate nanopores in a polycrystalline boron-nitride nanosheet applied to DNA sequencer devices by using Si clusters bombardment. Three different sizes of Si clusters with ten different kinetic energies and impacts at five different locations of the polycrystalline boron-nitride nanosheet have been simulated. Our

Name	Ea_H^+ (eV)	Ea_H_2 (eV)	Front view (YZ)	Top view (XY)	Information (ID, Name, Pore size, dma, Electron affinity, band gap, Neighbour atom)	Ref
						results show that desired nanopore s with expected size and topograp hy can be created by controlli ng the kinetic energy and size of the cluster. The area size of nanopore s also increase by rising the kinetic energy of the cluster. We have also observed that the existing grain boundary in the incident location highly affects the shape and size of the nanopore s. Therefor e, we subseque ntly applied an external tensile strain on the

Name	Ea_H^+ (eV)	Ea_H_2 (eV)	Front view (YZ)	Top view (XY)	Information (ID, Name, Pore size, dma, Electron affinity, band gap, Neighbour atom)	Ref
						boron-nitride nanosheet and determined the effect of straining nanosheet on the area, quality and the shape of fabricated nanopores. We find that increasing the external tensile strain leads to a large increase in the area of nanopores, but the shape and quality of fabricated nanopores remains nearly unaffected, particularly compared to drilling nanopores in the unstrained nanosheet. In order to investigate the effect of

Name	Ea_H+ (eV)	Ea_H2 (eV)	Front view (YZ)	Top view (XY)	Information (ID, Name, Pore size, dma, Electron affinity, band gap, Neighbour atom)	Ref
						the cluster type, two new type of clusters (SiC and diamond) have been used to generate nanopores. Our results reveal that SiC and diamond clusters bombardment lead to fabricate almost the same shape and quality of nanopores as well as the Si cluster. On the other hand, increasing the kinetic energy of the SiC and diamond cluster barely influences the area size of the nanopores. Among all clusters, the diamond cluster

Name	Ea_H⁺ (eV)	Ea_H_2 (eV)	Front view (YZ)	Top view (XY)	Information (ID, Name, Pore size, dma, Electron affinity, band gap, Neighbour atom)	Ref
						bombardment leads to fabricate the largest average area size of the nanopores.","container-title":"Computational Materials Science","DOI":"10.1016/j.commatsci.2017.12.022","ISSN":"09270256","journalAbbreviation":"Computational Materials Science","language":"en","page":"280-290","source":"DOI.org (Crossref)","title":"Fabrication of nanopores in polycrystalline boron-nitride nanosheet by using Si, SiC and diamond clusters bombardment","U

Name	Ea_H⁺ (eV)	Ea_H_2 (eV)	Front view (YZ)	Top view (XY)	Information (ID, Name, Pore size, dma, Electron affinity, band gap, Neighbour atom)	Ref
						RL":"https://linkinghub.elsevier.com/retrieve/pii/S0927025617307073","volume":"145","author":[{"family":"Abadi","given":"Rouzbeh"},{"family":"Nezhad Shirazi","given":"Ali Hossein"},{"family":"Izadifar","given":"Mohammadreza"},{"family":"Sepahi","given":"Mohammad"},{"family":"Rabczuk","given":"Timon"}],"accessed":{"date-parts":[["2024",12,1]]},"issued":{"date-parts":[["2018",4]]}}},{"id":2004,"uris":["http://zotero.org/users/15250265/items/UV8HG7ZD"],"it

Name	Ea_H$^+$ (eV)	Ea_H_2 (eV)	Front view (YZ)	Top view (XY)	Information (ID, Name, Pore size, dma, Electron affinity, band gap, Neighbour atom)	Ref
						emData":{"id":2004,"type":"article-journal","abstract":"Silicon (Si), associated with its natural abundance, low discharge voltage vs. Li/Li+, and extremely high theoretical capacity (4200 mAh g 1,), has been extensively explored as anode for lithium ion battery. One of the key challenges for using Si as anode is the large volume change upon lithiation and delithiation, which causes a fast capacity fading. Over the

Name	Ea_H$^+$ (eV)	Ea_H_2 (eV)	Front view (YZ)	Top view (XY)	Information (ID, Name, Pore size, dma, Electron affinity, band gap, Neighbour atom)	Ref
						last few years, dramatic progress has been made for addressin g this issue. In this paper, we review the progress towards tailoring of Si as anode for lithium ion battery. The paper is organize d such that it covers the fundame ntals, the promises offered by nanoscal e designs, and the challenge s that remained to be addresse d to allow the applicati on of Si based materials as high capacity anode for lithium ion batteries. ","contai ner-

Name	Ea_H$^+$ (eV)	Ea_H_2 (eV)	Front view (YZ)	Top view (XY)	Information (ID, Name, Pore size, dma, Electron affinity, band gap, Neighbour atom)	Ref
						title":"Nano Energy","DOI":"10.1016/j.nanoen.2015.08.025","ISSN":"22112855","journalAbbreviation":"Nano Energy","language":"en","page":"366-383","source":"DOI.org (Crossref)","title":"Nanoscale silicon as anode for Li-ion batteries: The fundamentals, promises, and challenges","title-short":"Nanoscale silicon as anode for Li-ion batteries","URL":"https://linkinghub.elsevier.com/retrieve/pii/S221128551500350X","volume":"17","author":[{"family":"Gu","given":"

Name	Ea_H⁺ (eV)	Ea_H_2 (eV)	Front view (YZ)	Top view (XY)	Information (ID, Name, Pore size, dma, Electron affinity, band gap, Neighbour atom)	Ref
						Meng"},{"family":"He","given":"Yang"},{"family":"Zheng","given":"Jianming"},{"family":"Wang","given":"Chongmin"}],"accessed":{"date-parts":[["2024",12,1]]},"issued":{"date-parts":[["2015",10]]}}}],"schema":"https://github.com/citation-style-language/schema/raw/maste PEM
TeC	0.41	6.29	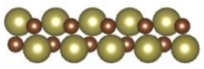	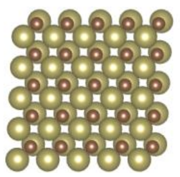	60ef71a6c403a99258a82bb5, Tellurium Carbide, 0.52 Å², 2.14 Å, 1.62 eV, 0.65 eV, Te	DFT EXP ADDIN ZOTERO_ITEM CSL_CITATION {"citationID":"H3ARp8j6","properties":{"formattedCitation":"\\super 12\\nosupersub{}","plainCitation":"12","note

Name	Ea_H+ (eV)	Ea_H_2 (eV)	Front view (YZ)	Top view (XY)	Information (ID, Name, Pore size, dma, Electron affinity, band gap, Neighbour atom)	Ref
						{"id":2015,"uris":["http://zotero.org/users/15250265/items/2HYYWU2W"],"itemData":{"id":2015,"type":"patent","authority":"United States","call-number":"US17163687","language":"en","number":"US11408068B2","title":"Deposition of tellurium-containing thin films","URL":"https://patents.google.com/patent/US11408068B2/en","author":[{"family":"Knisley","given":"Thomas"},{"family":"Woods","given":"Keenan N."},{"family":"Saly","given":"Mark"},{"family":"

Name	Ea_H$^+$ (eV)	Ea_H_2 (eV)	Front view (YZ)	Top view (XY)	Information (ID, Name, Pore size, dma, Electron affinity, band gap, Neighbour atom)	Ref
						Winter","given":"Charles H."},{"family":"Upadhyay","given":"Apoorva"}],"accessed":{"date-parts":[["2024",12,1]]},"issued":{"date-parts":[["2022",8,9]]},"submitted":{"date-parts":[["2021",2,1]]}}}],"schema":"https://github.com/citation-style-language/schema/raw/mast
Si	0.52	3.20	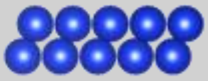	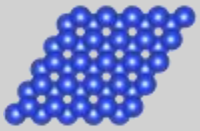	60ef73244dbb56fa22f3da12, Silicene, 0.78 Å^2, 2.78 Å, 1.39 eV, 0 eV, Si	PEM DFT ADDIN ZOTERO_ITEM CSL_CITATION {"citationID":"ID7WcvoT","properties":{"formattedCitation":"\\super 13,14\\nosupersub{}","plainCitation":"13,14","noteInd

Name	Ea_H+ (eV)	Ea_H2 (eV)	Front view (YZ)	Top view (XY)	Information (ID, Name, Pore size, dma, Electron affinity, band gap, Neighbour atom)	Ref
						":1996,"uris":["http://zotero.org/users/15250265/items/W5UVDNZC"],"itemData":{"id":1996,"type":"article-journal","abstract":"Two-dimensional silicon crystals with sizeable band gaps and ultrahigh carrier mobility are reported by using first-principles calculations.\n , \n \n Due to their compatibility in the well-developed Si-based semiconductor industry, exploring two-dimensional (2D) silicon crystals with both sizable band

Name	Ea_H+ (eV)	Ea_H_2 (eV)	Front view (YZ)	Top view (XY)	Information (ID, Name, Pore size, dma, Electron affinity, band gap, Neighbour atom)	Ref
						gaps and high carrier mobility is important to develop high-performance electronic and optoelectronic devices on the nanoscale. Here, eleven new 2D silicon crystals are reported based on the strategy of mixing 3-fold and 4-fold coordinated silicon atoms in 2D confined phases and first-principles calculations. We establish that these 2D silicon crystals can be obtained by functionalizing silicene with

Name	Ea_H+ (eV)	Ea_H_2 (eV)	Front view (YZ)	Top view (XY)	Information (ID, Name, Pore size, dma, Electron affinity, band gap, Neighbour atom)	Ref
						silicon atoms, dimers, or chains, which exhibit lower formation energy than that of silicene. Their dynamic stability and thermal stability are confirmed by phonon calculations and Born–Oppenheimer molecular dynamic simulation at temperatures up to 700 K. Electronic structure calculations reveal that these 2D silicon crystals are semiconductors with sizable and tunable band gaps, ranging

Name	Ea_H+ (eV)	Ea_H2 (eV)	Front view (YZ)	Top view (XY)	Information (ID, Name, Pore size, dma, Electron affinity, band gap, Neighbour atom)	Ref
						from 1.12 to 1.67 eV, and four of them are direct or quasi-direct band gap semiconductors with strong absorption in the visible-light frequency. The calculated Young's stiffness of 2D silicon crystals ranges from 31 to 88 N m\n −1\n , which are comparable to phosphorene, but remarkably smaller than those of MoS\n 2\n monolayer and graphene. Remarkably, C\n z\n -\n P\n 2/\n c\n -Si\n

Name	Ea_H^+ (eV)	Ea_H_2 (eV)	Front view (YZ)	Top view (XY)	Information (ID, Name, Pore size, dma, Electron affinity, band gap, Neighbour atom)	Ref
						12\n possesses a negative Poisson's ratio with a maximum value of −0.055. In particular, 2D silicon crystals possess ultrahigh carrier mobility of up to 1.7 × 10\n 5\n and 1.3 × 10\n 4\n cm\n 2\n V\n −1\n s\n −1\n at room temperature for electrons and holes, respectively, suitable for high-speed electronic and optoelectronic applications on the nanoscale.","container-title":"Nanoscale",

Name	Ea_H+ (eV)	Ea_H_2 (eV)	Front view (YZ)	Top view (XY)	Information (ID, Name, Pore size, dma, Electron affinity, band gap, Neighbour atom)	Ref
						"DOI":"10.1039/C7NR08172C","ISSN":"2040-3364, 2040-3372","issue":"3","journalAbbreviation":"Nanoscale","language":"en","page":"1265-1271","source":"DOI.org (Crossref)","title":"Two-dimensional silicon crystals with sizable band gaps and ultrahigh carrier mobility","URL":"https://xlink.rsc.org/?DOI=C7NR08172C","volume":"10","author":[{"family":"Zhuo","given":"Zhiwen"},{"family":"Wu","given":"Xiaojun"},{"family":"Yang","given":"Jinlong"}],"accesse

Name	Ea_H^+ (eV)	Ea_H_2 (eV)	Front view (YZ)	Top view (XY)	Information (ID, Name, Pore size, dma, Electron affinity, band gap, Neighbour atom)	Ref
						d":{"date-parts":[["2024",12,1]]},"issued":{"date-parts":[["2018"]]}}},{"id":2028,"uris":["http://zotero.org/users/15250265/items/SPDXXW4A"],"itemData":{"id":2028,"type":"article-journal","abstract":"By using ab initio calculations, we predict that a vertical electric field is able to open a band gap in semimetallic single-layer buckled silicene and germanene. The sizes of the band gap in both silicene and germanene increase

Name	Ea_H+ (eV)	Ea_H_2 (eV)	Front view (YZ)	Top view (XY)	Information (ID, Name, Pore size, dma, Electron affinity, band gap, Neighbour atom)	Ref
						linearly with the electric field strength. Ab initio quantum transport simulation of a dual-gated silicene field effect transistor confirms that the vertical electric field opens a transport gap, and a significant switching effect by an applied gate voltage is also observed. Therefore, biased single-layer silicene and germanene can work effectively at room temperature as field effect transistors.","container-title":"Nano Letters","

Name	Ea_H$^+$ (eV)	Ea_H_2 (eV)	Front view (YZ)	Top view (XY)	Information (ID, Name, Pore size, dma, Electron affinity, band gap, Neighbour atom)	Ref
						DOI":"10.1021/nl203065e","ISSN":"1530-6984","issue":"1","journalAbbreviation":"Nano Lett.","language":"en-US","note":"publisher: American Chemical Society","page":"113-118","source":"ACS Publications","title":"Tunable Bandgap in Silicene and Germanene","URL":"https://doi.org/10.1021/nl203065e","volume":"12","author":[{"family":"Ni","given":"Zeyuan"},{"family":"Liu","given":"Qihang"},{"family":"Tang","given":"Kechao"},{"famil

Name	Ea_H^+ (eV)	Ea_H_2 (eV)	Front view (YZ)	Top view (XY)	Information (ID, Name, Pore size, dma, Electron affinity, band gap, Neighbour atom)	Ref
						y":"Zheng","given":"Jiaxin"},{"family":"Zhou","given":"Jing"},{"family":"Qin","given":"Rui"},{"family":"Gao","given":"Zhengxiang"},{"family":"Yu","given":"Dapeng"},{"family":"Lu","given":"Jing"}],"accessed":{"date-parts":[["2024",12,1]]},"issued":{"date-parts":[["2012",1,11]]}}}],"schema":"https://github.com/citation-style-language/schema/raw/mast E X P ADDIN ZOTERO_ITEM CSL_CITATION {"citationID":"J0uxvbtV","properties":{"formattedCi

Name	Ea_H^+ (eV)	Ea_H_2 (eV)	Front view (YZ)	Top view (XY)	Information (ID, Name, Pore size, dma, Electron affinity, band gap, Neighbour atom)	Ref
						supersub{}","plainCitation":"15,16","noteIndex":0},"citationItems":[{"id":2000,"uris":["http://zotero.org/users/15250265/items/35TIYFQB"],"itemData":{"id":2000,"type":"article-journal","abstract":"Two-dimensional (2D) materials such as graphene have sparked great attention and research in every field. Among them, 2D silicon has wide potential applications in chemical sensor, hydrogen storage, semiconductors, electronic device, biomedicine and energy storage and

Name	Ea_H+ (eV)	Ea_H2 (eV)	Front view (YZ)	Top view (XY)	Information (ID, Name, Pore size, dma, Electron affinity, band gap, Neighbour atom)	Ref
						conversi on due to its abundant resources , environm entfriend ly character , unique structure and superior propertie s. Specially , with the fast ion transport, sufficient electrode - electrolyt e contact area, huge theoretic al capacity and superior activity, 2D silicon shows promisin g applicati ons in energy storage and conversi on such as lithium-ion batteries, lithium-oxygen batteries, lithiumsu lfur batteries, sodium-

Name	Ea_H^+ (eV)	Ea_H_2 (eV)	Front view (YZ)	Top view (XY)	Information (ID, Name, Pore size, dma, Electron affinity, band gap, Neighbour atom)	Ref
						ion batteries, potassium-ion batteries, magnesium-ion batteries, supercapacitor, hydrogen storage, etc. However, the challenges such as complicated synthesis process, sensitive in atmosphere, strong Van der Waals force between silicene hinder the commercialization of 2D silicon. Several effective strategies have been designed and developed on 2D silicon. In this review, we comprehensively summarize the fundamentals, advances

Name	Ea_H+ (eV)	Ea_H2 (eV)	Front view (YZ)	Top view (XY)	Information (ID, Name, Pore size, dma, Electron affinity, band gap, Neighbour atom)	Ref
						in synthesis of 2D silicon and its application in energy storage and conversion. Eight strategies for fabrication of 2D silicon are presented and analyzed, focusing on the relationships among fabrication approaches, intrinsic structure and properties. Furthermore, this review offers a state-of-the-art development and perspectives in synthesis and energy storage applications. The vast application of 2D silicon can be a

Name	Ea_H+ (eV)	Ea_H2 (eV)	Front view (YZ)	Top view (XY)	Information (ID, Name, Pore size, dma, Electron affinity, band gap, Neighbour atom)	Ref
						new milepost for energy storage and conversion and other aspects. In addition, the content of reviews may be referred by other 2D materials. We hope that the simplified synthesis process, improved and unique properties might promote the practical applications of 2D silicon in energy science and beyond.","container-title":"Energy Storage Materials","DOI":"10.1016/j.ensm.2020.07.006","ISSN":"2405

Name	Ea_H^+ (eV)	Ea_H_2 (eV)	Front view (YZ)	Top view (XY)	Information (ID, Name, Pore size, dma, Electron affinity, band gap, Neighbour atom)	Ref
						8297","journalAbbreviation":"Energy Storage Materials","language":"en","page":"115-150","source":"DOI.org (Crossref)","title":"Recent advances and perspectives of 2D silicon: Synthesis and application for energy storage and conversion","title-short":"Recent advances and perspectives of 2D silicon","URL":"https://linkinghub.elsevier.com/retrieve/pii/S2405829720302713","volume":"32","author":[{"family":"An","given":"Yongling"},{"fami

Name	Ea_H$^+$ (eV)	Ea_H_2 (eV)	Front view (YZ)	Top view (XY)	Information (ID, Name, Pore size, dma, Electron affinity, band gap, Neighbour atom)	Ref
						ly":"Tian","given":"Yuan"},{"family":"Wei","given":"Chuanliang"},{"family":"Zhang","given":"Yuchan"},{"family":"Xiong","given":"Shenglin"},{"family":"Feng","given":"Jinkui"},{"family":"Qian","given":"Yitai"}],"accessed":{"date-parts":[["2024",12,1]]},"issued":{"date-parts":[["2020",11]]}}},{"id":2032,"uris":["http://zotero.org/users/15250265/items/TF35GSJQ"],"itemData":{"id":2032,"type":"article-journal","abstract":"Although graphene is by far the most famous example

Name	Ea_H⁺ (eV)	Ea_H₂ (eV)	Front view (YZ)	Top view (XY)	Information (ID, Name, Pore size, dma, Electron affinity, band gap, Neighbour atom)	Ref
						of two-dimensional (2D) materials, which exhibits a wealth of exotic and intriguing properties, it suffers from a severe drawback. In this regard, the exploration of silicene, the silicon analog of the graphene material, has attracted substantial interest in the past decade. This review therefore provides a comprehensive survey of recent theoretical and experimental works on this 2D material. We first overview the

Name	Ea_H^+ (eV)	Ea_H_2 (eV)	Front view (YZ)	Top view (XY)	Information (ID, Name, Pore size, dma, Electron affinity, band gap, Neighbour atom)	Ref
						distinctive structures and properties of silicene, including mechanical, electronic, and spintronic properties. We then discuss the growth and experimental characterization of silicene on Ag(111) and other different substrates, providing insights into the different phases or atomic arrangements of silicene observed on the metallic surfaces as well as on its electronic structures. Then, the recent state-of-the-art applicati

Name	Ea_H$^+$ (eV)	Ea_H_2 (eV)	Front view (YZ)	Top view (XY)	Information (ID, Name, Pore size, dma, Electron affinity, band gap, Neighbour atom)	Ref
						ons of silicene are summarized in section 4 with the aim to break the scientific and engineering barriers for application in nanoelectronics, sensors, energy storage devices, electrode materials, and quantum technology. Finally, the concluding remarks and the future prospects of silicene are also provided. ","container-title":"Nanoscale","DOI":"10.1039/D2NR05809J","ISSN":"2040-3372","issue":"7","journalAbbreviation":"Na

Name	Ea_H$^+$ (eV)	Ea_H_2 (eV)	Front view (YZ)	Top view (XY)	Information (ID, Name, Pore size, dma, Electron affinity, band gap, Neighbour atom)	Ref
						noscale","language":"en","note":"publisher: The Royal Society of Chemistry","page":"2982-2996","source":"pubs.rsc.org","title":"Recent progress in emergent two-dimensional silicene","URL":"https://pubs.rsc.org/en/content/articlelanding/2023/nr/d2nr05809j","volume":"15","author":[{"family":"Shan","given":"Guangcun"},{"family":"Tan","given":"Haoyi"},{"family":"Ma","given":"Ruguang"},{"family":"Zhao","given":"Hongbin"},{"family":"

Name	Ea_H$^+$ (eV)	Ea_H$_2$ (eV)	Front view (YZ)	Top view (XY)	Information (ID, Name, Pore size, dma, Electron affinity, band gap, Neighbour atom)	Ref
						":{"date-parts":[["2024",12,1]]},"issued":{"date-parts":[["2023",2,16]]}}}],"schema":"https://github.com/citation-style-language/schema/raw/mast P E M ADDIN ZOTERO_ITEM CSL_CITATION {"citationID":"cdu8COfX","properties":{"formattedCitation":"\\super 17\\nosupersub{}","plainCitation":"17","noteIndex":0},"citationItems":[{"id":1998,"uris":["http://zotero.org/users/15250265/items/JF3YA3IG"],"itemData":{"id":1998,"type":"article-journal",

Name	Ea_H$^+$ (eV)	Ea_H_2 (eV)	Front view (YZ)	Top view (XY)	Information (ID, Name, Pore size, dma, Electron affinity, band gap, Neighbour atom)	Ref
						ts (SN) derived from natural vermiculite (Verm) were successfully incorporated into polyether sulfone–polyvinyl pyrrolidone (PES–PVP) polymer to fabricate high–temperature proton exchange membranes (HT–PEMs). The content of SN filler was varied (0.1–0.75 wt%) to study its influence on proton conductivity, power density and durability. Benefiting from the hydroxyl groups of SN that enable the formation of additiona

Name	Ea_H$^+$ (eV)	Ea_H_2 (eV)	Front view (YZ)	Top view (XY)	Information (ID, Name, Pore size, dma, Electron affinity, band gap, Neighbour atom)	Ref
						l proton–transferring pathways, the inorganic–organic membrane displayed enhanced proton conductivity of 48.2 mS/cm and power density of 495 mW/cm2 at 150 °C without humidification when the content of SN is 0.25 wt%. Furthermore, exfoliated SN (E–SN) and sulfonated SN (S–SN), which were fabricated by a liquid–phase exfoliation method and silane condensation, respectively, were embedded in PES–PVP

Name	Ea_H⁺ (eV)	Ea_H_2 (eV)	Front view (YZ)	Top view (XY)	Information (ID, Name, Pore size, dma, Electron affinity, band gap, Neighbour atom)	Ref
						polymer matrix by a simple blending method. Due to the significant contribution from sulfonic groups in S–SN, the membrane with 0.25 wt% S–SN reached the highest proton conductivity of 51.5 mS/cm and peak power density of 546 mW/cm2 at 150 °C, 48% higher than the pristine PES–PVP membranes. Compared to unaltered PES–PVP membrane, SN added hybrid composite membrane demonstr

Name	Ea_H⁺ (eV)	Ea_H_2 (eV)	Front view (YZ)	Top view (XY)	Information (ID, Name, Pore size, dma, Electron affinity, band gap, Neighbour atom)	Ref
						ated excellent durability for the fuel cell at 150 °C. Using a facile method to prepare 2D SN from natural clay minerals, the strategy of exfoliation and functionalization of SN can be potentially used in the production of HT–PEMs.","container-title":"Journal of Energy Chemistry","DOI":"10.1016/j.jechem.2021.04.061","ISSN":"20954956","journalAbbreviation":"Journal of Energy Chemistry","language":"en","license":"https://www.els

Name	Ea_H^{+} (eV)	Ea_H$_2$ (eV)	Front view (YZ)	Top view (XY)	Information (ID, Name, Pore size, dma, Electron affinity, band gap, Neighbour atom)	Ref
						evier.com/tdm/userlicense/1.0/","page":"323-334","source":"DOI.org (Crossref)","title":"High-performance polymer electrolyte membranes incorporated with 2D silica nanosheets in high-temperature proton exchange membrane fuel cells","URL":"https://linkinghub.elsevier.com/retrieve/pii/S2095495621002734","volume":"64","author":[{"family":"Guo","given":"Zunmin"},{"family":"Chen","given":"Jianuo"},{"family":"Byun","given":"Jae Jong"},{

Name	Ea_H$^+$ (eV)	Ea_H_2 (eV)	Front view (YZ)	Top view (XY)	Information (ID, Name, Pore size, dma, Electron affinity, band gap, Neighbour atom)	Ref
						"family":"Cai","given":"Rongsheng"},{"family":"Perez-Page","given":"Maria"},{"family":"Sahoo","given":"Madhumita"},{"family":"Ji","given":"Zhaoqi"},{"family":"Haigh","given":"Sarah J."},{"family":"Holmes","given":"Stuart M."}],"accessed":{"date-parts":[["2024",12,1]]},"issued":{"date-parts":[["2022",1]]}}}],"schema":"https://github.com/citation-style-language/schema/raw/maste
Ge	0.53	2.80	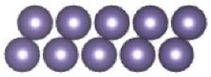	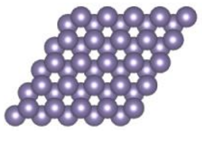	60ef73264dbb56fa22f3da29, Germanene, 0.97 Å^2, 3.03 Å, 1.23eV, 0 eV, Ge	DFT ADDIN ZOTERO_ITEM CSL_CITATION {"citatio

Name	Ea_H⁺ (eV)	Ea_H_2 (eV)	Front view (YZ)	Top view (XY)	Information (ID, Name, Pore size, dma, Electron affinity, band gap, Neighbour atom)	Ref
						es":{"formattedCitation":"\\super 14,18\\nosupersub{}","plainCitation":"14,18","noteIndex":0},"citationItems":[{"id":2017,"uris":["http://zotero.org/users/15250265/items/E4JRYZ7B"],"itemData":{"id":2017,"type":"article-journal","abstract":"We have grown an atom-thin, ordered, two-dimensional multi-phase film in situ through germanium molecular beam epitaxy using a gold (111) surface as a substrate. Its growth is similar to the

Name	Ea_H⁺ (eV)	Ea_H_2 (eV)	Front view (YZ)	Top view (XY)	Information (ID, Name, Pore size, dma, Electron affinity, band gap, Neighbour atom)	Ref
						formation of silicene layers on silver (111) templates. One of the phases, forming large domains, as observed in scanning tunneling microscopy, shows a clear, nearly flat, honeycomb structure. Thanks to thorough synchrotron radiation core-level spectroscopy measurements and advanced density functional theory calculations we can identify it as a √3 × √3

Name	Ea_H$^+$ (eV)	Ea_H_2 (eV)	Front view (YZ)	Top view (XY)	Information (ID, Name, Pore size, dma, Electron affinity, band gap, Neighbour atom)	Ref
						) Au(111) supercell, presenting compelling evidence of the synthesis of the germanium-based cousin of graphene on gold.","container-title":"New Journal of Physics","DOI":"10.1088/1367-2630/16/9/095002","ISSN":"1367-2630","issue":"9","journalAbbreviation":"New J. Phys.","language":"en","note":"publisher: IOP Publishing","page":"095002","source":"Institute of Physics","title":"Germanene: a novel two-dimensional germaniu

Name	Ea_H$^+$ (eV)	Ea_H_2 (eV)	Front view (YZ)	Top view (XY)	Information (ID, Name, Pore size, dma, Electron affinity, band gap, Neighbour atom)	Ref
						m allotrope akin to graphene and silicene","title-short":"Germanene","URL":"https://dx.doi.org/10.1088/1367-2630/16/9/095002","volume":"16","author":[{"family":"Dávila","given":"M. E."},{"family":"Xian","given":"L."},{"family":"Cahangirov","given":"S."},{"family":"Rubio","given":"A."},{"family":"Lay","given":"G. Le"}],"accessed":{"date-parts":[["2024",12,1]]},"issued":{"date-parts":[["2014",9]]}}},{"id":2028,"uris":["http://zotero.org/users/15250265/items/SPDXXW

Name	Ea_H⁺ (eV)	Ea_H₂ (eV)	Front view (YZ)	Top view (XY)	Information (ID, Name, Pore size, dma, Electron affinity, band gap, Neighbour atom)	Ref
						4A"],"itemData":{"id":2028,"type":"article-journal","abstract":"By using ab initio calculations, we predict that a vertical electric field is able to open a band gap in semimetallic single-layer buckled silicene and germanene. The sizes of the band gap in both silicene and germanene increase linearly with the electric field strength. Ab initio quantum transport simulation of a dual-gated silicene field effect transistor confirms

Name	Ea_H+ (eV)	Ea_H₂ (eV)	Front view (YZ)	Top view (XY)	Information (ID, Name, Pore size, dma, Electron affinity, band gap, Neighbour atom)	Ref
						that the vertical electric field opens a transport gap, and a significant switching effect by an applied gate voltage is also observed. Therefore, biased single-layer silicene and germanene can work effectively at room temperature as field effect transistors.","container-title":"Nano Letters","DOI":"10.1021/nl203065e","ISSN":"1530-6984","issue":"1","journalAbbreviation":"Nano Lett.","language":"en-US","note":"publisher:

Name	Ea_H⁺ (eV)	Ea_H_2 (eV)	Front view (YZ)	Top view (XY)	Information (ID, Name, Pore size, dma, Electron affinity, band gap, Neighbour atom)	Ref
						American Chemical Society","page":"113-118","source":"ACS Publications","title":"Tunable Bandgap in Silicene and Germanene","URL":"https://doi.org/10.1021/nl203065e","volume":"12","author":[{"family":"Ni","given":"Zeyuan"},{"family":"Liu","given":"Qihang"},{"family":"Tang","given":"Kechao"},{"family":"Zheng","given":"Jiaxin"},{"family":"Zhou","given":"Jing"},{"family":"Qin","given":"Rui"},{"family":"Gao","given":"Zhengxiang"},{"family":"Yu",

Name	Ea_H⁺ (eV)	Ea_H_2 (eV)	Front view (YZ)	Top view (XY)	Information (ID, Name, Pore size, dma, Electron affinity, band gap, Neighbour atom)	Ref
						"given":"Dapeng"},{"family":"Lu","given":"Jing"}],"accessed":{"date-parts":[["2024",12,1]]},"issued":{"date-parts":[["2012",1,11]]}}}],"schema":"https://github.com/citation-style-language/schema/raw/mast EXPE[19–22] PEM
GeSe	0.58	4.30	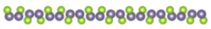	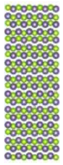	60ef72214dbb56fa22f3d040, Germanium Selenide, 0.91 Å², 3.86 Å, 1.63 eV, 0.35 eV, Se	DFT ADDIN ZOTERO_ITEM CSL_CITATION {"citationID":"N8Duk2AO","properties":{"formattedCitation":"\\super 23,24\\nosupersub{}","plainCitation":"23,24","noteIndex":0},"citationItems":[{"id":2035,"uris":["http://zotero.org/user

Name	Ea_H+ (eV)	Ea_H_2 (eV)	Front view (YZ)	Top view (XY)	Information (ID, Name, Pore size, dma, Electron affinity, band gap, Neighbour atom)	Ref
						QSF"],"itemData":{"id":2035,"type":"article-journal","abstract":"GeSe has emerged as an appealing photovoltaic material due to the desirable electronic and optical properties as well as being an earth-abundant constituent element. We systematically explore for the first time the contact properties of bilayer GeSe with commonly used back electrode metals in detail, such as the geometric features, electronic

Name	Ea_H^+ (eV)	Ea_H_2 (eV)	Front view (YZ)	Top view (XY)	Information (ID, Name, Pore size, dma, Electron affinity, band gap, Neighbour atom)	Ref
						properties, Schottky barrier, tunneling barrier, and band alignment. Our results reveal that the metals investigated, especially Au, Pt, and Ni, show great potential in forming favorable contacts with GeSe due to a low Schottky barrier and tunneling barrier. More importantly, we find that when a SnS monolayer is superimposed with GeSe layers, the combined system can be used as an effective solar cell material with type-II

Name	Ea_H⁺ (eV)	Ea_H_2 (eV)	Front view (YZ)	Top view (XY)	Information (ID, Name, Pore size, dma, Electron affinity, band gap, Neighbour atom)	Ref
						heterostructure alignment. The power conversion efficiency is predicted to be as high as ~18%, which is comparable or even higher than that of previously reported solar cells. Our results not only provide microscopic insights into the characteristics between layered GeSe and metals, but also pave the way for further experimental improvements of GeSe thin-film solar cells.","container-title":"Journal of Materials Chemistr

Name	Ea_H⁺ (eV)	Ea_H₂ (eV)	Front view (YZ)	Top view (XY)	Information (ID, Name, Pore size, dma, Electron affinity, band gap, Neighbour atom)	Ref
						y A","DOI":"10.1039/C8TA00129D","ISSN":"2050-7496","issue":"12","journalAbbreviation":"J. Mater. Chem. A","language":"en","note":"publisher: The Royal Society of Chemistry","page":"5032-5039","source":"pubs-rsc-org.khalifa.idm.oclc.org","title":"Two-dimensional GeSe for high performance thin-film solar cells","URL":"https://pubs.rsc.org/en/content/articlelanding/2018/ta/c8ta00129d","volume":"6","author":[{"family":"Lv","given":"Xingshuai"},{"family

Name	Ea_H^+ (eV)	Ea_H_2 (eV)	Front view (YZ)	Top view (XY)	Information (ID, Name, Pore size, dma, Electron affinity, band gap, Neighbour atom)	Ref
						":"Wei","given":"Wei"},{"family":"Mu","given":"Cong"},{"family":"Huang","given":"Baibiao"},{"family":"Dai","given":"Ying"}],"accessed":{"date-parts":[["2024",12,1]]},"issued":{"date-parts":[["2018",3,20]]}}},{"id":2037,"uris":["http://zotero.org/users/15250265/items/IQD9WK9W"],"itemData":{"id":2037,"type":"article-journal","abstract":"We have used highly accurate quantum Monte Carlo methods to determine the chemical structure and electroni

Name	Ea_H$^+$ (eV)	Ea_H_2 (eV)	Front view (YZ)	Top view (XY)	Information (ID, Name, Pore size, dma, Electron affinity, band gap, Neighbour atom)	Ref
						c band gaps of monolayer GeSe. Two-dimensional (2D) monolayer GeSe has received a great deal of attention due to its unique thermoelectric, electronic, and optoelectronic properties with a wide range of potential applications. Density functional theory (DFT) methods have usually been applied to obtain optical and structural properties of bulk and 2D GeSe. For the monolayer, DFT typically yields a larger band-gap energy than for bulk

Name	Ea_H^+ (eV)	Ea_H_2 (eV)	Front view (YZ)	Top view (XY)	Information (ID, Name, Pore size, dma, Electron affinity, band gap, Neighbour atom)	Ref
						GeSe but cannot conclusively determine if the monolayer has a direct or indirect gap. Moreover, the DFT-optimized lattice parameters and atomic coordinates for monolayer GeSe depend strongly on the choice of approximation for the exchange-correlation functional, which makes the ideal structure—and its electronic properties—unclear. In order to obtain accurate lattice parameters and atomic coordinates for the monolayer, we

Name	Ea_H+ (eV)	Ea_H2 (eV)	Front view (YZ)	Top view (XY)	Information (ID, Name, Pore size, dma, Electron affinity, band gap, Neighbour atom)	Ref
						use a surrogate Hessian-based parallel line search within diffusion Monte Carlo to fully optimize the GeSe monolayer structure. The DMC-optimized structure is different from those obtained using DFT, as are calculated band gaps. The potential energy surface has a shallow minimum at the optimal structure. This, combined with the sensitivity of the electronic structure to strain, suggests that the optical propertie

Name	Ea_H+ (eV)	Ea_H2 (eV)	Front view (YZ)	Top view (XY)	Information (ID, Name, Pore size, dma, Electron affinity, band gap, Neighbour atom)	Ref
						s of monolayer GeSe are highly tunable by strain.","container-title":"Physical Review Materials","DOI":"10.1103/PhysRevMaterials.5.024002","issue":"2","journalAbbreviation":"Phys. Rev. Mater.","note":"publisher: American Physical Society","page":"024002","source":"APS","title":"Optimized structure and electronic band gap of monolayer GeSe from quantum Monte Carlo methods","URL":"https://link.aps.org/doi/10.1103/PhysRevMate

Name	Ea_H⁺ (eV)	Ea_H₂ (eV)	Front view (YZ)	Top view (XY)	Information (ID, Name, Pore size, dma, Electron affinity, band gap, Neighbour atom)	Ref
						rials.5.024002","volume":"5","author":[{"family":"Shin","given":"Hyeondeok"},{"family":"Krogel","given":"Jaron T."},{"family":"Gasperich","given":"Kevin"},{"family":"Kent","given":"Paul R. C."},{"family":"Benali","given":"Anouar"},{"family":"Heinonen","given":"Olle"}],"accessed":{"date-parts":[["2024",12,1]]},"issued":{"date-parts":[["2021",2,10]]}}}],"schema":"https://github.com/citation-style-language/schema/raw/mast E X P ADDIN ZOTER

Name	Ea_H⁺ (eV)	Ea_H_2 (eV)	Front view (YZ)	Top view (XY)	Information (ID, Name, Pore size, dma, Electron affinity, band gap, Neighbour atom)	Ref
						{"citationID":"J2KMby6r","properties":{"formattedCitation":"\\super 25\\nosupersub{}","plainCitation":"25","noteIndex":0},"citationItems":[{"id":2033,"uris":["http://zotero.org/users/15250265/items/V4NI4CD6"],"itemData":{"id":2033,"type":"article-journal","abstract":"We demonstrate the synthesis of layered anisotropic semiconductor GeSe and GeSe2 nanomaterials through low temperature (~400 °C) and atmospheric pressure chemical vapor

Name	Ea_H⁺ (eV)	Ea_H_2 (eV)	Front view (YZ)	Top view (XY)	Information (ID, Name, Pore size, dma, Electron affinity, band gap, Neighbour atom)	Ref
						deposition using halide based precursors. Results show that GeI2 and H2Se precursors successfully react in the gas-phase and nucleate on a variety of target substrates including sapphire, Ge, GaAs, or HOPG. Layer-by-layer growth takes place after nucleation to form layered anisotropic materials. Detailed SEM, EDS, XRD, and Raman spectroscopy measurements together with systematic CVD studies

Name	Ea_H+ (eV)	Ea_H2 (eV)	Front view (YZ)	Top view (XY)	Information (ID, Name, Pore size, dma, Electron affinity, band gap, Neighbour atom)	Ref
						reveal that the substrate temperature, selenium partial pressure, and the substrate type ultimately dictate the resulting stoichiometry and phase of these materials. Results from this work introduce the phase control of Ge and Se based nanomaterials (GeSe and GeSe2) using halide based CVD precursors at ATM pressures and low temperatures. Overall findings also extend our fundamental understanding of their growth

Name	Ea_H$^+$ (eV)	Ea_H_2 (eV)	Front view (YZ)	Top view (XY)	Information (ID, Name, Pore size, dma, Electron affinity, band gap, Neighbour atom)	Ref
						by making the first attempt to correlate growth parameters to resulting competing phases of Ge–Se based materials.","container-title":"RSC Advances","DOI":"10.1039/D0RA07539F","ISSN":"2046-2069","issue":"63","journalAbbreviation":"RSC Adv.","language":"en","note":"publisher: The Royal Society of Chemistry","page":"38227-38232","source":"pubs.rsc.org","title":"The synthesis of competing phase GeSe and GeSe2 2D layered

Name	Ea_H⁺ (eV)	Ea_H_2 (eV)	Front view (YZ)	Top view (XY)	Information (ID, Name, Pore size, dma, Electron affinity, band gap, Neighbour atom)	Ref
						materials","URL":"https://pubs.rsc.org/en/content/articlelanding/2020/ra/d0ra07539f","volume":"10","author":[{"family":"Yumigeta","given":"Kentaro"},{"family":"Brayfield","given":"Cassondra"},{"family":"Cai","given":"Hui"},{"family":"Hajra","given":"Debarati"},{"family":"Blei","given":"Mark"},{"family":"Yang","given":"Sijie"},{"family":"Shen","given":"Yuxia"},{"family":"Tongay","given":"S."}],"accessed":{"date-parts":[["2024",12,1]]},"issued":{"date-parts":[["2020",10,15]]}}}],"schema

Name	Ea_H⁺ (eV)	Ea_H_2 (eV)	Front view (YZ)	Top view (XY)	Information (ID, Name, Pore size, dma, Electron affinity, band gap, Neighbour atom)	Ref
						":"https://github.com/citation-style-language/schema/raw/mast PEM
I3N	0.91	3.97	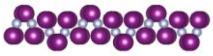	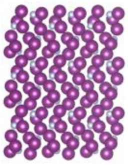	60ef719c3b482494c462f839, Nitrogen triiodide, 2.52 Å², 3.05 Å, 1.94 eV, 0.64 eV, N	DFT EXP ADDIN ZOTERO_ITEM CSL_CITATION {"citationID":"YFV7Ak5A","properties":{"formattedCitation":"\\super 26,27\\nosupersub{}","plainCitation":"26,27","noteIndex":0},"citationItems":[{"id":2042,"uris":["http://zotero.org/users/15250265/items/K2JHTBN8"],"itemData":{"id":2042,"type":"chapter","abstract":"Although new investigations on inorganic nitrogen compounds of

Name	Ea_H$^+$ (eV)	Ea_H_2 (eV)	Front view (YZ)	Top view (XY)	Information (ID, Name, Pore size, dma, Electron affinity, band gap, Neighbour atom)	Ref
						iodine have been reviewed comparatively recently, it seems appropriate to discuss the chemistry and structures of nitrogen triiodide, -chloride, and -bromide once more since highly interesting, and in part, surprising results have emerged in the course of the past 2 years. Liquid ammonia (ca. 1 mg/ml at - 76°C) and no dissolution was observed in the experiments, an exchange mechanism other than that involving solution seems likely. To

Name	Ea_H^+ (eV)	Ea_H_2 (eV)	Front view (YZ)	Top view (XY)	Information (ID, Name, Pore size, dma, Electron affinity, band gap, Neighbour atom)	Ref
						clarify this point, exchange experiments between solid nitrogen triiodide-1 -ammonia and gaseous ammonia were made and followed by IR spectroscopy. The relationship between the triammine and the pentammine is also brought out by the observation that the red pentammine is converted into the green triammine above -76°C either in liquid ammonia or in the mixed solvent referred to above. The green compound is also

Name	Ea_H^{+} (eV)	Ea_H_2 (eV)	Front view (YZ)	Top view (XY)	Information (ID, Name, Pore size, dma, Electron affinity, band gap, Neighbour atom)	Ref
						transformed into the red one in the mixed solvent at -75°C to -86°C. It is believed that the second red compound is monoiodamine- 1 – ammonia.","container-title":"Advances in Inorganic Chemistry and Radiochemistry","note":"DOI: 10.1016/S0065-2792(08)60070-9","page":"1-63","publisher":"Academic Press","source":"ScienceDirect","title":"Recent Chemistry and Structure Investigation of Nitrogen Triiodide, Tribromi

Name	Ea_H$^+$ (eV)	Ea_H$_2$ (eV)	Front view (YZ)	Top view (XY)	Information (ID, Name, Pore size, dma, Electron affinity, band gap, Neighbour atom)	Ref
						de, Trichloride, and Related Compounds","URL":"https://www.sciencedirect.com/science/article/pii/S0065279208600709","volume":"19","author":[{"family":"Jander","given":"Jochen"}],"editor":[{"family":"Emeléus","given":"H. J."},{"family":"Sharpe","given":"A. G."}],"accessed":{"date-parts":[["2024",12,1]]},"issued":{"date-parts":[["1976",1,1]]}}},{"id":2041,"uris":["http://zotero.org/users/15250265/items/ZV27PHXY"],"itemData":{"id":2041,"type":"article-journal",

Name	Ea_H⁺ (eV)	Ea_H_2 (eV)	Front view (YZ)	Top view (XY)	Information (ID, Name, Pore size, dma, Electron affinity, band gap, Neighbour atom)	Ref
						"abstract":"The present work is dedicated to deepen the causes of the physicochemical instability of NI3. As a support study, a theoretical investigation is performed. The quantum chemical calculations were performed by using SE(PM6), HF/6-31G* and DFT/M06-2X/6-311G** approach. The calculated gas phase formation enthalpy to NH3.NI3 is 91.75 kJmol−1. The sublimation enthalpy for the adduct is calculated as

Name	Ea_H$^+$ (eV)	Ea_H_2 (eV)	Front view (YZ)	Top view (XY)	Information (ID, Name, Pore size, dma, Electron affinity, band gap, Neighbour atom)	Ref
						237.75 kJmol−1. By structure calculations, it is shown that the stabilization of NI3 in the NH3.NI3 adduct is consequence of the I-N-I angle increase. The enthalpy for the reaction NH3.NI3 (s) → NH3(g) + NI3 (s) is calculated as 46 kJmol−1. Pure NH3:NI3 detonated only three minutes after dried at room conditions (humidity = 65%, temperature = 39 ºC). On the other hand, the NH3:NI3 polyvinyl chloride resin, polyvinyl acetate resin or

Name	Ea_H+ (eV)	Ea_H_2 (eV)	Front view (YZ)	Top view (XY)	Information (ID, Name, Pore size, dma, Electron affinity, band gap, Neighbour atom)	Ref
						polysiloxane resin "entrapped" samples, do not detonate after 30 h. By using the empirical equation Is = 17.562 η + 125.551, where Is is the specific impulse (s) and η is the absolute chemical hardness (eV), the specific impulse for NH3.NI3 can be calculated as 185.52 s (HF/6-31G* data) and 163.75 s (DFT/M06-2X/6-311G** data). To NI3the Is calculated values are 201.68s and 168.75 s, respectively. The decrease in the specific impulse values

Name	Ea_H+ (eV)	Ea_H2 (eV)	Front view (YZ)	Top view (XY)	Information (ID, Name, Pore size, dma, Electron affinity, band gap, Neighbour atom)	Ref
						from NI3 to NI3:NH3 is explained as consequence of the increase in the polarizability of the entire system.","container-title":"Journal of Molecular Structure","DOI":"10.1016/j.molstruc.2021.130075","ISSN":"0022-2860","journalAbbreviation":"Journal of Molecular Structure","page":"130075","source":"ScienceDirect","title":"The structure, thermodynamic instability and energetics of NI3, its specific impulse and a strategy

Name	Ea_H⁺ (eV)	Ea_H_2 (eV)	Front view (YZ)	Top view (XY)	Information (ID, Name, Pore size, dma, Electron affinity, band gap, Neighbour atom)	Ref
						for its stabilization","URL":"https://www.sciencedirect.com/science/article/pii/S0022286021002064","volume":"1232","author":[{"family":"Marinho","given":"George S."},{"family":"Farias","given":"Robson F.","non-dropping-particle":"de"}],"accessed":{"date-parts":[["2024",12,1]]},"issued":{"date-parts":[["2021",5,15]]}}}],"schema":"https://github.com/citation-style-language/schema/raw/maste PEM
TeCl	0.92	5.00	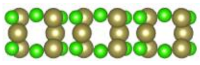	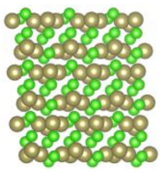	60ef71e3c403a99258a82e29, Tellurium Monochloride, 0.1.99Å^2, 2.69Å, 2.79eV, 0.76 eV, Te	DFT E X P ADDIN ZOTERO_ITEM

Name	Ea_H⁺ (eV)	Ea_H_2 (eV)	Front view (YZ)	Top view (XY)	Information (ID, Name, Pore size, dma, Electron affinity, band gap, Neighbour atom)	Ref
						nID":"wdzEmHBJ","properties":{"formattedCitation":"\\super 28\\nosupersub{}","plainCitation":"28","noteIndex":0},"citationItems":[{"id":2044,"uris":["http://zotero.org/users/15250265/items/H9R6LVND"],"itemData":{"id":2044,"type":"article-journal","container-title":"Journal of Molecular Spectroscopy","DOI":"10.1016/0022-2852(71)90301-8","ISSN":"00222852","issue":"2","journalAbbreviation":"Journal of Molecular Spectroscopy","language":"en","lice

Name	Ea_H⁺ (eV)	Ea_H_2 (eV)	Front view (YZ)	Top view (XY)	Information (ID, Name, Pore size, dma, Electron affinity, band gap, Neighbour atom)	Ref
						nse":"https://www.elsevier.com/tdm/userlicense/1.0/","page":"314-320","source":"DOI.org (Crossref)","title":"Ultraviolet absorption spectra of tellurium monochloride and tellurium monobromide","URL":"https://linkinghub.elsevier.com/retrieve/pii/0022285271903018","volume":"37","author":[{"family":"Oldershaw","given":"G.A."},{"family":"Robinson","given":"K."}],"accessed":{"date-parts":[["2024",12,1]]},"issued":{"date-parts":[["1971",2]]}}}],"schema":"https://githu

Name	Ea_H^{+} (eV)	Ea_H$_2$ (eV)	Front view (YZ)	Top view (XY)	Information (ID, Name, Pore size, dma, Electron affinity, band gap, Neighbour atom)	Ref
						b.com/citation-style-language/schema/raw/maste PErM
SiS	0.99	4.89	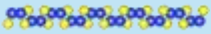	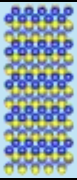	60ef720bc403a99258a82fc3, Silicon Monosulfide, 1.09 Å^2, 2.23 Å, 1.73 eV, 0 eV, Si	DFT[29–31] EXP PEM
SiSe	1.03	1.94	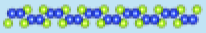	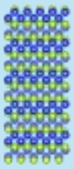	60ef71f73b482494c462faeb, Silicon Monoselenide, 0.51Å^2, 2.23 Å, 1.73eV, 0 eV, Se	DFT[32–34] EXP PEM
SN	1.06	3.20	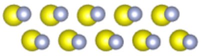	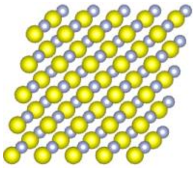	60ef720b4dbb56fa22f3cf6b, Sulfur Mononitride, 1.45Å^2, 3.14Å, 0.34eV, 0 eV, S	DFT EXP ADDIN ZOTERO_ITEM CSL_CITATION {"citationID":"Qc0ZWdaz","properties":{"formattedCitation":"\\super 35\\nosupersub{}","plainCitation":"35","noteIndex":0},"citationItems":[{"id":2057,"uris":["http://zotero.org/users/15250265/items/DXX3EGBD"],"itemData":{"id":2057,"t

Name	Ea_H+ (eV)	Ea_H2 (eV)	Front view (YZ)	Top view (XY)	Information (ID, Name, Pore size, dma, Electron affinity, band gap, Neighbour atom)	Ref
						"abstract":"The explosive properties of tetrasulfur tetranitride, S4N4, are reviewed and analysed. S4N4 is an explosive compound with an impact sensitivity comparable to pentaerythritol tetranitrate (PETN) (Ei=4 J) but a friction sensitivity equal or even lower than lead azide (F=0.1–1 N). S4N4 has primary explosive properties. It has a working capacity greater than silver azide, AgN3, and is capable to initiate TNT.

Name	Ea_H⁺ (eV)	Ea_H_2 (eV)	Front view (YZ)	Top view (XY)	Information (ID, Name, Pore size, dma, Electron affinity, band gap, Neighbour atom)	Ref
						S4N4 shows an unusual non-linear density/detonation velocity relationship which is due to different stability regimes of sulfur species present in the Chapman Jouguet zone. S4N4 is stable, non-ageing, non-toxic, insoluble in water and hence an interesting candidate for future lead tricinate replacement.","container-title":"Zeitschrift für anorganische und allgemeine Chemie","DOI":"10.1002/zaac.202000406","ISSN":"1521-3749","is

Name	Ea_H+ (eV)	Ea_H_2 (eV)	Front view (YZ)	Top view (XY)	Information (ID, Name, Pore size, dma, Electron affinity, band gap, Neighbour atom)	Ref
						sue":"4","language":"en","license":"© 2020 Wiley-VCH GmbH","note":"_eprint: https://onlinelibrary.wiley.com/doi/pdf/10.1002/zaac.202000406","page":"192-199","source":"Wiley Online Library","title":"Analysis of the Explosive Properties of Tetrasulfur Tetranitride, S4N4","URL":"https://onlinelibrary.wiley.com/doi/abs/10.1002/zaac.202000406","volume":"647","author":[{"family":"Koch","given":"Ernst-Christian"},{"family":"Sućeska","given":"Mu

Name	Ea_H⁺ (eV)	Ea_H₂ (eV)	Front view (YZ)	Top view (XY)	Information (ID, Name, Pore size, dma, Electron affinity, band gap, Neighbour atom)	Ref
						hamed"}],"accessed":{"date-parts":[["2024",12,1]]},"issued":{"date-parts":[["2021"]]}}}],"schema":"https://github.com/citation-style-language/schema/raw/maste PEM
Si_3Br_8	1.10	15	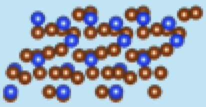	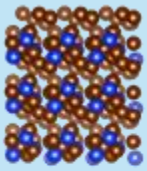	60ef716682379218cee7a5e3, Trisilicon Octabromide, 1.40Å^2, 3.14Å, 2.83eV, 0.66 eV, Br	DFT EXP PEM
F_2	1.14	8.17	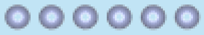	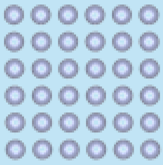	60ef73284dbb56fa22f3da3a, Fluorine, 2.76Å^2, 1.98Å, 3.40eV, 0 eV, F	DFT EXP PEM
CSe	1.38	9.76	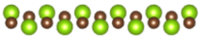	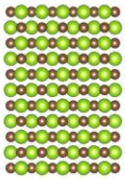	60ef7176c403a99258a829b3, Carbon Monoselenide, 0.49Å^2, 2.25Å, 1.64eV, 0.31eV, Se	DFT ADDIN ZOTERO_ITEM CSL_CITATION {"citationID":"C26bEucU","properties":{"formattedCitation":"\\super 36,37\\nosupersub{}","plainCitation":"36,37","noteInd

Name	Ea_H^+ (eV)	Ea_H_2 (eV)	Front view (YZ)	Top view (XY)	Information (ID, Name, Pore size, dma, Electron affinity, band gap, Neighbour atom)	Ref
						":2059,"uris":["http://zotero.org/users/15250265/items/HESH8722"],"itemData":{"id":2059,"type":"article-journal","abstract":"A new generation of two-dimensional (2D) material has captivated significant attention in the energy conversion field owing to their promising optoelectronics and thermoelectric applications. The present work involves the systematic investigation of fundamental properties of single-layered 2D

Name	Ea_H+ (eV)	Ea_H2 (eV)	Front view (YZ)	Top view (XY)	Information (ID, Name, Pore size, dma, Electron affinity, band gap, Neighbour atom)	Ref
						carbon-based monochalcogenides (CS, CSe, CTe) with planar, buckled and puckered geometry within the framework of density functional theory (DFT). The structural and lattice dynamics analysis disclose that puckered and buckled configurations are energetically and dynamically stable whereas planar structures depict instability. The anisotropic group velocity of longitudinal acoustic (LA) and transverse acoustic

Name	Ea_H^+ (eV)	Ea_H_2 (eV)	Front view (YZ)	Top view (XY)	Information (ID, Name, Pore size, dma, Electron affinity, band gap, Neighbour atom)	Ref
						(TA) phonon modes in puckered systems may render the characteristics thermal transport properties. Additionally, for the first time, we scrutinized the thermoelectric and optical properties of these materials. At room temperature, the electron carrier mobilities are 174.698 and 160.830 m2V-1s-1 of puckered and buckled CS systems, respectively are highest among all structures. The computed Seebeck coefficient,

Name	Ea_H+ (eV)	Ea_H2 (eV)	Front view (YZ)	Top view (XY)	Information (ID, Name, Pore size, dma, Electron affinity, band gap, Neighbour atom)	Ref
						electrical conductivity and power factor manifests the high thermoelectric transport properties of puckered CS material. Further, the calculated solar parameters demonstrate an exceptionally high-power conversion efficiency of 19.61 % for puckered CTe. Present work indicates that puckered phase of CS and CTe show their potential for the heat and solar energy harvesting devices, respectively.","container-

Name	Ea_H+ (eV)	Ea_H2 (eV)	Front view (YZ)	Top view (XY)	Information (ID, Name, Pore size, dma, Electron affinity, band gap, Neighbour atom)	Ref
						title":"Applied Surface Science","DOI":"10.1016/j.apsusc.2022.155121","ISSN":"0169-4332","journalAbbreviation":"Applied Surface Science","page":"155121","source":"ScienceDirect","title":"Carbon-based monochalcogenides for efficient solar and heat energy harvesting","URL":"https://www.sciencedirect.com/science/article/pii/S0169433222026496","volume":"608","author":[{"family":"Bhojani","given":"Amit K."},{"family":"Kagdada","given":"Hardik L."},{"fa

Name	Ea_H+ (eV)	Ea_H_2 (eV)	Front view (YZ)	Top view (XY)	Information (ID, Name, Pore size, dma, Electron affinity, band gap, Neighbour atom)	Ref
						mily":"Ahuja","given":"Rajeev"},{"family":"Singh","given":"Dheeraj K."}],"accessed":{"date-parts":[["2024",12,1]]},"issued":{"date-parts":[["2023",1,15]]}}},{"id":2065,"uris":["http://zotero.org/users/15250265/items/KMGCRAL2"],"itemData":{"id":2065,"type":"article-journal","abstract":"Beilstein Journal of Nanotechnology","container-title":"Beilstein Journal of Nanotechnology","DOI":"10.3762/bjnano.8.135","ISSN":"2190-4286","issue":"1",

Name	Ea_H⁺ (eV)	Ea_H_2 (eV)	Front view (YZ)	Top view (XY)	Information (ID, Name, Pore size, dma, Electron affinity, band gap, Neighbour atom)	Ref
						"journal Abbreviation":"Beilstein J. Nanotechnol.","language":"en","license":"© 2017 Rocca et al.; licensee Beilstein-Institut.","note":"publisher: Beilstein-Institut","page":"1338-1344","source":"www.beilstein-journals.org","title":"Two-dimensional silicon and carbon monochalcogenides with the structure of phosphorene","URL":"https://www.beilstein-journals.org/bjnano/articles/8/135","volume":"8","author":[{"family":"Rocca","given":"Da

Name	Ea_H⁺ (eV)	Ea_H_2 (eV)	Front view (YZ)	Top view (XY)	Information (ID, Name, Pore size, dma, Electron affinity, band gap, Neighbour atom)	Ref
						rio"},{"family":"Abboud","given":"Ali"},{"family":"Vaitheeswaran","given":"Ganapathy"},{"family":"Lebègue","given":"Sébastien"}],"accessed":{"date-parts":[["2024",12,1]]},"issued":{"date-parts":[["2017",6,29]]}}}],"schema":"https://github.com/citation-style-language/schema/raw/mast ADDIN ZOTERO_ITEM CSL_CITATION {"citationID":"SVRO96Ap","properties":{"formattedCitation":"\\super 38,39\\nosupersub{}","plainCitation":"38,39"

Name	Ea_H^+ (eV)	Ea_H_2 (eV)	Front view (YZ)	Top view (XY)	Information (ID, Name, Pore size, dma, Electron affinity, band gap, Neighbour atom)	Ref
						ms":[{"id":2060,"uris":["http://zotero.org/users/15250265/items/PGH4W3ID"],"itemData":{"id":2060,"type":"article-journal","abstract":"We perform systematic investigation on the geometric, energetic, and electronic properties of group IV-VI binary monolayers (*XY*), which are the counterparts of phosphorene, by employing density functional theory based electronic structure calculations. For this purpose, we choose the

Name	Ea_H$^+$ (eV)	Ea_H_2 (eV)	Front view (YZ)	Top view (XY)	Information (ID, Name, Pore size, dma, Electron affinity, band gap, Neighbour atom)	Ref
						binary systems XY consisting of equal numbers of group IV (X = C, Si, Ge, Sn) and group VI elements (Y = O, S, Se, Te) in three geometrical configurations, the puckered, buckled and planar structures. The results of binding energy calculations show that all the binary systems studied are energetically stable. It is observed that, the puckered structure, similar to that of phosphorene, is the energetically most stable geometric

Name	Ea_H^+ (eV)	Ea_H_2 (eV)	Front view (YZ)	Top view (XY)	Information (ID, Name, Pore size, dma, Electron affinity, band gap, Neighbour atom)	Ref
						configuration. Moreover, the binding energies of buckled configuration are very close to those of the puckered configuration. Our results of electronic band structure predict that puckered SiO and CSe are direct band semiconductors with gaps of 1.449 and 0.905 eV, respectively. Band structure of CSe closely resembles that of phosphorene. Remaining group IV-VI binary monolayers in the puckered configuration and all the buckled monolayers are

Name	Ea_H$^+$ (eV)	Ea_H_2 (eV)	Front view (YZ)	Top view (XY)	Information (ID, Name, Pore size, dma, Electron affinity, band gap, Neighbour atom)	Ref
						also semiconductors, but with indirect band gaps. Importantly, we find that the difference between indirect and direct band gaps is very small for many puckered monolayers. Thus there is a possibility of making these systems undergo transition from indirect to direct band gap semiconducting state by a suitable external influence. Indeed, we show in the present work that seven binary monolayers, namely, SnS, SiSe, GeSe,

Name	Ea_H+ (eV)	Ea_H2 (eV)	Front view (YZ)	Top view (XY)	Information (ID, Name, Pore size, dma, Electron affinity, band gap, Neighbour atom)	Ref
						SnSe, SiTe, GeTe, and SnTe become direct band gap semiconductors when they are subjected to a small mechanical strain (≤3%). This makes nine out of sixteen binary monolayers studied in the present work direct band gap semiconductors. Thus there is a possibility of utilizing these binary counterparts of phosphorene in future light-emitting diodes and solar cells.","container-title":"Physical Review B","DOI

Name	Ea_H$^+$ (eV)	Ea_H_2 (eV)	Front view (YZ)	Top view (XY)	Information (ID, Name, Pore size, dma, Electron affinity, band gap, Neighbour atom)	Ref
						":"10.1103/PhysRevB.93.125428","issue":"12","journalAbbreviation":"Phys. Rev. B","note":"publisher: American Physical Society","page":"125428","source":"APS","title":"Direct band gaps in group IV-VI monolayer materials: Binary counterparts of phosphorene","title-short":"Direct band gaps in group IV-VI monolayer materials","URL":"https://link.aps.org/doi/10.1103/PhysRevB.93.125428","volume":"93","author":[{"family":"Kamal","given"

Name	Ea_H⁺ (eV)	Ea_H₂ (eV)	Front view (YZ)	Top view (XY)	Information (ID, Name, Pore size, dma, Electron affinity, band gap, Neighbour atom)	Ref
						:"C."},{"family":"Chakrabarti","given":"Aparna"},{"family":"Ezawa","given":"Motohiko"}],"accessed":{"date-parts":[["2024",12,1]]},"issued":{"date-parts":[["2016",3,23]]}}},{"id":2067,"uris":["http://zotero.org/users/15250265/items/I86VV5KE"],"itemData":{"id":2067,"type":"article-journal","abstract":"An improved source of the CSe band systemin emission has been discovered which has enabled a partial rotational analysis of seven bands to be made. The value

Name	Ea_H+ (eV)	Ea_H_2 (eV)	Front view (YZ)	Top view (XY)	Information (ID, Name, Pore size, dma, Electron affinity, band gap, Neighbour atom)	Ref
						derived for re\" is 1.67 Å. No evidence against the view that the main systemis 1Π-1Σ, in which the 1Π state is greatly perturbed, has been obtained. ","container-title":"Proceedings of the Physical Society. Section A","DOI":"10.1088/0370-1298/66/9/309","ISSN":"0370-1298","issue":"9","journalAbbreviation":"Proc. Phys. Soc. A","language":"en","page":"836","source":"Institute of Physics","title":"The Ultra-Violet Band Spectrum of Carbon

Name	Ea_H$^+$ (eV)	Ea_H_2 (eV)	Front view (YZ)	Top view (XY)	Information (ID, Name, Pore size, dma, Electron affinity, band gap, Neighbour atom)	Ref
						Monoselenide","URL":"https://dx.doi.org/10.1088/0370-1298/66/9/309","volume":"66","author":[{"family":"Laird","given":"R.K."},{"family":"Barrow","given":"R.F."}],"accessed":{"date-parts":[["2024",12,1]]},"issued":{"date-parts":[["1953",9]]}}}],"schema":"https://github.com/citation-style-language/schema/raw/maste
SeI	1.39	12.35	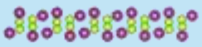	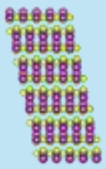	60ef71c33b482494c462f97b, Selenium Monoiodide, 3.58Å^2, 3.29Å, 2.54eV, 0.65 eV, Se	PEMDFT ADDIN ZOTERO_ITEM CSL_CITATION {"citationID":"eqSZwMtC","properties":{"formattedCitation":"

Name	Ea_H⁺ (eV)	Ea_H_2 (eV)	Front view (YZ)	Top view (XY)	Information (ID, Name, Pore size, dma, Electron affinity, band gap, Neighbour atom)	Ref
						","plainCitation":"40","noteIndex":0},"citationItems":[{"id":2073,"uris":["http://zotero.org/users/15250265/items/6SVIFBSG"],"itemData":{"id":2073,"type":"article-journal","abstract":"A new species, selenium monoiodide (SeI), was investigated for the first time at a high level of theoretical approach, SA-CASSCF/MRCI. The overall picture of all doublet and quartet (K + S) states correlating with the three lowest dissociation channels and the

Name	Ea_H^+ (eV)	Ea_H_2 (eV)	Front view (YZ)	Top view (XY)	Information (ID, Name, Pore size, dma, Electron affinity, band gap, Neighbour atom)	Ref
						associated X states provide reliable results to help understand the lack of experimental data on its transitions and to plan the investigation and determination of spectroscopic parameters. Transition probabilities were computed for the transitions X2 – X1, A1 – X1, A2 – X1, and A2 – X2, originated from the spin-forbidden 1 4R - X 2P system, and the corresponding radiative lifetimes evaluated.","container-title":"Chemical Physics Letters","DOI":"1

Name	Ea_H+ (eV)	Ea_H_2 (eV)	Front view (YZ)	Top view (XY)	Information (ID, Name, Pore size, dma, Electron affinity, band gap, Neighbour atom)	Ref
						0.1016/j.cplett.2017.01.013","ISSN":"00092614","journalAbbreviation":"Chemical Physics Letters","language":"en","page":"78-83","source":"DOI.org (Crossref)","title":"Electronic states and spectroscopic parameters of selenium monoiodide, SeI: A theoretical contribution","title-short":"Electronic states and spectroscopic parameters of selenium monoiodide, SeI","URL":"https://linkinghub.elsevier.com/retrieve/pii/S0009261417300258","v

Name	Ea_H^+ (eV)	Ea_H_2 (eV)	Front view (YZ)	Top view (XY)	Information (ID, Name, Pore size, dma, Electron affinity, band gap, Neighbour atom)	Ref
						olume":"671","author":[{"family":"Belinassi","given":"Antonio Ricardo"},{"family":"Alves","given":"Tiago Vinicius"},{"family":"Ornellas","given":"Fernando R."}],"accessed":{"date-parts":[["2024",12,1]]},"issued":{"date-parts":[["2017",3]]}}}],"schema":"https://github.com/citation-style-language/schema/raw/maste EXP PEM
HC	0.53	12.50	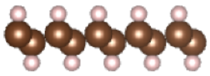	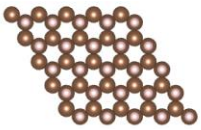	60ef72433b482494c462fd5f, Hydrogenated Graphene (graphane), 1.78Å^2, 2.18Å, 1.01eV, 3.49eV, H	DFT ADDIN ZOTERO_ITEM CSL_CITATION {"citationID":"wTWEouuC","properties":{"formatte

Name	Ea_H⁺ (eV)	Ea_H_2 (eV)	Front view (YZ)	Top view (XY)	Information (ID, Name, Pore size, dma, Electron affinity, band gap, Neighbour atom)	Ref
						persub{} ","plainCitation":"41","noteIndex":0},"citationItems":[{"id":2081,"uris":["http://zotero.org/users/15250265/items/IISPKMHJ"],"itemData":{"id":2081,"type":"article-journal","abstract":"In order to explain the disagreement between present theoretical and experimental investigations on the stability of hydrogenated graphene, we have systematically studied hydrogenated graphene with different configurations from the considera

Name	Ea_H^+ (eV)	Ea_H_2 (eV)	Front view (YZ)	Top view (XY)	Information (ID, Name, Pore size, dma, Electron affinity, band gap, Neighbour atom)	Ref
						tion of single-side and double-side adsorption using first-principles calculations. Both binding energy and formation energy are calculated to characterize the stability of the system. It is found that single-side hydrogenated graphene is always unstable. However, for double-side hydrogenation, some configurations are stable due to the increased carbon–carbon sp3 hybridization compared to

Name	Ea_H$^+$ (eV)	Ea_H_2 (eV)	Front view (YZ)	Top view (XY)	Information (ID, Name, Pore size, dma, Electron affinity, band gap, Neighbour atom)	Ref
						single-side hydrogenation. Furthermore, it is found that the systemis energetically favorable when an equal number of hydrogen atoms are adsorbed on each side of the graphene.","container-title":"RSC Advances","DOI":"10.1039/C5RA00004A","ISSN":"2046-2069","issue":"26","journalAbbreviation":"RSC Adv.","language":"en","note":"publisher: The Royal Society of Chemistry","page":"20617-20622","source":"pubs.rsc.org","titl

Name	Ea_H$^+$ (eV)	Ea_H_2 (eV)	Front view (YZ)	Top view (XY)	Information (ID, Name, Pore size, dma, Electron affinity, band gap, Neighbour atom)	Ref
						e":"Stability of hydrogenated graphene: a first-principles study","title-short":"Stability of hydrogenated graphene","URL":"https://pubs.rsc.org/en/content/articlelanding/2015/ra/c5ra00004a","volume":"5","author":[{"family":"Yi","given":"Ding"},{"family":"Yang","given":"Liu"},{"family":"Xie","given":"Shijie"},{"family":"Saxena","given":"Avadh"}],"accessed":{"date-parts":[["2024",12,1]]},"issued":{"date-parts":[["2015",2,17]]}}}],"schema":"https://

Name	Ea_H$^+$ (eV)	Ea_H_2 (eV)	Front view (YZ)	Top view (XY)	Information (ID, Name, Pore size, dma, Electron affinity, band gap, Neighbour atom)	Ref
						github.com/citation -style-language /schema/raw/mast EXPe[42–45] PErM
h-BN	0.69	12.63	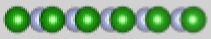	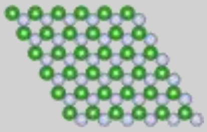	60ef72d03b482494c46302d5, Hexagonal Boron Nitride, 1.59Å^2, 1.45 Å, -0.56 eV, 4.66 eV, N	D F T ADDIN ZOTERO_ITEM CSL_CITATION {"citationID":"RHIVFLfC","properties":{"formattedCitation":"\\super 46\\nosupersub{}","plainCitation":"46","noteIndex":0},"citationItems":[{"id":2118,"uris":["http://zotero.org/users/15250265/items/WBNFZV7X"],"itemData":{"id":2118,"type":"article-journal","abstract":"The hydrogen storage capacity of Boron Nitride nanoshee

Name	Ea_H$^+$ (eV)	Ea_H_2 (eV)	Front view (YZ)	Top view (XY)	Information (ID, Name, Pore size, dma, Electron affinity, band gap, Neighbour atom)	Ref
						d by using density functional theory (DFT). All the structural and electronic properties of a monolayer BN nanosheet are in well agreement with the previously reported results. Out of the four possible adsorption sites, centre is the most favourable adsorption site for H2 molecule with binding energy ~ 0.212 eV/H2. We have proceeded our calculations considering this adsorption site. The calculated direct band gap

Name	Ea_H⁺ (eV)	Ea_H₂ (eV)	Front view (YZ)	Top view (XY)	Information (ID, Name, Pore size, dma, Electron affinity, band gap, Neighbour atom)	Ref
						within GGA and HSE for pristine h-BN monolayer are found to be 4.669 eV and 5.63 eV, respectively. In our calculation the Hydrogen storage capacity of BN nanosheet was found to be 6.7 wt.% well within benchmark value (6.0%) with an average adsorption energy of (~ 0.128 eV/H2). Bader analysis revealed that the charge transfer from BN nanosheet to the H2 molecule is very low (0.004–0.065 ∣e∣) leading to weak

Name	Ea_H^+ (eV)	Ea_H_2 (eV)	Front view (YZ)	Top view (XY)	Information (ID, Name, Pore size, dma, Electron affinity, band gap, Neighbour atom)	Ref
						binding of the H2 molecule. The calculated desorption temperature was found to be low due to low average adsorption energy of the H2 molecule. Also, upon increasing the number of H2 molecule adsorption a feeble tuning of the band gap has been observed due to the contribution of the 1s orbital of H2 molecule.","container-title":"Surfaces and Interfaces","DOI":"10.1016/j.surfin.2021.101043","ISSN":"24680230","journalAbbrevi

Name	Ea_H^+ (eV)	Ea_H_2 (eV)	Front view (YZ)	Top view (XY)	Information (ID, Name, Pore size, dma, Electron affinity, band gap, Neighbour atom)	Ref
						ation":"Surfaces and Interfaces","language":"en","page":"101043","source":"DOI.org (Crossref)","title":"Hexagonal boron nitride (h -BN) nanosheet as a potential hydrogen adsorption material: A density functional theory (DFT) study","title-short":"Hexagonal boron nitride (h -BN) nanosheet as a potential hydrogen adsorption material","URL":"https://linkinghub.elsevier.com/retrieve/pii/S2468023021001206","volume":"24","author":[{"family":"Chettri","given":"B."},

Name	Ea_H$^+$ (eV)	Ea_H$_2$ (eV)	Front view (YZ)	Top view (XY)	Information (ID, Name, Pore size, dma, Electron affinity, band gap, Neighbour atom)	Ref
						{"family":"Patra","given":"P.K."},{"family":"Hieu","given":"Nguyen N."},{"family":"Rai","given":"D.P."}],"accessed":{"date-parts":[["2024",12,1]]},"issued":{"date-parts":[["2021",6]]}}}],"schema":"https://github.com/citation-style-language/schema/raw/maste EXP[47–49] PEM[50–52]
PO_2	1.17	10.22	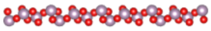	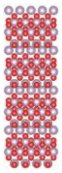	60ef71ff4dbb56fa22f3ceed, Phosphorus Dioxide, 0.82Å^2, 1.55Å, 1.22eV, 3.31 eV, P	DFT ADDIN ZOTERO_ITEM CSL_CITATION {"citationID":"BrLcGRCP","properties":{"formattedCitation":"\\super 53\\nosupersub{}","plainCitation":"53","noteIndex":0

Name	Ea_H⁺ (eV)	Ea_H_2 (eV)	Front view (YZ)	Top view (XY)	Information (ID, Name, Pore size, dma, Electron affinity, band gap, Neighbour atom)	Ref
						0,"uris":["http://zotero.org/users/15250265/items/5WEHTGIG"],"itemData":{"id":2120,"type":"article-journal","abstract":"Phosphorene is a rising star in electronics. Recently, 2D phosphorus oxides with higher stability have been synthesized. In this study, we theoretically explored the structures and properties of 2D phosphorus oxides. We found that the structural features of PxOy vary with the oxygen content. When the oxygen

Name	Ea_H+ (eV)	Ea_H_2 (eV)	Front view (YZ)	Top view (XY)	Information (ID, Name, Pore size, dma, Electron affinity, band gap, Neighbour atom)	Ref
						content is low, the most stable PxOy material can be obtained by the adsorption of O atoms on phosphorene. Otherwise, stable structures are no longer based on phosphorene and will contain P–O–P motifs. We found that P4O4 has a direct band gap (about 2.24 eV), good optical absorption, and high stability in water, so it may be suitable for photochemical water splitting. P2O3 adopts two possible stable ferroelectric

Name	Ea_H$^+$ (eV)	Ea_H_2 (eV)	Front view (YZ)	Top view (XY)	Information (ID, Name, Pore size, dma, Electron affinity, band gap, Neighbour atom)	Ref
						structures (P2O3-I and P2O3-II) with electric polarization perpendicular and parallel to the lateral plane, respectively, as the lowest-energy configurations, depending on the layer thickness. We propose that P2O3 could be used in novel nanoscale multiple-state memory devices.","container-title":"Angewandte Chemie International Edition","DOI":"10.1002/anie.201602295","ISSN":"1521-3773","issue":"30","languag

Name	Ea_H$^+$ (eV)	Ea_H_2 (eV)	Front view (YZ)	Top view (XY)	Information (ID, Name, Pore size, dma, Electron affinity, band gap, Neighbour atom)	Ref
						e":"en","license":"© 2016 WILEY-VCH Verlag GmbH & Co. KGaA, Weinheim","note":"_eprint: https://onlinelibrary.wiley.com/doi/pdf/10.1002/anie.201602295","page":"8575-8580","source":"Wiley Online Library","title":"Two-Dimensional Phosphorus Oxides as Energy and Information Materials","URL":"https://onlinelibrary.wiley.com/doi/abs/10.1002/anie.201602295","volume":"55","author":[{"family":"Luo","given":"Wei"},{"family":"

Name	Ea_H$^+$ (eV)	Ea_H_2 (eV)	Front view (YZ)	Top view (XY)	Information (ID, Name, Pore size, dma, Electron affinity, band gap, Neighbour atom)	Ref
						Xiang","given":"Hongjun"}],"accessed":{"date-parts":[["2024",12,1]]},"issued":{"date-parts":[["2016"]]}}}],"schema":"https://github.com/citation-style-language/schema/raw/maste E X P ADDIN ZOTERO_ITEM CSL_CITATION {"citationID":"eNYbQzFN","properties":{"formattedCitation":"\\super 53\\nosupersub{}","plainCitation":"53","noteIndex":0},"citationItems":[{"id":2120,"uris":["http://zotero.org/users/15250265/items/5WEHTGIG"],"itemDa

Name	Ea_H⁺ (eV)	Ea_H_2 (eV)	Front view (YZ)	Top view (XY)	Information (ID, Name, Pore size, dma, Electron affinity, band gap, Neighbour atom)	Ref
						le-journal", "abstract ":"Phosphorene is a rising star in electronics. Recently, 2D phosphorus oxides with higher stability have been synthesized. In this study, we theoretically explored the structures and properties of 2D phosphorus oxides. We found that the structural features of PxOy vary with the oxygen content. When the oxygen content is low, the most stable PxOy material can be obtained by the adsorption of O

Name	Ea_H^+ (eV)	Ea_H_2 (eV)	Front view (YZ)	Top view (XY)	Information (ID, Name, Pore size, dma, Electron affinity, band gap, Neighbour atom)	Ref
						atoms on phosphorene. Otherwise, stable structures are no longer based on phosphorene and will contain P–O–P motifs. We found that P4O4 has a direct band gap (about 2.24 eV), good optical absorption, and high stability in water, so it may be suitable for photochemical water splitting. P2O3 adopts two possible stable ferroelectric structures (P2O3-I and P2O3-II) with electric polarization on perpendicular and parallel

Name	Ea_H^{+} (eV)	Ea_H_2 (eV)	Front view (YZ)	Top view (XY)	Information (ID, Name, Pore size, dma, Electron affinity, band gap, Neighbour atom)	Ref
						to the lateral plane, respectively, as the lowest-energy configurations, depending on the layer thickness. We propose that P2O3 could be used in novel nanoscale multiple-state memory devices.","container-title":"Angewandte Chemie International Edition","DOI":"10.1002/anie.201602295","ISSN":"1521-3773","issue":"30","language":"en","license":"© 2016 WILEY-VCH Verlag GmbH & Co. KGaA, Weinheim","note

Name	Ea_H⁺ (eV)	Ea_H₂ (eV)	Front view (YZ)	Top view (XY)	Information (ID, Name, Pore size, dma, Electron affinity, band gap, Neighbour atom)	Ref
						":"_eprint: https://onlinelibrary.wiley.com/doi/pdf/10.1002/anie.201602295","page":"8575-8580","source":"Wiley Online Library","title":"Two-Dimensional Phosphorus Oxides as Energy and Information Materials","URL":"https://onlinelibrary.wiley.com/doi/abs/10.1002/anie.201602295","volume":"55","author":[{"family":"Luo","given":"Wei"},{"family":"Xiang","given":"Hongjun"}],"accessed":{"date-parts":[["2024",12,1]]},"issued":{"date-

Name	Ea_H$^+$ (eV)	Ea_H$_2$ (eV)	Front view (YZ)	Top view (XY)	Information (ID, Name, Pore size, dma, Electron affinity, band gap, Neighbour atom)	Ref
						parts":[["2016"]]}}}],"schema":"https://github.com/citation-style-language/schema/raw/maste e PEM
C	1.33	13.18	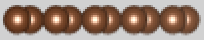	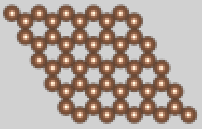	60ef72924dbb56fa22f3d4b2, Graphene, 1.56Å^2, 1.42 Å, 1.26eV, 0 eV, C	D F T ADDIN ZOTERO_ITEM CSL_CITATION {"citationID":"CdkQPjwI","properties":{"formattedCitation":"\\super 54\\nosupersub{}","plainCitation":"54","noteIndex":0},"citationItems":[{"id":2125,"uris":["http://zotero.org/users/15250265/items/U8725XLV"],"itemData":{"id":2125,"type":"article-journal","abstract":"Two-dimensional (2D) monolay

Name	Ea_H^+ (eV)	Ea_H_2 (eV)	Front view (YZ)	Top view (XY)	Information (ID, Name, Pore size, dma, Electron affinity, band gap, Neighbour atom)	Ref
						be exploited as the thinnest membrane with distinct differential sieving properties for proton isotopes. Motivated from the experimental evidence of differential sieving proton isotopes through graphene and hexagonal boron nitrate (h-BN) monolayer, we compute the kinetic barrier of isotope H+ and D+ permeation through model graphene and h-BN fragments at the MP2/6-31++G(d,p) level of theory. On the basis of the ratio

Name	Ea_H⁺ (eV)	Ea_H₂ (eV)	Front view (YZ)	Top view (XY)	Information (ID, Name, Pore size, dma, Electron affinity, band gap, Neighbour atom)	Ref
						of tunneling reaction rate constant, the isotope separation ratio of H+/D+ and H+/T+ is predicted to be ~12 and 37, respectively. The tunneling reaction rate constant can be estimated from the zero-point-energy computed at the transition state for the proton isotope permeation though the 2D model systems. We show that the presence of Stone–Wales (55–77) defect in the model graphene fragment can significantly lower the

Name	Ea_H$^+$ (eV)	Ea_H$_2$ (eV)	Front view (YZ)	Top view (XY)	Information (ID, Name, Pore size, dma, Electron affinity, band gap, Neighbour atom)	Ref
						proton permeation barrier by 0.55 eV. With the defect, the ratio of tunneling reaction rate constant of H+/D+ is increased to ~25. In addition to model graphene and h-BN, we have examined proton permeation capability of α-boron monolayer. We compute the tunneling reaction pathway for H+ through α-boron monolayer using both the climbing nudged elastic band (c-NEB) method and the scanning-path method. Both

Name	Ea_H⁺ (eV)	Ea_H_2 (eV)	Front view (YZ)	Top view (XY)	Information (ID, Name, Pore size, dma, Electron affinity, band gap, Neighbour atom)	Ref
						methods suggest that α-boron monolayer entails a relatively low barrier of ~0.20 eV for H+ permeation, much lower than that of the model graphene and h-BN fragments. Our studies provide molecular-level insights into the differential permeation of proton isotopes through 2D materials. The methods can be extended to examine isotope separation capability of other 2D materials as well.","container-title":"The Journal

Name	Ea_H^+ (eV)	Ea_H_2 (eV)	Front view (YZ)	Top view (XY)	Information (ID, Name, Pore size, dma, Electron affinity, band gap, Neighbour atom)	Ref
						of Physical Chemistry Letters","DOI":"10.1021/acs.jpclett.6b01507","issue":"17","journalAbbreviation":"J. Phys. Chem. Lett.","note":"publisher: American Chemical Society","page":"3395-3400","source":"ACS Publications","title":"Differential Permeability of Proton Isotopes through Graphene and Graphene Analogue Monolayer","URL":"https://doi.org/10.1021/acs.jpclett.6b01507","volume":"7","author":[{"family":"Zhang","given":"Qiuju"}

Name	Ea_H$^+$ (eV)	Ea_H_2 (eV)	Front view (YZ)	Top view (XY)	Information (ID, Name, Pore size, dma, Electron affinity, band gap, Neighbour atom)	Ref
						,{"family":"Ju","given":"Minggang"},{"family":"Chen","given":"Liang"},{"family":"Zeng","given":"Xiao Cheng"}],"accessed":{"date-parts":[["2024",12,1]]},"issued":{"date-parts":[["2016",9,1]]}}}],"schema":"https://github.com/citation-style-language/schema/raw/mast E X P ADDIN ZOTERO_ITEM CSL_CITATION {"citationID":"IHgLeNmc","properties":{"formattedCitation":"\\super 55\\nosupersub{}","plainCitation":"55","noteIndex":0},"citatio

Name	Ea_H+ (eV)	Ea_H_2 (eV)	Front view (YZ)	Top view (XY)	Information (ID, Name, Pore size, dma, Electron affinity, band gap, Neighbour atom)	Ref
						"http://zotero.org/users/15250265/items/86C4IQ8B"],"itemData":{"id":1954,"type":"article-journal","abstract":"Since the isolation of graphene in 2004, two-dimensional (2D) materials research has rapidly evolved into an entire subdiscipline in the physical sciences with a wide range of emergent applications. The unique 2D structure offers an open canvas to tailor and functionalize 2D materials through layer number, defects, morphology,

Name	Ea_H$^+$ (eV)	Ea_H_2 (eV)	Front view (YZ)	Top view (XY)	Information (ID, Name, Pore size, dma, Electron affinity, band gap, Neighbour atom)	Ref
						moiré pattern, strain, and other control knobs. Through this review, we aim to highlight the most recent discoveries in the following topics: theory-guided synthesis for enhanced control of 2D morphologies, quality, yield, as well as insights toward novel 2D materials; defect engineering to control and understand the role of various defects, including in situ and ex situ methods; and properties and applications that are related to

Name	Ea_H⁺ (eV)	Ea_H_2 (eV)	Front view (YZ)	Top view (XY)	Information (ID, Name, Pore size, dma, Electron affinity, band gap, Neighbour atom)	Ref
						moiré engineering, strain engineering, and artificial intelligence. Finally, we also provide our perspective on the challenges and opportunities in this fascinating field.","container-title":"ACS Nanoscience Au","DOI":"10.1021/acsnanoscienceau.2c00017","issue":"6","journalAbbreviation":"ACS Nanosci. Au","note":"publisher: American Chemical Society","page":"450-485","source":"ACS Publications","title":"Graphene and Beyond:

Name	Ea_H⁺ (eV)	Ea_H_2 (eV)	Front view (YZ)	Top view (XY)	Information (ID, Name, Pore size, dma, Electron affinity, band gap, Neighbour atom)	Ref
						Recent Advances in Two-Dimensional Materials Synthesis, Properties, and Devices","title-short":"Graphene and Beyond","URL":"https://doi.org/10.1021/acsnanoscienceau.2c00017","volume":"2","author":[{"family":"Lei","given":"Yu"},{"family":"Zhang","given":"Tianyi"},{"family":"Lin","given":"Yu-Chuan"},{"family":"Granzier-Nakajima","given":"Tomotaroh"},{"family":"Bepete","given":"George"},{"family":"Kowalczyk","given":"Dorota A."},{"family":"Li

Name	Ea_H+ (eV)	Ea_H2 (eV)	Front view (YZ)	Top view (XY)	Information (ID, Name, Pore size, dma, Electron affinity, band gap, Neighbour atom)	Ref
						n","given":"Zhong"},{"family":"Zhou","given":"Da"},{"family":"Schranghamer","given":"Thomas F."},{"family":"Dodda","given":"Akhil"},{"family":"Sebastian","given":"Amritanand"},{"family":"Chen","given":"Yifeng"},{"family":"Liu","given":"Yuanyue"},{"family":"Pourtois","given":"Geoffrey"},{"family":"Kempa","given":"Thomas J."},{"family":"Schuler","given":"Bruno"},{"family":"Edmonds","given":"Mark T."},{"family":"Quek","given":"Su Ying"},{"family":"Wurstba

Name	Ea_H^{+} (eV)	Ea_H_2 (eV)	Front view (YZ)	Top view (XY)	Information (ID, Name, Pore size, dma, Electron affinity, band gap, Neighbour atom)	Ref
						uer","given":"Ursula"},{"family":"Wu","given":"Stephen M."},{"family":"Glavin","given":"Nicholas R."},{"family":"Das","given":"Saptarshi"},{"family":"Dash","given":"Saroj Prasad"},{"family":"Redwing","given":"Joan M."},{"family":"Robinson","given":"Joshua A."},{"family":"Terrones","given":"Mauricio"}],"accessed":{"date-parts":[["2024",11,17]]},"issued":{"date-parts":[["2022",12,21]]}}}],"schema":"https://github.com/citation-style-language/schema/

Name	Ea_H+ (eV)	Ea_H_2 (eV)	Front view (YZ)	Top view (XY)	Information (ID, Name, Pore size, dma, Electron affinity, band gap, Neighbour atom)	Ref
						PEM[56–58]

Table S4. **The hyper-parameters of DNN, RF, and GP algorithms for classification (C) and regression (R) tasks**.

Models	Parameters
DNN	512-128-64-32 (ReLU), L2(λ=0.01), Dropout(0.1), Adam, MSE, MAE, epochs: 200, batch: 50
GP	Kernel: C(1.0,[10^{-3},10^{3}])×Matern(1.0,ν=0.4,[10^{-3},10^{3}])+WhiteKernel(1.0)C(1.0, [10^{-3},10^{3}]), Restarts: 20, Random State: 42
RF	max_depth=9, min_samples_split=5, n_estimators=250, random_state=120

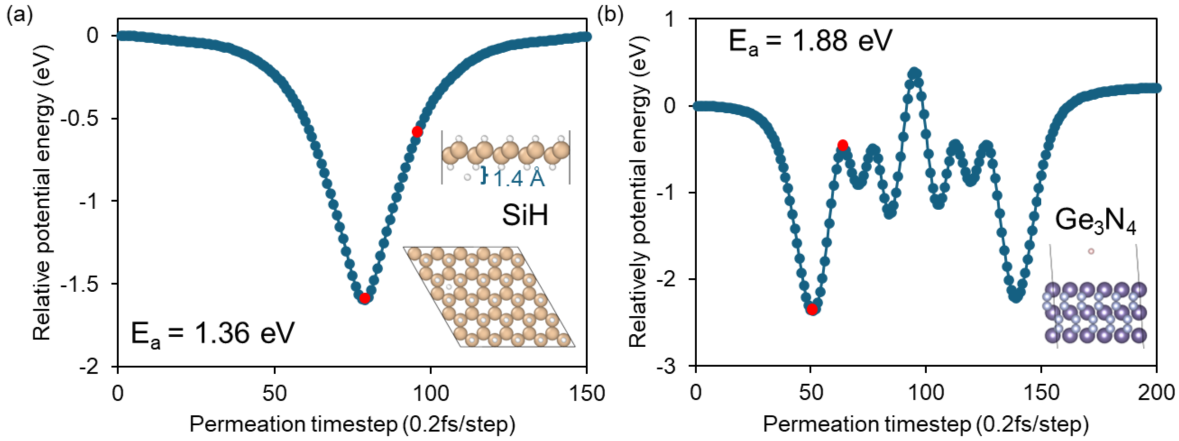

Figure S1. The relative potential energy profile of proton permeation for three different materials as examples of obtaining permeation barrier through AIMD, the red points indicate the maximum and minimum energy points to calculate proton permeation barriers.

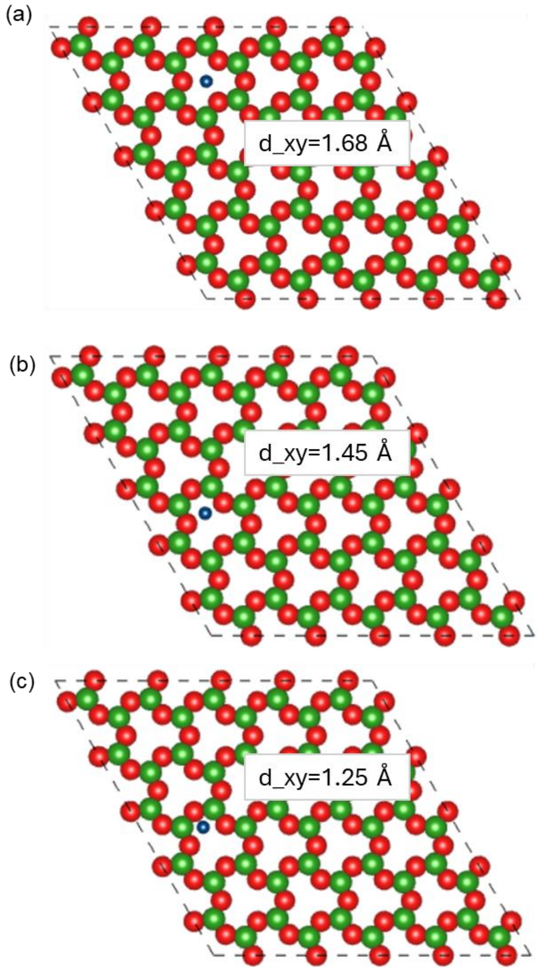

Figure S2. Representative initial proton placement configurations above the 2D layers from t
o
p

v
i
e
w

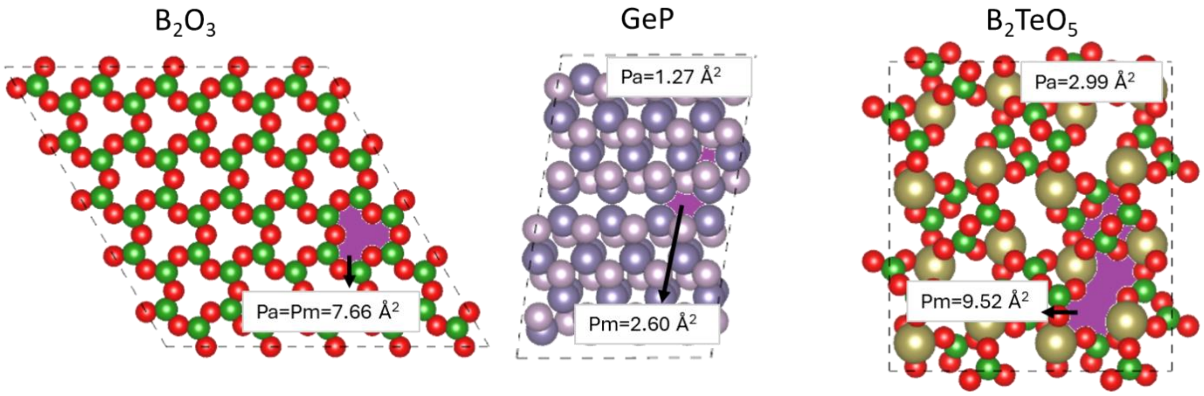

Figure S3. [illegible] atom in the 2D sheet

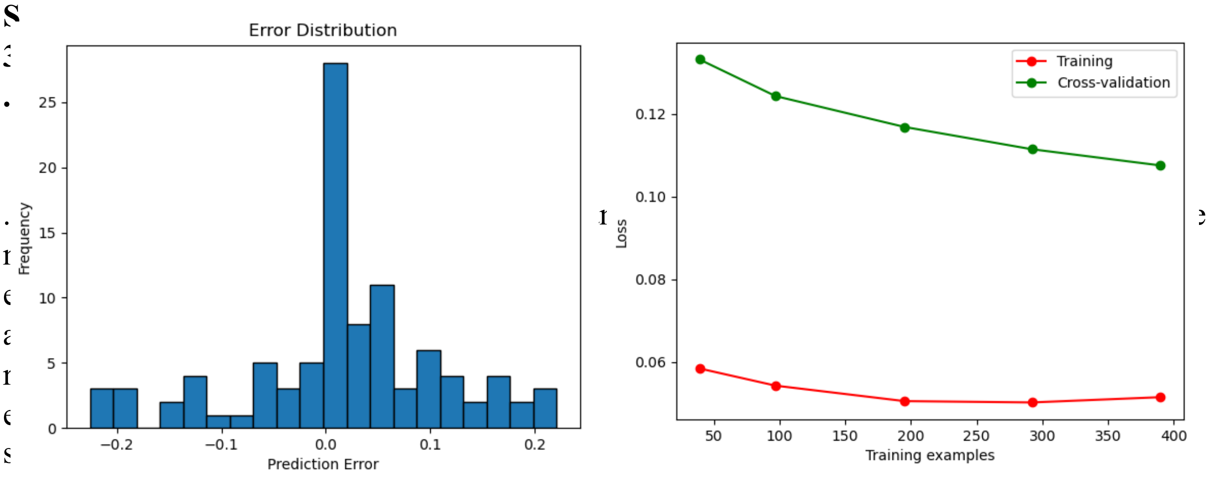

Figure S4. (a) The histogram of prediction errors for the Random Forest Regressor model. The prediction error is defined as the difference between the predicted and actual values. The x-axis represents the range of prediction errors, while the y-axis indicates the frequency of these errors. **(b)** Learning curve of training and cross-validation loss for the Random Forest Regressor model, showing the training loss (red curve) and cross-validation loss (green curve) as functions of the number of training samples.

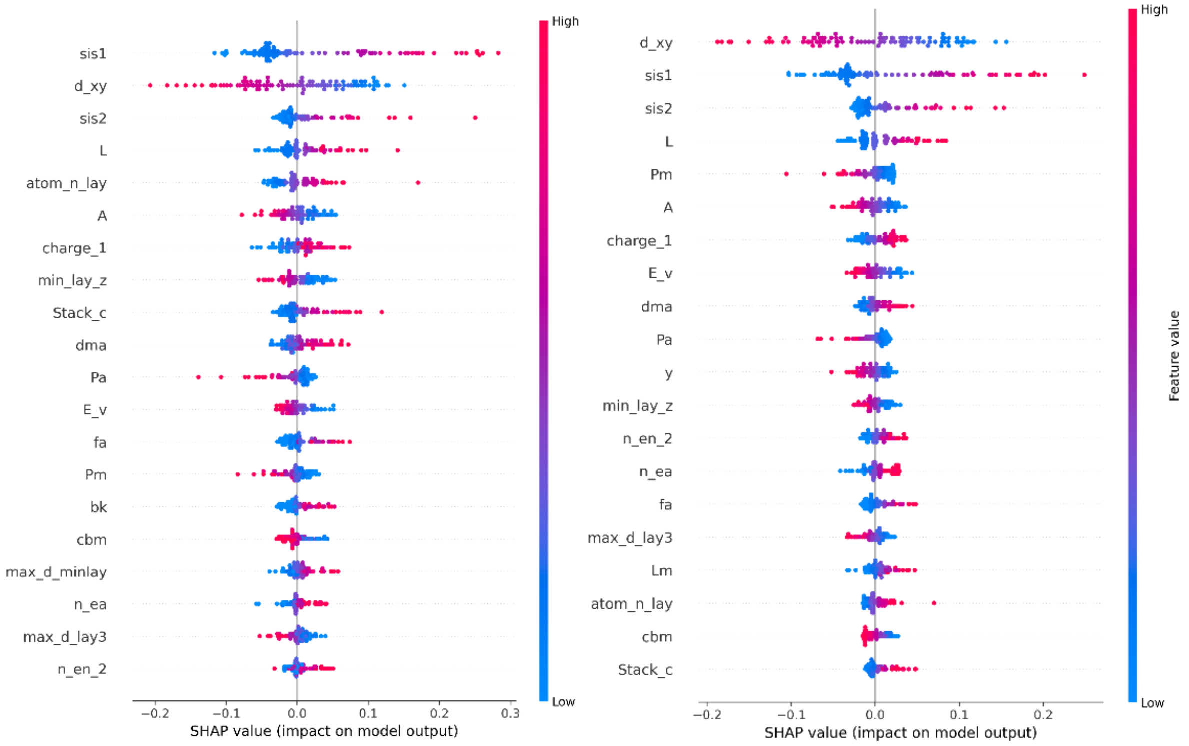

Figure S5. Shapley Additive Explanations (SHAP) value analysis showing feature impact on DNN (left) and GPR (right) model output. The SHAP value quantifies the feature's impact on the model's prediction of proton permeation barrier. Positive SHAP values indicate that a feature increases the predicted value, whereas negative SHAP values indicate a decrease in the predicted value. The color of each point represents the magnitude of the feature value for each sample, blue indicates low feature values, red indicates high feature values.

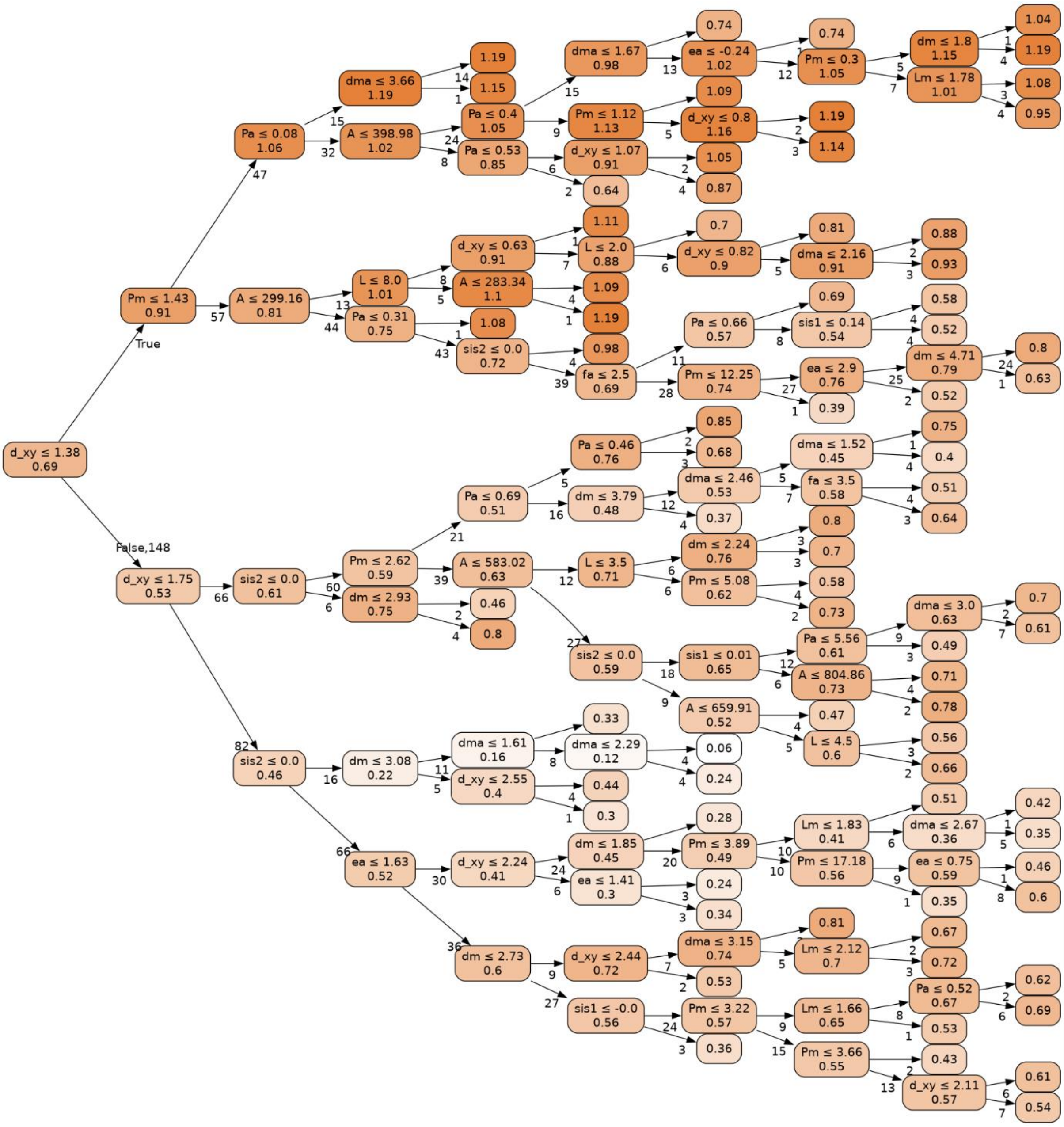
d_xy ≤ 1.38
0.69
True
False,148
Pm ≤ 1.43
0.91
Pa ≤ 0.08
1.06
dma ≤ 3.66
1.19
1.19
1.15
A ≤ 398.98
1.02
Pa ≤ 0.4
1.05
dma ≤ 1.67
0.98
0.74
ea ≤ -0.24
1.02
0.74
Pm ≤ 0.3
1.05
dm ≤ 1.8
1.15
1.04
1.19
Lm ≤ 1.78
1.01
1.08
0.95
Pm ≤ 1.12
1.13
1.09
d_xy ≤ 0.8
1.16
1.19
1.14
Pa ≤ 0.53
0.85
d_xy ≤ 1.07
0.91
1.05
0.87
0.64
A ≤ 299.16
0.81
L ≤ 8.0
1.01
d_xy ≤ 0.63
0.91
1.11
L ≤ 2.0
0.88
0.7
d_xy ≤ 0.82
0.9
0.81
dma ≤ 2.16
0.91
0.88
0.93
A ≤ 283.34
1.1
1.09
1.19
Pa ≤ 0.31
0.75
1.08
sis2 ≤ 0.0
0.72
0.98
fa ≤ 2.5
0.69
Pa ≤ 0.66
0.57
0.69
sis1 ≤ 0.14
0.54
0.58
0.52
Pm ≤ 12.25
0.74
ea ≤ 2.9
0.76
dm ≤ 4.71
0.79
0.8
0.63
0.52
0.39
d_xy ≤ 1.75
0.53
sis2 ≤ 0.0
0.61
Pm ≤ 2.62
0.59
Pa ≤ 0.69
0.51
Pa ≤ 0.46
0.76
0.85
0.68
dm ≤ 3.79
0.48
dma ≤ 2.46
0.53
dma ≤ 1.52
0.45
0.75
0.4
fa ≤ 3.5
0.58
0.51
0.64
0.37
A ≤ 583.02
0.63
L ≤ 3.5
0.71
dm ≤ 2.24
0.76
0.8
0.7
Pm ≤ 5.08
0.62
0.58
0.73
sis2 ≤ 0.0
0.59
sis1 ≤ 0.01
0.65
Pa ≤ 5.56
0.61
dma ≤ 3.0
0.63
0.7
0.61
0.49
A ≤ 804.86
0.73
0.71
0.78
A ≤ 659.91
0.52
0.47
L ≤ 4.5
0.6
0.56
0.66
dm ≤ 2.93
0.75
0.46
0.8
sis2 ≤ 0.0
0.46
dm ≤ 3.08
0.22
dma ≤ 1.61
0.16
0.33
dma ≤ 2.29
0.12
0.06
0.24
d_xy ≤ 2.55
0.4
0.44
0.3
ea ≤ 1.63
0.52
d_xy ≤ 2.24
0.41
dm ≤ 1.85
0.45
0.28
Pm ≤ 3.89
0.49
Lm ≤ 1.83
0.41
0.51
dma ≤ 2.67
0.36
0.42
0.35
Pm ≤ 17.18
0.56
ea ≤ 0.75
0.59
0.46
0.6
0.35
ea ≤ 1.41
0.3
0.24
0.34
dm ≤ 2.73
0.6
d_xy ≤ 2.44
0.72
dma ≤ 3.15
0.74
0.81
Lm ≤ 2.12
0.7
0.67
0.72
0.53
sis1 ≤ -0.0
0.56
Pm ≤ 3.22
0.57
Lm ≤ 1.66
0.65
Pa ≤ 0.52
0.67
0.62
0.69
0.53
Pm ≤ 3.66
0.55
0.43
d_xy ≤ 2.11
0.57
0.61
0.54
0.36

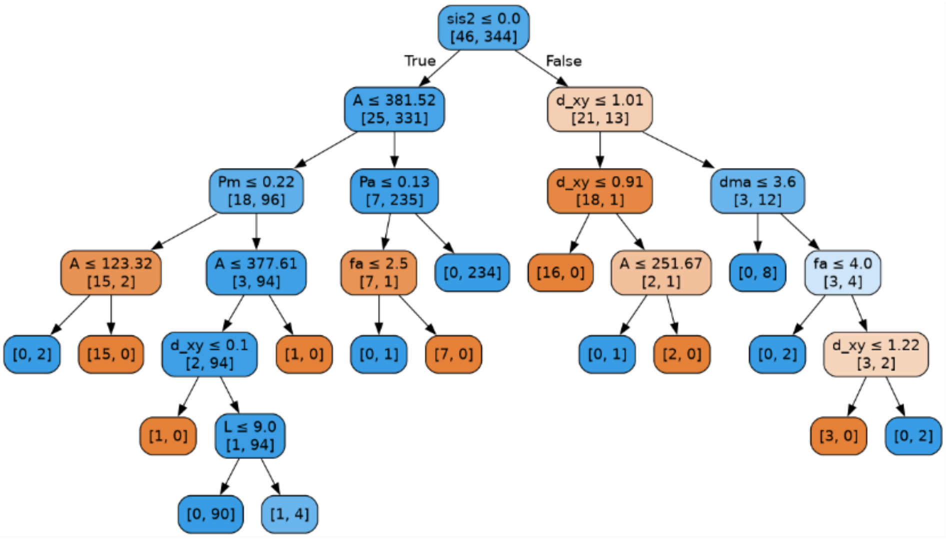
sis2 ≤ 0.0
[46, 344]
True
False
A ≤ 381.52
[25, 331]
Pm ≤ 0.22
[18, 96]
A ≤ 123.32
[15, 2]
[0, 2]
[15, 0]
A ≤ 377.61
[3, 94]
d_xy ≤ 0.1
[2, 94]
[1, 0]
L ≤ 9.0
[1, 94]
[0, 90]
[1, 4]
[1, 0]
Pa ≤ 0.13
[7, 235]
fa ≤ 2.5
[7, 1]
[0, 1]
[7, 0]
[0, 234]
d_xy ≤ 1.01
[21, 13]
d_xy ≤ 0.91
[18, 1]
[16, 0]
A ≤ 251.67
[2, 1]
[0, 1]
[2, 0]
dma ≤ 3.6
[3, 12]
[0, 8]
fa ≤ 4.0
[3, 4]
[0, 2]
d_xy ≤ 1.22
[3, 2]
[3, 0]
[0, 2]

Figure S6. (a) Visualization of the trained Random Forest Regressor (RFR) to highlight the key decision pathways of the model. Each node represents a split based on a specific feature and threshold, displayed on the first line. The second line indicates the predicted mean value of samples in the node, the values displayed here are normalized (e.g., 0.65 corresponds to the barrier of 1.4 eV). The nodes in the last branch represent the final predicted values, while the intermediate levels of the tree represent the average predicted values at each node. The left arrows represent the True condition for the specified feature threshold, while the right arrows represent the False condition. The numbers beside the arrows indicate the sample size passing through each branch. Node colors correspond to the predicted value, with darker shades representing higher values and lighter shades representing lower ones. Nodes at higher levels split the data based on features with significant influence, while lower-level nodes focus on finer adjustments to the predictions. The abbreviations of the features can be found in **Table S1**. **(b)** Decision tree visualization from a random forest classifier. The top line in each node indicates the splitting condition based on a feature and its threshold. The numbers in brackets in the second line represent the sample distribution between the two classes, [proton permeable, proton impermeable]. The blue nodes represent the classification of proton permeable, while orange nodes favor the classification of impermeable, with darker shade reflecting higher certainty. The last leaf nodes, representing the final predictions, show the number of samples classified into each class.

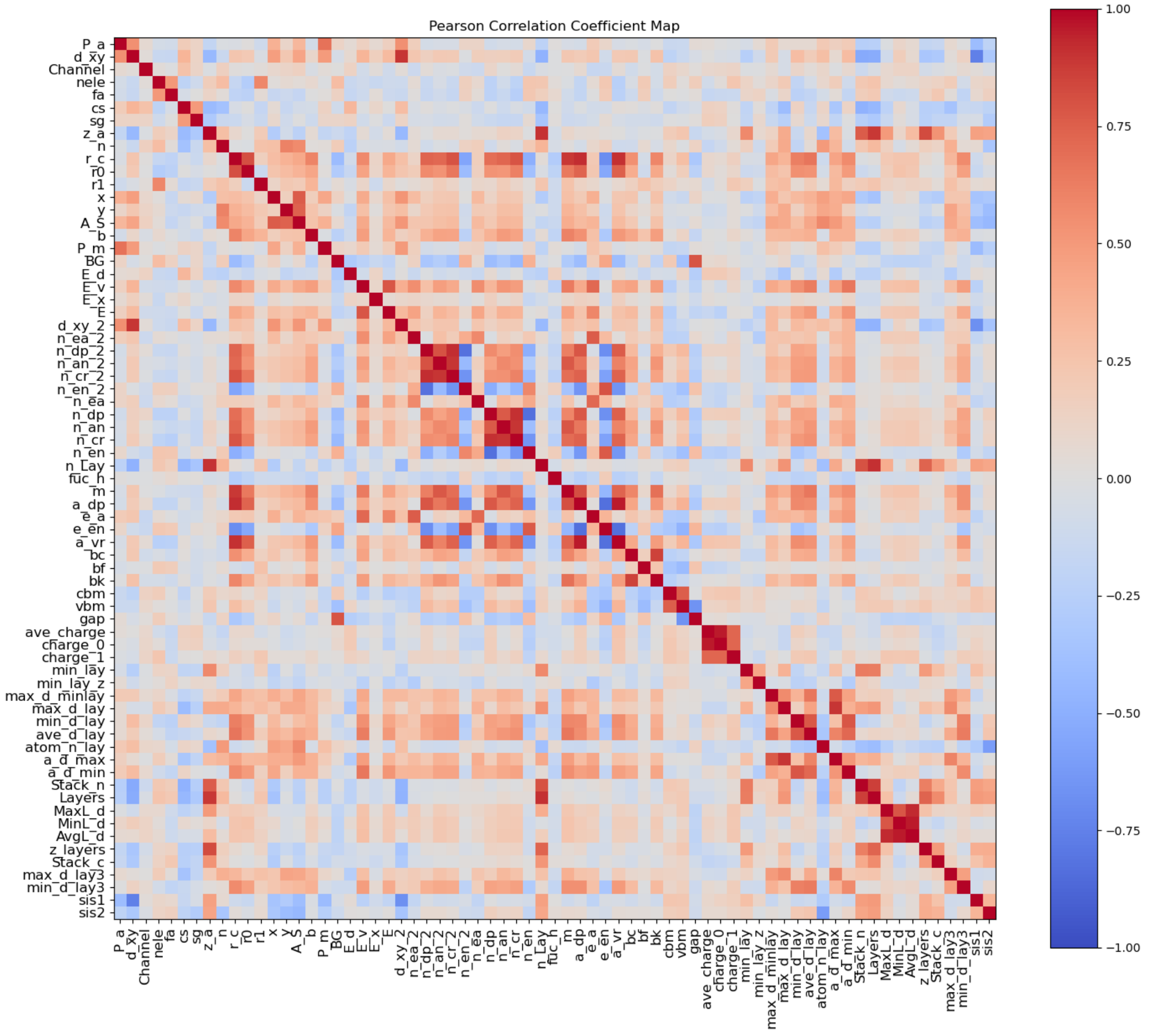

Figure S7. Pearson correlation coefficient map for selected features. Each cell represents the correlation value between a pair of features, ranging from -1 (perfect negative correlation) to +1 (perfect positive correlation).

REFERENCES

; Qiao, Z. Machine-Learning-Assisted High-Throughput Computational Screening of

(41), 22471–22478. https://doi.org/10.1021/acs.jpcc.0c05964.

(1), 1–7. https://doi.org/10.1038/s41524-017-0045-8.
(28), 32134–32148. https://doi.org/10.1021/acsami.2c08977.
280–290. https://doi.org/10.1016/j.commatsci.2017.12.022.
366–383. https://doi.org/10.1016/j.nanoen.2015.08.025.

(3), 1265–1271. https://doi.org/10.1039/C7NR08172C.
(1), 113–118. https://doi.org/10.1021/nl203065e.
115–150. https://doi.org/10.1016/j.ensm.2020.07.006.
(7), 2982–2996. https://doi.org/10.1039/D2NR05809J.
323–334. https://doi.org/10.1016/j.jechem.2021.04.061.

(46), 16318–16325. https://doi.org/10.1039/D0TC03892J.
(38), 13405–13410. https://doi.org/10.1002/anie.201908377.

(5), 4414–4421. https://doi.org/10.1021/nn4009406.
(12), 5032–5039. https://doi.org/10.1039/C8TA00129D.

(63), 38227–38232. https://doi.org/10.1039/D0RA07539F.
, pp 1–63. https://doi.org/10.1016/S0065-2792(08)60070-9.

(2), 314–320. https://doi.org/10.1016/0022-2852(71)90301-8.
(38), 15578–15587. https://doi.org/10.1039/D4TC02208D.
(2), 1110–1117. https://doi.org/10.1021/acs.nanolett.5b04341.
(32) Huang, Z.; Ren, K.; Zheng, R.; Wang, L.; Wang, L. Ultrahigh Carrier Mobility in Two-Dimensional IV–VI Semiconductors for Photocatalytic Water Splitting. *Molecules* **2023**,

(35) Koch, E.-C.; Suceska, M. Analysis of the Explosive Properties of Tetrasulfur Tetranitride,

78–83. https://doi.org/10.1016/j.cplett.2017.01.013.

(26), 20617–20622. https://doi.org/10.1039/C5RA00004A.

(8017), 619–624. https://doi.org/10.1038/s41586-024-07435-8.

(9), 9104–9112. https://doi.org/10.1021/acsanm.1c01691.
(4), 262–266. https://doi.org/10.1038/nphoton.2015.277.
T.-Y.; Shin, H. S. AA′-Stacked Trilayer Hexagonal Boron Nitride Membrane for Proton

(6), 2898–2912. https://doi.org/10.1039/C9TA12293A.
(27), 12755–12773. https://doi.org/10.1039/C9NR03094H.
(30), 8575–8580. https://doi.org/10.1002/anie.201602295.
(17), 3395–3400. https://doi.org/10.1021/acs.jpclett.6b01507.
(6), 450–485. https://doi.org/10.1021/acsnanoscienceau.2c00017.
(18), [illegible] https://doi.org/10.1021/acsami.[illegible]c01254.
(57) Pavko, L.; Gatalo, M.; Finšgar, M.; Ruiz-Zepeda, F.; Ehelebe, K.; Kaiser, P.; Geuß, M.; Đukić, T.; Surca, A. K.; Šala, M.; Bele, M.; Cherevko, S.; Genorio, B.; Hodnik, N.; Gaberšček, M. Graphene-Derived Carbon Support Boosts Proton Exchange Membrane

(7), 958–983. https://doi.org/10.1002/ese3.833.
